# Diagnosing ADHD in adults in randomized controlled studies: a scoping review

**DOI:** 10.1192/j.eurpsy.2025.2447

**Published:** 2025-04-14

**Authors:** Igor Studart, Mads Gram Henriksen, Julie Nordgaard

**Affiliations:** 1 Institute of Psychiatry, University of São Paulo, Sao Paulo, Brazil; 2 Center for Subjectivity Research, University of Copenhagen, Copenhagen, Denmark; 3 Psychiatry East, Region Zealand, Roskilde, Denmark; 4 Department of Clinical Medicine, University of Copenhagen, Copenhagen, Denmark

**Keywords:** comorbidity, diagnostic criteria, methodology, psychopathology

## Abstract

**Background:**

The diagnosis of ADHD in adults is on the rise. Applying the ADHD diagnosis, which originally was described in children, to adults has involved a “subjectivization” of some of the diagnostic criteria, i.e., some behavioral features (signs) in children have become experiences (symptoms) in adults. These issues raise the question of how ADHD is best diagnosed in adults? Thus, we examined how ADHD is diagnosed in adults in research.

**Methods:**

A review of how ADHD is diagnosed in adults in randomized controlled studies (RCTs).

**Results:**

We include 292 RCTs. We found substantial variation and no consensus about the diagnostic method. More than half of the studies did not seem to include an assessment of general psychopathology, and only in 35% of studies was the ADHD diagnosis allocated by psychiatrists or psychologist. More than half of the studies included patients with psychiatric comorbidity.

**Conclusion:**

These findings raise concerns about the validity of the ADHD diagnosis in many of the included RCTs. It is worrying that securing a reasonably accurate diagnosis is not prioritized in more than half of the studies. If neither clinicians nor researchers can rely on the basic fact the patients in scientific studies diagnostically resemble the patients they are facing, scientific studies risk losing their clinical relevance. Since RCTs can lead to changes in clinical practice, they must be conducted carefully. To advance research on adult ADHD, the quality of the diagnostic assessment must be prioritized, requiring comprehensive differential diagnosis by a skilled psychiatrist or psychologist.

## Introduction

The number of adults receiving a diagnosis of Attention Deficit/Hyperactivity Disorder (ADHD) is increasing [1, 2]. In the World Federation of ADHD International Consensus Statement from 2021 it is estimated that ADHD occurs in 2.5% of adults [3]. However, a recent systematic review and meta-analysis found a global prevalence of symptomatic adult ADHD of 6.75% in 2020, corresponding to more than 366 million affected adults globally [4]. This number also includes individuals, who were diagnosed in childhood and who remained symptomatic in adulthood. However, several longitudinal studies have shown that most individuals with adult ADHD have not received a diagnosis of ADHD in childhood [4–6].

One factor that has been discussed as a contributing cause to the increasing number of adults receiving an ADHD diagnosis, in addition to increased clinical awareness, is the growing visibility of ADHD on social media platforms, where users are exposed to symptom descriptions and personal accounts that may prompt self-identification and help-seeking behavior [1, 7].

Originally, ADHD was described in children. The scientific origin of ADHD is by many considered to be the work of George F. Still at the turn of the 20th century, but clinical descriptions of what we today call ADHD can be found a century earlier in the works of Alexander Crichton [8]. In 1968, the diagnostic category of Hyperkinetic Reaction of Childhood (or Adolescence) was included in DSM-II, which described the disorder in terms of “overactivity, restlessness, distractibility, and short attention span, especially in young children; the behavior usually diminishes in adolescence” [9].

In the subsequent versions of the DSM [10–15], the diagnostic criteria for ADHD have been diluted and become more inclusive. Unlike most other adult mental disorders, which are defined by a combination of diagnostic criteria targeting behavioral and experiential anomalies, i.e., signs and symptoms, the diagnosis of ADHD is based on behavioral features (signs). Initially, this could hardly be any different, as the original diagnostic criteria specified observable behavioral features in children reported by adults (e.g., parents or teachers). Thus, the possibility of diagnosing ADHD in adults has involved what might be called a “subjectivization” of the diagnostic criteria of ADHD. Instead of basing a diagnostic assessment on reports of observed behavioral features (signs) from parents or teachers (e.g., of “excessive running or climbing (…) having difficulty sitting still” [10, p. 41] or of “interrupting, grabbing objects (…) excessive talking and by an inability to play quietly” [11, p. 50], the adult person must now herself consider if she believes she, e.g., has “difficulty sustaining attention in tasks,” is easily distracted by “unrelated thoughts,” “squirms in seat,” feels “restless,” etc. [14, p. 59f.]. She must also reflect upon whether she believes that some of these features were present in her childhood, making recall bias a crucial issue. This change in the perception of the diagnostic criteria from being ‘signs’ to being ‘symptoms’ may have lowered the diagnostic thresholds of ADHD.

This change in the perception of the criteria is also reflected in various national guidelines for diagnosing and managing ADHD in adults. Such guidelines often recommend applying diagnostic *interviews* for assessing ADHD. Yet, the national guidelines do not provide the level of evidence for interviews specifically aimed at diagnosing ADHD, e.g., European guidelines [16], UK guidelines [17, 18], and Australian guidelines [19]. A recent meta-analysis of self-report diagnostic methods for ADHD showed that they often yielded false-positive diagnoses [20]. Another systematic review found that methods of diagnosing ADHD in adulthood varied widely with respect to source of information, diagnostic instruments, diagnostic symptom threshold, and whether impairment was required for making the diagnosis. Here, sole reliance on self-reports was linked to a low diagnostic persistence estimate [21].

The above-described changes in or perception of the diagnostic criteria for ADHD may explain some of the global increase in prevalence. Moreover, the possibility of diagnosing ADHD in adults raises several issues, and scholars have stressed the need to examine the validity of the diagnostic category of ADHD in adults [22, 23]. Among the issues are difficulties in defining what “impaired functioning” is. Many adults endorse experiences that could perhaps sound like symptoms of ADHD [24] but if, say, experiences of inattention do not interfere with functioning, such experiences should not have the status of symptoms of ADHD according to DSM-5 [15].

In sum, the diagnostic criteria pose challenges for diagnosing ADHD in adults, since i) the original diagnostic criteria and tools for assessing ADHD were developed for use in children [25], ii) retrospective recall of childhood symptoms is notoriously poor [26], iii) the ADHD criteria were not tested in adults in the DSM-5 field trials, and iv) collateral information (e.g., from school teachers or parents), which previously was the foundation for the making the ADHD diagnosis, is difficult, if not impossible, to retrieve or access in adults [20]. Thus, we decided to examine how research studies have handled the challenges surrounding ADHD diagnosis.

## Aim

The aim of this study was to review how ADHD has been diagnosed in adults in Randomized Controlled Trials (RCTs).

## Methods

### Search strategy and selection criteria

Following the PRISMA guidelines [27], we conducted a review, focusing on the methods of diagnosing ADHD in adults in RCTs. We focused on RCTs as they rank very high in the hierarchy of evidence in evidence-based medicine and thus are likely to represent high-quality empirical research e.g., [28]. To be clear, we were only interested in the diagnostic methods and not the findings of these RCTs. We searched PubMed, using the following search string “ADHD OR Hyperkinetic Disorder AND Adult” on December 5, 2024. We restricted our search to humans and RCT, using PubMed filters. Inclusion criteria were RCT studies with adult samples (participants at least 18 years old) with a diagnosis of ADHD/Hyperkinetic disorder, studies written in English, and studies must include a direct patient assessment. Conference abstracts were excluded as well as studies relying on registry data. Authors IS and JN screened all titles and abstracts for inclusion in the study. Disagreement was resolved through consensus between the authors. We chose to only search one database (PubMed), because the aim was to get an overview of the methodology used to allocate the ADHD diagnosis in adults in RCTs.

### Data extraction

We extracted data on diagnostic methods, whether an assessment of general psychopathology was made, whether the study included patients with comorbid disorders in the sample, and on the person allocating the diagnosis (e.g., a medical doctor, psychologist, trained rater, or unknown).

### Categories

The diagnostic methods were categorized into five main groups based on how the ADHD diagnosis had been established: 1) studies that only used an ADHD-specific interview/rating scale; 2) Studies that only used clinical diagnoses, 3) studies that used a structured interview for assessing general psychopathology, 4) studies that used a semi-structured interview for assessing general psychopathology, and 5) studies that used other approaches. Some of these categories were further subdivided if there was an add-on to the main diagnostic approach, e.g., an ADHD-specific rating scale in addition to a structured interview for assessing general psychopathology.

The categorization process followed a systematic strategy:

1. Diagnostic tools: in each of the included studies, we identified the specific diagnostic instruments used (e.g., structured interviews, self-report scales, clinician-administered ADHD-specific interviews). If this information was not explicitly stated in the study itself, we traced it back to a *parent paper* (i.e., an original or referenced study) that provided details on the diagnostic method used. If studies did not describe assessing general psychopathology or report procedures that would allow such an assessment, we concluded that no such assessment had been made.
2. Differential diagnosis and hierarchical considerations: The presence of a systematic differential diagnostic process was determined based on the study’s inclusion and exclusion criteria or if it was explicitly described in the study, e.g., using a method allowing for differential diagnosis. We assessed whether studies adhered to a classical diagnostic hierarchy [29, 30], prioritizing organic disorders, followed by schizophrenia spectrum and bipolar disorders, and then other psychiatric conditions. If a study explicitly stated that such hierarchical exclusion criteria were applied, it was categorized accordingly.
3. Comorbidity: The handling of psychiatric comorbidities was assessed based on whether studies allowed participants with additional diagnoses (e.g., anxiety, depression) beyond ADHD.
4. Interviewer Qualifications: The qualifications of the individual conducting the diagnostic assessment were extracted from the article. We specifically looked for whether the study specified that a psychiatrist, psychologist, trained rater, or another professional was responsible for diagnosing participants. If this information was not available, we categorized it as “unknown.”

### Definitions

In this study, we defined a structured diagnostic interview as an interview consisting of a set of predetermined questions that should be presented in a definite order. Diagnostic information is yielded based on the patient’s responses to the questions and on the interviewer’s observations (an example of a structured interview for general psychopathology following this definition is the *Structured Clinical Interview for DSM* (SCID-I) [31]. Structured diagnostic interviews aim at identifying symptoms that meet diagnostic Criteria [32] and which can result in the allocation of a diagnosis. We defined a semi-structured diagnostic interview for general psychopathology as a conversational interview, aiming at eliciting psychopathological information but without using preformulated questions presented in a definite order. The interviewer’s questions function as triggers that encourage the patient to talk, and through his or her comments and questions, the interviewer steers the interview to obtain the relevant psychopathological data necessary for allocating a diagnosis [33].

## Results

**Figure 1.**
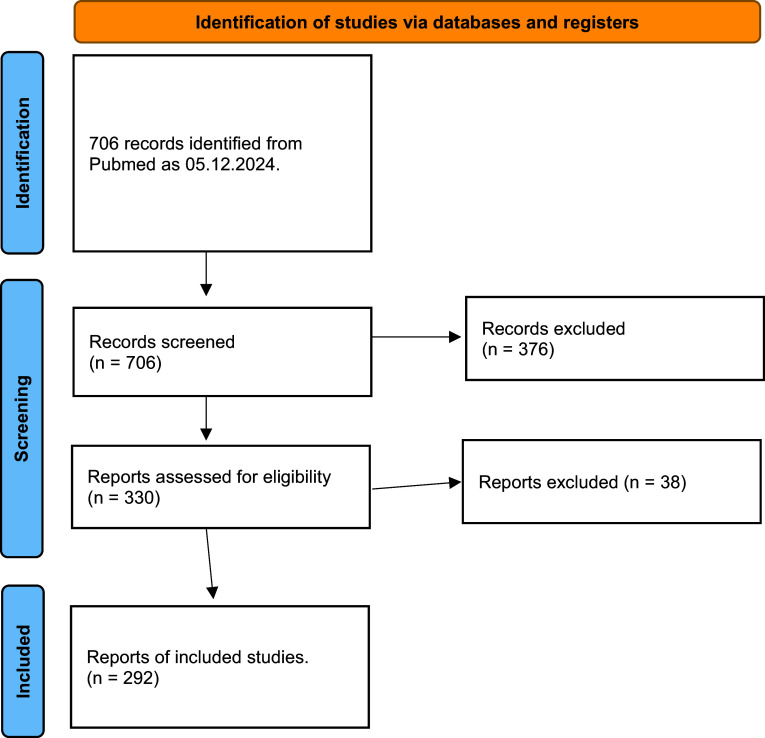
PRISMA flow diagram for inclusion of papers.

The PubMed search yielded 706 publications. 376 publications were excluded, leaving 330 which were assessed for eligibility. 38 were excluded for not meeting the inclusion criteria. We ultimately included 292 RCTs (see supplementary material for the list of the included studies). The study selection can be seen in Figure 1.

### Diagnostic methods

The diagnostic methods used to allocate ADHD diagnoses to adults in the included studies are shown in Table 1. Generally, the methods used to diagnose ADHD in adults varied considerably, and 49.7 % of the studies allocated the ADHD diagnosis without an assessment of general psychopathology. This group of studies is composed of studies using only clinical diagnoses, with (29.5%) or without (12.7%) an additional ADHD-specific rating scale and studies using only an ADHD-specific interview/rating scale (7.5%).

**Table 1.** Methods used for allocating ADHD diagnosis and the number of studies using the methods

	ADHD specific	Clinical diagnosis		Structured interview			Semi-structured interview		Other
Diagnostic methods	ADHD specific interview or rating scale (either self- or clinician rated)	Clinical diagnosis/ Paper state that patients met DSM or ICD criteria for ADHD	Clinical diagnosis/ Paper state that patients met DSM or ICD criteria for ADHD + ADHD specific rating scale	Structured interview for general psycho- pathology schedule (SCID or MINI)	Structured interview for general Psycho-pathology + ADHD specific ratingscale	Structured interview+ semi-structured + ADHD rating scale	Semi-structured interview for general psychopathology (Kiddie SADS)	Semi-structured interview for general psychopathology + ADHD specific ratingscale	Other
Number of papers using the methods (total *N* = 292)	22 (7.5%)	37 (12.7%)	86 (29.5%)	17 (5.8%)	91 (31.2%)	26 (8.9%)	4 (1.4%)	7 (2.4%)	2 (0.7%)

Among the studies that included an assessment of general psychopathology, the ADHD diagnosis was allocated either based on this assessment alone or in combination with a self- or clinician-rated scale targeting ADHD. When dividing studies that assessed general psychopathology into studies using structured vs. semi-structured interviews, the vast majority of studies used a structured diagnostic interview (see Table 1 for details).

### Who allocated the diagnosis?

In 190 studies (65%), the person who conducted the diagnostic assessment was either not reported, not a psychiatrist or a psychologist, or it was made by a computer (see table 2).

**Table 2.** Allocation of diagnosis and psychiatric comorbidity

Who did the diagnostic interview?	N (%)
Clinician	102 (35%)
Unknown	179 (61%)
Trained rater	10 (3%)
Other (computer allocated diagnosis, confirmed by a neurologist or psychiatrist)	1 (0.5%)
Allow comorbid diagnosis?	
Yes	157 (54%)
No	122 (42%)
Unknown	13 (4%)

### Comorbidity

Table 2 shows that from the total of 292 studies, 157 studies (53.8%) accepted some kind of psychiatric comorbidity in their sample. Moreover, 256 of the studies (87.7%) stated that they adhered to a diagnostic hierarchy, e.g., a diagnosis of an organic condition overrules an ADHD diagnosis. Simultaneously, most of these studies did not apply a method that included an assessment of whether the patients suffered from mental disorders, which they claimed would overrule the ADHD diagnosis.

## Discussion

This review examined how the ADHD diagnosis has been allocated in 292 empirical studies of adult patients. Overall, there was considerable variation in and no consensus about the method used for diagnosing ADHD. Moreover, the review identified three, interrelated methodological issues that raise concern about the quality of the allocated diagnoses in a substantial part of these studies.

First, half of the included studies did not describe conducting an examination of general psychopathology or report procedures that would have allowed for such an assessment, which is necessary for allocating any diagnosis. In these studies, either no diagnostic assessment was made (relying solely on clinical diagnoses), or the diagnosis was allocated based on results from a self- or clinician-rated scale targeting only ADHD, sometimes in combination with a clinical diagnosis. Just stating that a clinical diagnosis was used without any description of how and who made the clinical diagnosis is not sufficient as this can cover a wide range of diagnostic methods, diagnoses been made by untrained staff, different diagnostic traditions, errors, etc. [34, 35], and provides no transparency, which is of greatest importance in research [36]. However, without an assessment of general psychopathology, it is impossible to make a differential diagnosis, e.g., ruling out the possibility of other (often more severe) mental disorders that may present with similar signs or symptoms. Although 87.7% of the studies asserted that they adhered to a diagnostic hierarchy, this was practically impossible in most of these studies as they included no assessment of general psychopathology. Naturally, a general psychopathological assessment is crucial in the case of ADHD, because none of the diagnostic criteria of ADHD are specific to ADHD and similar signs and symptoms can be seen in a range of other mental disorders such as substance use disorder, schizophrenia spectrum disorders, mood disorders, etc. For example, attention deficits and motor disturbances have been described as parts of the psychopathology of schizophrenia since Bleuler coined the concept of schizophrenia in the early 20th century [37]. Also, disorders such as depression, anxiety, and trauma-related conditions can give rise to attentional complaints that mimic ADHD symptoms. These overlaps can lead to diagnostic confusion, particularly in adult populations, where developmental history may be less readily available or prone to recall bias. Mølstrøm et al. [38] highlight this issue in a study of first-admission psychiatric patients, demonstrating how affective and anxiety symptoms often manifest in non-specific complaints, including difficulties with concentration and attention. These findings underscore the importance of a thorough differential diagnostic process that takes into account the non-specific nature of attentional symptoms and the disease pictures they are embedded in. Thus, it is highly problematic that half of the included studies diagnosed ADHD apparently without assessing general psychopathology.

Although structured diagnostic interviews long have been regarded as a “gold standard” for diagnosing mental disorders in research, several studies have reported serious limitations with structured diagnostic interviews. For example, studies comparing the agreement of diagnoses allocated by a trained rater using structured interviews with best consensus diagnoses allocated by experienced psychiatrists using semi-structured diagnostic interviews and including all available information (e.g., from the clinic and relatives) have reported worryingly low overall concordances [39, 40]. The authors recommend that structured interviews should only be used in research with certain precautions, e.g., only by skilled medical doctors or psychologists and not by for-the-purpose trained raters. In our review, only 12.7% of the studies used a semi-structured interview to assess general psychopathology (1.4% used only a semi-structured interview, 2.4% used it in combination with a self- or clinician-rated scale for ADHD, and 8.9% used it in combination with a structured interview and an ADHD specific rating scale). The high reliance on structured interviews for assessing general psychopathology, amounting to a total of 45.9%, may have compromised the validity of the allocated ADHD diagnoses in these studies.

Second, only approximately one-third of the studies reported that the diagnosis had been allocated by a medical doctor or a psychologist. This is also a cause for concern because significant discrepancies repeatedly have been demonstrated for psychiatric diagnoses allocated by trained raters vs. clinicians [39, 40]. Moreover, self-rating measures to diagnose ADHD have a very low positive predictive value, often in the 10% range [20]. The reliance on specially trained raters and self-rating scales for diagnosing ADHD elevates the likelihood of diagnostic errors.

Third, more than half of the studies included participants who had some kind of psychiatric comorbidity. Although developmental disorders were removed as an exclusion criterion for the ADHD diagnosis in DSM-5 [20], other mental disorders still function as exclusion criteria for making the ADHD diagnosis—i.e., ADHD cannot be diagnosed if the symptoms occur only during the course of schizophrenia or another psychotic disorder or if the ADHD symptoms are better explained by other disorders such mood disorders, anxiety disorder, personality disorders, and substance use disorder, etc. [15]. The above-described omission of assessment of general psychopathology in half of the studies makes it impossible to know if the ADHD symptoms here occurred during the course of another disorder or if they were better explained by another disorder. In these studies, we cannot conclude that the ADHD diagnosis was made in accordance with the diagnostic guidelines. Of course, ADHD can, in some cases, be diagnosed as a comorbid condition [41].

The overall implication of these methodological issues is that the validity of the ADHD diagnoses in many of the included RCTs appears to be severely compromised. If these diagnoses were allocated on insufficient grounds, it has most likely affected the outcome of these trials, e.g., results of interventions in samples, whose diagnostic status was assumed to be ADHD but which in fact remain diagnostically unascertained. It also implies that comparing results across studies in reviews or meta-analyses comes with a high degree of uncertainty. Here, it may prove useful to exclude studies relying on insufficient diagnostic methods. For empirical studies researching subjects related to specific disorders, e.g., testing effects of treatment in ADHD, prioritization of careful allocation of diagnosis is of utmost importance.

With the sparse knowledge of how ADHD manifests in adults, and the need to rely on the patients’ own descriptions of their behavior as children to diagnose ADHD in adults, we are, diagnostically speaking, standing on unstable ground. The lack of real-time external observations of these patients, who are now adults, has transformed some of the behavioral *signs* of ADHD in children into *symptoms* of ADHD in adults, viz. the subjectivization of the diagnostic criteria. This change in the perception of some diagnostic criteria for a child vs adult ADHD raises the question as to whether ADHD diagnosed in childhood and ADHD diagnosed in adulthood is in fact the same disorder. Most patients, who are diagnosed with ADHD in adulthood, have not been diagnosed with ADHD in childhood [4–6]. Perhaps some of these adult ADHD patients were overlooked as children, but a more likely explanation seems to be that many of them did not attract psychiatric attention as children, because they did not show the same degree of behavioral manifestations as those children, who were diagnosed with ADHD in childhood. Again, there is an urgent need to clarify how exactly ADHD presents in adults and to establish diagnostic criteria to delineate the disorder from other conditions that also present with attention- and hyperkinetic phenomena.

Consequently, it seems premature to include patients with comorbid disorders in the empirical research studies of ADHD in adults, which nonetheless was the case in more than half of the studies. Due to the limited knowledge of ADHD disorder in adults, the aim must first be to comprehensively examine a sample of ADHD patients without comorbidities and follow them over time [42]. For now, we do not know if ADHD symptoms in adults are similar in patients with ADHD with or without psychiatric comorbidities. Psychopathological studies, clarifying the nature of the subjective experiences of being distracted by “unrelated thoughts” or “feeling restless” etc. in adult ADHD, may aid in differentiating such ADHD symptoms from seemingly similar symptoms in other mental disorders.

These diagnostic challenges underscore the importance of transparency and rigor when conducting empirical studies on ADHD, not the least RCTs, which are considered to be providing evidence of high quality [43]. Without clear and consistent reporting of diagnostic methods and procedures, the reliability of findings becomes questionable, potentially intensifying the difficulties already inherent in studying adult ADHD. As emphasized in Guidelines for Reporting Health Research: A User’s Manual [36]:

“Poorly conducted trials are a waste of time, effort, and money. The most dangerous risk associated with poor-quality reporting is an overestimation of the advantages of a given treatment … Whatever the outcome of a study, it is really hard for the average reader to interpret and verify the reliability of a poorly reported RCT. In turn, this problem could result in changes in clinical practice that are based on false evidence and that may harm patients.” [36, p. 3]. Transparent reporting is therefore essential, not only to ensure that RCTs provide reliable and interpretable evidence but also to safeguard clinical practice from being guided by potentially flawed “evidence.”

In conclusion the results of this review point to a worrying shift in the common understanding of how a psychiatric diagnosis should be allocated in research studies, with a dwindling awareness of the importance of making as accurate a diagnosis as possible, which necessarily implies making a comprehensive general psychopathological assessment. If we, both as clinicians and researchers, cannot be reasonably sure that patients in scientific studies actually suffer from the diagnosis which the study claims that they do, we cannot rely on the study’s findings.

Our finding that half of the RCTs exhibited little or no interest in securing the validity of the ADHD diagnosis and that it was unclear who made the diagnosis in 2/3 of the studies is certainly alarming. The diagnostic assessment is the foundation, which all subsequent analyses are built upon. As long as it remains unclear precisely what disorder is being examined in scientific studies, the findings of these studies will have limited value. In this context, it is noteworthy that we reviewed RCTs, and RCTs are considered high in the scientific evidence hierarchy in evidence-based medicine. Still, many RCTs had not made an effort to diagnose *lege artis*, thus rendering the results of their otherwise comprehensive study questionable.

## Supporting information

Studart et al. supplementary material 1 Studart et al. supplementary material

Studart et al. supplementary material 2 Studart et al. supplementary material

## Data Availability

Detailed information about dataextraction is available upon request

## Supplementary material

The supplementary material for this article can be found at http://doi.org/10.1192/j.eurpsy.2025.2447.
