# Supplementary material for: Diagnosing ADHD in adults in randomized controlled studies: a scoping review
Source: Eur Psychiatry. 2025 Apr 14;68(1):e64. doi: 10.1192/j.eurpsy.2025.2447 (PMC12188335; doi:10.1192/j.eurpsy.2025.2447)
Supplement: Studart et al. supplementary material 1 — Studart et al. supplementary material [file S0924933825024472sup001.docx]

Supplementary table. Key-points of the 292 included randomized controlled studies

| **Title** | **Author** | **Publication Year** | **Diagnostic 'tool' for allocating diagnosis** | **Differential diagnosis/Hierarchy of diagnoses** | **Comorbid Diagnosis Accepted** | **Interviewer** |
| --- | --- | --- | --- | --- | --- | --- |
| Long-term effectiveness and safety of dexmethylphenidate extended-release capsules in adult ADHD | Adler L. A., Spencer T., McGough J.J. Et al | 2009 | DSM-IV | Not mentioned | Yes | Unknown |
| Safety and tolerability of once versus twice daily atomoxetine in adults with ADHD | Adler L., Dietrich A., Reimherr F.W. Et al | 2006 | DSM-IV, Conners’ Adult ADHD Diagnostic Interview for DSM-IV (CAADID) | Yes (but no description of how) | No | Unknown |
| Functional outcomes in the treatment of adults with ADHD | Adler L.A. , Spencer T.J., Levine L.R. et al | 2008 | DSM-IV-TR, Conners Adult ADHD Diagnostic Interview for DSM-IV, SCID | Yes | No | Unknown |
| Pharmacokinetic and Pharmacodynamic Properties of Lisdexamfetamine in Adults with Attention-Deficit/Hyperactivity Disorder | Adler L.A., Alperin S., Leon T. et al | 2017 | Adult ADHD Clinician Diagnostic Scale | Yes | Yes | Unknown |
| Quality of life assessment in adult patients with attention-deficit/hyperactivity disorder treated with atomoxetine | Adler L.A., Sutton V.K. Moore R.J. Et al | 2006 | DSM-IV, SCID, Conners Adult ADHD Diagnostic Interview for DSM-IV | Yes | No | Unknown |
| Efficacy, Safety, and Tolerability of Centanafadine Sustained-Release Tablets in Adults With Attention-Deficit/Hyperactivity Disorder: Results of 2 Phase 3, Randomized, Double-blind, Multicenter, Placebo-Controlled Trials | Adler L.A., Adams J., Madera-McDonough J. et al | 2022 | ACDS | Unknown | Unknown | Unknown |
| Self-Reported quality of life in adults with attention-deficit/hyperactivity disorder and executive function impairment treated with lisdexamfetamine dimesylate: a randomized, double-blind, multicenter, placebo-controlled, parallel-group study | Adler L.A., Dirks B,, Deas P. et al | 2013 | DSM-IV-TR, ADHD-RS-IV | Yes | No | Unknown |
| Lisdexamfetamine dimesylate in adults with attention-deficit/ hyperactivity disorder who report clinically significant impairment in executive function: results from a randomized, double-blind, placebo-controlled study | Adler L.A., Dirks B., Deas P.F. et al | 2013 | DSM-IV, SCID, ADHD-RS | Yes | No | Clinician |
| Double-blind, placebo-controlled study of the efficacy and safety of lisdexamfetamine dimesylate in adults with attention-deficit/hyperactivity disorder | Adler L.A., Goodman D.W., Kollins S.H. et al | 2008 | DSm-IV, ADult ADHD Clinical Diagnostic Scale | Yes | Yes | Clinician |
| A Placebo-Controlled Trial of Lisdexamfetamine in the Treatment of Comorbid Sluggish Cognitive Tempo and Adult ADHD | Adler L.A., Leon T.L., Sardoff T.M. et al | 2021 | DSM 5, Adult ADHD Clinical Diagnostic Scale , MINI | Yes | No | Unknown |
| Atomoxetine treatment in adults with attention-deficit/hyperactivity disorder and comorbid social anxiety disorder | Adler L.A., Liebowitz M., Kronenberger W. et al | 2009 | DSM-IV, SCID, The Conners’ Adult ADHD Diagnostic Interview for DSM-IV | Yes | Yes | Unknown |
| Medication adherence and symptom reduction in adults treated with mixed amphetamine salts in a randomized crossover study | Adler L.A., Lynch L.R, Shaw D.M. et al | 2011 | DSM-IV-TR, SCID, Adult ADHD Clinician Diagnostic Scale (ACDS) version 1.2 | Yes | Yes | Unknown |
| Effectiveness and Duration of Effect of Open-Label Lisdexamfetamine Dimesylate in Adults With ADHD | Adler L.A., Lynch L.R., Shaw D.M. et al | 2013 | DSM-IV-TR , Adult ADHD Clinician Diagnostic Scale (ACDS) v1, SCID | Yes | No | Unknown |
| Executive Functioning Outcomes Over 6 Months of Atomoxetine for Adults With ADHD: Relationship to Maintenance of Response and Relapse Over the Subsequent 6 Months After Treatment | Adler L.A., Solanto M. , Escobar R. et al | 2020 | DSM-IV, Conners’ Adult ADHD Rating Scale–Investigator Rated: Screening Version | Yes (but no description of how) | Yes | Unknown |
| Short-term effects of lisdexamfetamine dimesylate on cardiovascular parameters in a 4-week clinical trial in adults with attention-deficit/hyperactivity disorder | Adler L.A., Weisler R.H., Goodman D.W. et al | 2009 | DSM-IV, Adult ADHD Clinical Diagnostic Scale | Yes | Yes | Psychiatrist |
| Efficacy and safety of OROS methylphenidate in adults with attention-deficit/hyperactivity disorder: a randomized, placebo-controlled, double-blind, parallel group, dose-escalation study | Adler L.A., Zimmerman B., Starr H.L. et al | 2009 | DSM-IV, Adult ADHD Clinical Diagnostic Scale version 1.2 28 | Yes | Unknown | Psychiatrist or MA degree |
| Non-specific effects of methylphenidate (Ritalin) on cognitive ability and decision-making of ADHD and healthy adults | Agay N. , Yechiam E., Carmel Z.et al | 2010 | DSM-IV, the Conners Adult ADHD Rating Scale, and the Wender–Utah Rating Scale. | Yes | No | Senior psychiatrist or a clinical expert |
| Cannabis Use in Adults Who Screen Positive for Attention Deficit/Hyperactivity Disorder: CANreduce 2.0 RCT Subgroup Analysis | Ahlers J., Baumgartner C., Augsburger M. et al | 2022 | ASRS | No | Yes | No interviewer, only self rated scale |
| Efficacy and safety of the novel α₄β₂ neuronal nicotinic receptor partial agonist ABT-089 in adults with attention-deficit/hyperactivity disorder: a randomized, double-blind, placebo-controlled crossover study | Apostol G., Abi-Saab W., Kratochvil C.J. et al | 2012 | DSM-IV, Adult ADHD Clinical Diagnostic Scale V 1.2 | Yes (but no description of how) | Yes | Unknown |
| Effects of mindfulness and psychoeducation on working memory in adult ADHD: A randomised, controlled fMRI study | Bachmann K., Lam A.P., Sörös P. et al | 2018 | DSM-IV | Yes | No | Unknown |
| Evidence for modulation of planning and working memory capacities by transcranial direct current stimulation in a sample of adults with attention deficit hyperactivity disorder | Barham H., Büyükgök D., Aksu S et al | 2022 | DSM-5, the Adult Self-Report Scale for ADHD (ASRS) and the Diagnostic Interview for ADHD in Adults 2.0 (DIVA 2.0) | Yes | No | Unknown |
| A randomized-controlled neurofeedback trial in adult attention-deficit/hyperactivity disorder | Barth B, Mayer-Carius K, Strehl U., Wyckoff S.N., Haeussinger F.B., Fallgatter A.J., Ehlis A.C. et al | 2021 | WURS-K, ADHD self-rating, SCID DSM IV scale, ADHS-SB, Wender Reimherr Interview, WR | Yes | No | Clinical Psychologist |
| The effects of lisdexamfetamine dimesylate on the driving performance of young adults with ADHD: a randomized, double-blind, placebo-controlled study using a validated driving simulator paradigm | Biederman J., Fried R., Hammerness P. et al | 2012 | DSM-IV, SCID, Kiddie Schedule forAffective Disorders and Schizophrenia- Epidemiologic Version (KSADS-E) | Yes | Yes | Psychiatrist |
| A randomized, 3-phase, 34-week, double-blind, long-term efficacy study of osmotic-release oral system-methylphenidate in adults with attention-deficit/hyperactivity disorder | Biederman J., Mick E., Surman C. et al | 2010 | DSM-IV, SCID, childhood disorders by modules from the Kiddie Schedule for Affective Disorders and Schizophrenia for School-Aged Children, Epidemiologic Version | Yes | Yes | Psychiatrist |
| A functional magnetic resonance imaging investigation of prefrontal cortex deep transcranial magnetic stimulation efficacy in adults with attention deficit/hyperactive disorder: A double blind, randomized clinical trial | Bleich-Cohen M., Gurevitch G., Carmi N. et al | 2021 | DSM-5, Adult ADHD Self-Report Scale (ASRS_ | Yes | Yes | Unknown |
| OROS-methylphenidate efficacy on specific executive functioning deficits in adults with ADHD: a randomized, placebo-controlled cross-over study | Bron T.I., Bijlenga D., Boonstra A.M. et al | 2014 | DSM-IV, SCID, DIVA | Yes | Yes | Psychologist |
| Open-label administration of lisdexamfetamine dimesylate improves executive function impairments and symptoms of attention-deficit/hyperactivity disorder in adults | Brown T.E., Brams M., Gao J. et al | 2010 | DSM-IV, Adult ADHD Clinical Diagnostic Scale version 1.2 (ACDS v1.2 | Yes | Yes | Unknown |
| Improved Executive Function in Adults Diagnosed With Attention-Deficit/ Hyperactivity Disorder as Measured by the Brown Attention-Deficit Disorder Scale Following Treatment With SHP465 Mixed Amphetamine Salts Extended-Release: Post Hoc Analyses From 2 Randomized, Placebo-Controlled Studies | Brown T.E., Chen J. and Robertson B. | 2022 | DSM-IV, ADHD-RS-IV | Yes | Yes | Unknown |
| Effect of atomoxetine on executive function impairments in adults with ADHD | Brown T.E., Holdnack J., Saylor K. et al | 2011 | DSM-IV, Adult ADHD Clinician Diagnostic Scale version 1.2 | Yes (but no description of how) | Yes | Unknown |
| Atomoxetine improved response inhibition in adults with attention deficit/hyperactivity disorder | Chamberlain S.R., Del Campo N., Jonathan Dowson J. et al | 2007 | DSM-IV | Yes | Yes | Psychiatrist |
| Efficacy of Cognitive Behavioral Therapy With and Without Medication for Adults With ADHD: A Randomized Clinical Trial | Cherkasova M.V., French L.R., Syer C.A. et al | 2020 | DSM-IV, Conners’ Adult ADHD Diagnostic Interview for DSM-IV | Yes | Yes | PhD in clinical psychology |
| Personalized Treatment of Mothers With ADHD and Their Young At-Risk Children: A SMART Pilot | Chronis-Tuscano A., Wang C.H., Strickland J. et al | 2016 | DSM-IV, ADHD Clinical Diagnostic Scale (ACDS) | No | Yes | Unknown |
| Divergence by ADHD subtype in smoking cessation response to OROS-methylphenidate | Covey L.S., Hu M.C, Weissman J.et al | 2011 | DSM-IV, adult ADHD Clinical Diagnostic Scale (ACDS) version 1.2 | Yes (but no description of how) | Yes | M.A degree. |
| An exploration of site effects in a multisite trial of OROS-methylphenidate for smokers with attention deficit/hyperactivity disorder | Covey L.S., Hu M.C., Green C.A. et al | 2011 | DSM-IV, Adult Clinical Diagnostic Scale version 1.2 | Yes (but no description of how) | Yes | Unknown |
| Long-acting methylphenidate reduces collision rates of young adult drivers with attention-deficit/hyperactivity disorder | Cox D.J., Davis M., Mikami A.Y., Singh H., Merkel R.L., Burket R. | 2012 | DSM-IV, SCID, Conners Adult ADHD Rating Scale, Barkley Structured Interview for ADHD | Yes | No | 'Trained examiner'' |
| Parent-Reported Improvements in Family Functioning in a RCT of Lisdexamfetamine for Treatment of Parental Attention-Deficit/Hyperactivity Disorder | Dara E Babinski, James G Waxmonsky, Daniel A Waschbusch et al | 2017 | DSM-IV or DSM-5 | Yes | No | Clinician with an MD/PhD |
| Transcranial Direct Current Stimulation to the Left Dorsolateral Prefrontal Cortex Improves Cognitive Control in Patients With Attention-Deficit/Hyperactivity Disorder: A Randomized Behavioral and Neurophysiological Study | Dubreuil-Vall L., Gomez-Bernal F., Villegas A.C. et al | 2021 | DSM-5 , MINI, ASRS | Yes | Yes | Unknown |
| An RCT of a CBT Intervention for Emerging Adults with ADHD Attending College: Functional Outcomes | Eddy L.D., Anastopoulos A.D. and Dvorsky M.R. | 2021 | DSM-5, Semi-Structured Interview for Adult ADHD, ADHD Rating Scale-5, SCID | Yes | Yes | Clinical Psychologist |
| Cognitive behaviour therapy in medication-treated adults with ADHD and persistent symptoms: a RCT | Emilsson B., Gudjonsson G., Sigurdsson J.F. et al | 2011 | DSM-IV, Kiddie-Schedule for Affective Disorders and Schizophrenia (K-SADS-PL) ADHD section, present and lifetime version, Barkley ADHD Current Symptoms Scale | Yes | Yes | 'Mental health professionals'' |
| Neural correlates of atomoxetine improving inhibitory control and visual processing in Drug-naïve adults with attention-deficit/hyperactivity disorder | Fan L.Y., Chou T.L. And Shur-Fen Gau S. | 2017 | DSM-IV, Conners’ Adult ADHD Diagnostic Interview, modified adult version of the ADHD supplement of the Chinese version of the Schedule for Affective Disorders and Schizophrenia–Epidemiological Version (K-SADS-E | Yes | No | Psychiatrist |
| Triple-Bead Mixed Amphetamine Salts (SHP465) in Adults With ADHD: Results of a Phase 3, Double-Blind, Randomized, Forced-Dose Trial | Frick G., Brian Yan B. and A Adler L.A. | 2020 | DSM-IV, SCID, Adult ADHD Clinical Diagnostic Scale Version 1.2, ADHD-RS-IV | Yes | Yes | Unknown |
| Personality Disorder in Adult Attention-Deficit/Hyperactivity Disorder: Attrition and Change During Long-term Treatment | Gift T.E., Reimherr F.W., Marchant B.K. et al | 2016 | DSM-IV, Utah Criteria of ADHD, SCID, Wender Utah Rating Scale | Yes | Yes | Unknown |
| Randomized, 6-Week, Placebo-Controlled Study of Treatment for Adult Attention-Deficit/Hyperactivity Disorder: Individualized Dosing of Osmotic-Release Oral System (OROS) Methylphenidate With a Goal of Symptom Remission | Goodman D.W., Starr H.L., Ma Y.W. et al | 2017 | DSM-IV, ADHD Clinical Diagnostic Scale (ACDS), MINI | Unknown | Yes | Unknown |
| Effectiveness of a Tailored Intervention for Women With Attention Deficit Hyperactivity Disorder (ADHD) and ADHD Symptoms: A Randomized Controlled Study | Gutman S.A., Balasubramanian S. And Herzog M. | 2020 | Self Report | Yes | Yes | Unknown/none |
| Reduced ADHD symptoms in adults with ADHD after structured skills training group: results from a RCT | Hirvikoski T., Waaler E., Alfredsson J. et al | 2011 | DSM-IV, Wender Utah Rating Scale (WURS) | Yes | Yes | Psychiatrist and Psychologist |
| Cognitive-Behavioral Therapy for Adult ADHD: A Randomized Clinical Trial in China | Huang F., Tang Y.L, Zhao M. et al | 2019 | DSM-IV, SCID, ADHD-RS | Yes | No | Unknown |
| Open-label dose optimization of methylphenidate modified release long acting (MPH-LA): a post hoc analysis of real-life titration from a 40-week randomized trial | Huss M., Ginsberg Y., Arngrim T. et al | 2014 | DSM-IV, DSM-IV ADHD Rating Scale | Unknown | No | Unknown |
| Efficacy and Safety of Guanfacine Extended-Release in the Treatment of Attention-Deficit/Hyperactivity Disorder in Adults: Results of a Randomized, Double-Blind, Placebo-Controlled Study | Iwanami A., Saito K., Fujiwara M. et al | 2020 | DSM-5 | Yes | No | Unknown |
| A randomized, double-blind, placebo-controlled crossover study of α4β 2* nicotinic acetylcholine receptor agonist AZD1446 (TC-6683) in adults with attention-deficit/hyperactivity disorder | Jucaite A., Öhd J., Potter A.S. et al | 2014 | DSM-IV, Conner's adult ADHD rating scale investigator administered | Yes | Yes | Psychiatrist |
| Reinforcing and subjective effects of methylphenidate in adults with and without attention deficit hyperactivity disorder (ADHD) | Kollins S.H,, English J., Robinson R. et al | 2009 | DSM-IV, SCID, Conners’ Adult ADHD Interview for DSM-IV | Yes | No | Trained clinician |
| A pilot study of lis-dexamfetamine dimesylate (LDX/SPD489) to facilitate smoking cessation in nicotine-dependent adults with ADHD | Kollins S.H., English J.S., Itchon-Ramos N. et al | 2014 | Conners’ Adult ADHD Interview, DSM-IV, Conners’ Adult ADHD Rating Scale, SCID | Yes | Yes | Unknown |
| Methylphenidate for attention deficit hyperactivity disorder and drug relapse in criminal offenders with substance dependence: a 24-week randomized placebo-controlled trial | Konstenius M., Jayaram-Lindström N., Guterstam J. et al | 2013 | DSM-IV, Adult ADHD Self-Rating Scale, the Wender Utah Rating Scale, the Structured Clinical Interview for DSM-IV I and II (SCID I and II), the Addiction Severity Index (ASI), Conners’ continuous performance test | Yes | Yes | Psychiatrists and psychologists |
| Response to methylphenidate in adults with ADHD is associated with a polymorphism in SLC6A3 (DAT1) | Kooij J.S., Boonstra A.M., Vermeulen S.H. et al | 2008 | DSM-IV, ADHD-rating scale | Yes | Yes | Psychiatrist |
| Once-daily atomoxetine for adult attention-deficit/hyperactivity disorder: a 6-month, double-blind trial | Lenard A Adler L.A., Thomas Spencer T., Brown T.E. et al | 2009 | DSM-IV, SCID, the Adult ADHD Clinician Diagnostic Scale version 1.2 | Yes | No | Unknown |
| Extended-Release Mixed Amphetamine Salts vs Placebo for Comorbid Adult Attention-Deficit/Hyperactivity Disorder and Cocaine Use Disorder: A Randomized Clinical Trial | Levin F.R., Mariani J.J., Specker S. et al | 2015 | DSM-IV-TR | Yes | Yes | Unknown |
| How treatment improvement in ADHD and cocaine dependence are related to one another: A secondary analysis | Levin F.R., Choi C.J., Pavlicova M. et al | 2018 | Conners Adult ADHD Diagnostic Interview for Diagnostic and Statistical Manual of Mental Disorders (DSM-IV), SCID DSM-5 | Yes | Yes | Unknown |
| Discrete Global but No Focal Gray Matter Volume Reductions in Unmedicated Adult Patients With Attention-Deficit/Hyperactivity Disorder | Maier S., Perlov E., Graf E. et al | 2016 | DSM-IV, ADHS-DC, Wender-Utah Rating Scale, SCID | Yes | Yes | Experienced postgraduate-level assessors |
| Efficacy of metadoxine extended release in patients with predominantly inattentive subtype attention-deficit/hyperactivity disorder | Manor I, Newcorn J.H., Faraone S.V. et al | 2013 | DSM-IV, Adult ADHD Clinical Diagnostic Scale version 1.2 (ACDS v1.2) the Structured Clinical Interview for DSM-IV (SCID) | Yes | Unknown | Unknown |
| Methylphenidate transdermal system in adult ADHD and impact on emotional and oppositional symptoms | Marchant B.K., Reimherr F.W., Robison R.J. et al | 2011 | DSM-IV, the Utah Criteria for ADHD | Yes | Yes | Unknown |
| Differentiating depression and ADHD without depression in adults with processing-speed measures | Martiny K., Nielsen N.P. And Wiig E.H. | 2020 | Brown Attenttion Deficit Disorder, ASRS | Yes | No | Psychiatrist |
| Responsiveness of the Adult Attention-Deficit/Hyperactivity Disorder Quality of Life Scale (AAQoL) | Matza L.S. , Johnston J.A., Faries D.E. et al | 2007 | Conners’ Adult ADHD Diagnostic Interview for DSM-IV (CAADID) | No | Unknown | Unknown |
| Supplementary guanfacine hydrochloride as a treatment of attention deficit hyperactivity disorder in adults: A double blind, placebo-controlled study | Max E Butterfield M.E., Saal J., Young B. et al | 2016 | DSM-IV | Yes | Yes | Psychiatrist |
| Effects of smoking abstinence on adult smokers with and without attention deficit hyperactivity disorder: results of a preliminary study | McClernon F.J. , Kollins S.H., Lutz A.M. et al | 2008 | DSM-IV, SCID, Conners’ Adult ADHD Interview, elf-report and observer versions of the Conners’ Adult ADHD Rating Scale | Yes | Unknown | Unknown |
| The effect of memantine in adult patients with attention deficit hyperactivity disorder | Mohammadzadeh S., Ahangari K. And Yousefi F. | 2019 | DSM-IV, SCID, Kiddie | Yes | No | Psychiatrist |
| Safety, tolerability and pharmacokinetics of 2-pyridylacetic acid, a major metabolite of betahistine, in a phase 1 dose escalation study in subjects with ADHD | Moorthy G., Sallee F., Gabbita P., Zemlan F., Sallans L., Desai P.B. | 2015 | DSM-IV, Conners Adult ADHD Rating Scale Self-Report (CAARS) | No | Yes | Unknown |
| Increased default-mode variability is related to reduced task-performance and is evident in adults with ADHD | Mowinckel A.M., Alnæs D., Pedersen M.L.et al | 2017 | DSM-IV-TR, MINI, DIVA 2.0 | Yes | No | Unknown |
| Efficacy of Group Cognitive Behavior Therapy Targeting Time Management for Adults with Attention Deficit/Hyperactivity Disorder in Japan: A Randomized Control Pilot Trial | Nakashima M., Inada N., Tanigawa Y. et al | 2021 | DSM-IV, SCID | Yes | Yes | Unknown |
| Efficacy and safety of guanfacine extended-release in Japanese adults with attention-deficit/hyperactivity disorder: Exploratory post hoc subgroup analyses of a randomized, double-blind, placebo-controlled study | Naya N., Sakai C., Okutsu D. et al | 2021 | DSM-5 | Yes | Yes | Unknown |
| A head-to-head randomized clinical trial of methylphenidate and atomoxetine treatment for executive function in adults with attention-deficit hyperactivity disorder | Ni H.C., Shang C.Y., Shur-Fen Gau S. et al | 2013 | DSM-IV , Adult ADHD Self-Report Scale-v1.1, Schedule for Affective Disorders and Schizophrenia–Epidemiological Versio | Yes | Yes | Child Psychiatrist |
| A comparison of efficacy between cognitive behavioral therapy (CBT) and CBT combined with medication in adults with attention-deficit/hyperactivity disorder (ADHD) | Pan M.R., Huang F., Zhao M.J. et al | 2019 | DSM-IV, Conners ’ Adult ADHD Diagnostic Interview for DSM-IV | Yes | Yes | Unknown |
| Efficacy of cognitive behavioural therapy in medicated adults with attention-deficit/hyperactivity disorder in multiple dimensions: a randomised controlled trial | Pan M.R., Zhang S.Y., Qiu S.W. et al | 2022 | Conners’ Adult ADHD Diagnostic Interview, DSM-IV, SCID | Yes | Yes | Psychiatrist |
| Cognitive behavioural therapy in groups for medicated adults with attention deficit hyperactivity disorder: protocol for a randomised controlled trial | Pan M.R., Zhao M.-J., Lie L. et al | 2020 | Conners’ Adult ADHD Diagnostic Interview, DSM IV | Yes | Yes | Unknown |
| A randomized controlled multicenter trial on the multimodal treatment of adult attention-deficit hyperactivity disorder: enrollment and characteristics of the study sample | Philipsen A., Graf E., Jans T. et al | 2014 | SCID, Conners Adult ADHD Rating Scales | Yes | Yes | "Experienced postgraduate-level assessors" |
| Acute nicotine improves cognitive deficits in young adults with attention-deficit/hyperactivity disorder | Potter A, NewhouseP | 2008 | DSM-IV, SCID, Wender–Utah Rating scale, the K-SADS-PL structured interview behavior disorder supplement | Yes | No | Unknown |
| Effects of acute ultra-low dose mecamylamine on cognition in adult attention-deficit/hyperactivity disorder (ADHD) | Potter A.S., Ryan K.K. and Newhouse P.A. | 2009 | DSM-IV, SCID, K-SADS-PL structured interview behavior disorder supplement, The Wender–Utah rating scale | Yes | No | Unknown |
| A double-blind, placebo-controlled, crossover study of osmotic release oral system methylphenidate in adults with ADHD with assessment of oppositional and emotional dimensions of the disorder | Reimherr F.W., Williams E.D., Strong R.E. et al | 2007 | DSM-IV, Conners Diagnostic Interview | Yes | No | Unknown |
| Efficacy of reboxetine in adults with attention-deficit/hyperactivity disorder: a randomized, placebo-controlled clinical trial | Riahi F., Tehrani-Doost M. Shahrivar Z.et al | 2010 | DSM-IV, SCID, Wender Utah Rating Scale (WURS), the ADHD section of the kiddie schedule for affective disorders and schizophrenia-present and lifetime version (K-SADS-PL) | Yes | No | Unknown |
| A randomized, double-blind, crossover comparison of MK-0929 and placebo in the treatment of adults with ADHD | Rivkin A., Alexander R.C., Knighton J. et al | 2012 | DSM-IV | No | Unknown | Unknown |
| A randomised, placebo-controlled, 24-week, study of low-dose extended-release methylphenidate in adults with attention-deficit/hyperactivity disorder | Rösler M., Fischer R, Ammer R. et al | 2009 | DSM-IV, SCID, the Wender Utah rating scale (WURS) | No | Yes | Psychiatrist |
| Twenty-four-week treatment with extended release methylphenidate improves emotional symptoms in adult ADHD | Rösler M., Retz W., Fischer R. et al | 2010 | DSM-IV, the Wender Utah Rating scale (WURS, Wender 1995) | Yes | Yes | Psychiatrist |
| Medication adherence in psychopharmacologically treated adults with ADHD | Safren S.A., Duran P., Yovel I. et al | 2007 | DSM-IV, SCID, the ADHD section of Kiddie SADS-E | Unknown | Unknown | Unknown |
| The efficacy of cognitive-behavioral therapy for older adults with ADHD: a RCT | Solanto M.V., Surman C.B., Ma J. et al | 2018 | DSM-IV, SCID, Conners Adult ADHD Diagnostic Interview | Yes | Yes | Unknown |
| Efficacy of meta-cognitive therapy for adult ADHD | Solanto M.V.,Marks D.J., Wasserstein J. et al | 2010 | DSM-IV, SCID, Conners Adult ADHD Diagnostic Interview for DSM-IV (CAADID) | Yes | Yes | Unknown |
| Hyperactivity/restlessness is associated with increased functional connectivity in adults with ADHD: a dimensional analysis of resting state fMRI | Sörös P., Hoxhaj E., Borel P. et al | 2019 | Structured Clinical Interview (SCID) for DSM-IV, Wender Utah Rating Scale, Conners Adult ADHD Rating Scales-Self Report | Yes | Yes | Psychiatrists |
| Does L-Methylfolate Supplement Methylphenidate Pharmacotherapy in Attention-Deficit/Hyperactivity Disorder?: Evidence of Lack of Benefit From a Double-Blind, Placebo-Controlled, Randomized Clinical Trial | Surman C., Ceranoglu A., Vaudreuil C., Albright B. et al | 2019 | DSM-IV | Yes | Yes | Psychiatrists |
| Post-Hoc Analyses of the Effects of Baseline Sleep Quality on SHP465 Mixed Amphetamine Salts Extended-Release Treatment Response in Adults with Attention-Deficit/Hyperactivity Disorder | Surman C.B.H., Robertson B., Chen J. et al | 2019 | DSM-IV-TR | Yes | No | Unknown |
| A Randomized Controlled Study of a Cognitive Behavioral Planning Intervention for College Students With ADHD: An Effectiveness Study in Student Counseling Services in Flanders | Van der Oord S., Boyer B.E., Van Dyck L. et al | 2020 | DSM-IV, SCID, MINI, DIVA | Yes | No | Unknown |
| Methylphenidate significantly improves driving performance of adults with attention-deficit hyperactivity disorder: a randomized crossover trial | Verster J.C., Bekker E.M., de Roos M. et al | 2008 | DSM-IV, DSM-IV ADHD-rating scale | Yes | No | Experienced psychiatrists |
| Methylphenidate significantly improves declarative memory functioning of adults with ADHD | Verster J.C., Bekker E.M., Kooij J.J.et al | 2010 | DSM-IV, DSM-IV ADHD-rating scale, Conners Adult ADHD rating scale | Yes | No | Experienced psychiatrists |
| Atomoxetine in comorbid ADHD/PTSD: A randomized, placebo controlled, pilot, and feasibility study | Wang Z., Zuschlag Z.D., Myers U.S. et al | 2022 | Conners’ ADHD Rating Scale | Yes | Yes | Unknown |
| Treatment With Lisdexamfetamine Dimesylate Improves Self- and Informant-Rated Executive Function Behaviors and Clinician- and Informant-Rated ADHD Symptoms in Adults: Data From a Randomized, Double-Blind, Placebo-Controlled Study | Weisler R., Ginsberg L, Dirks B. et al | 2014 | Adult ADHD Clinical Diagnostic Scale, ADHD-RS-IV with adult prompts, | Yes | No | Unknown |
| A randomized double-blind trial of paroxetine and/or dextroamphetamine and problem-focused therapy for attention-deficit/hyperactivity disorder in adults | Weiss M., Hechtman L. and Adult ADHD Research Group | 2006 | DSM-IV | Yes | Yes | Unknown |
| Efficacy and Safety of PRC-063, Extended-Release Multilayer Methylphenidate in Adults with ADHD Including 6-Month Open-Label Extension | Weiss M.D., Childress A.C. And Donnelly G.A.E. | 2021 | DSM-5, SCID, Conners’ Adult ADHD Diagnostic Interview for DSM-IV | Yes | No | Unknown |
| A one year trial of methylphenidate in the treatment of ADHD | Wender P.H., Reimherr F.W., Marchant B.K. et al | 2011 | Utah Criteria for ADHD, Wender-Reimherr Adult Attention Deficit Disorder Scale (WRAADDS | Yes | No | Board certified psychiatrists |
| A randomized, double-blind study of SHP465 mixed amphetamine salts extended-release in adults with ADHD using a simulated adult workplace design | Wigal T., Brams M., Frick G. et al | 2018 | DSM-IV-TR | Yes | No | Psychiatrist |
| Effect size of lisdexamfetamine dimesylate in adults with attention-deficit/hyperactivity disorder | Wigal T., Brams M., Gasior M. et al | 2011 | DSM-IV, ADHD-RS-IV | Yes (but no description of how) | Yes | Unknown |
| Randomized, double-blind, placebo-controlled, crossover study of the efficacy and safety of lisdexamfetamine dimesylate in adults with attention-deficit/hyperactivity disorder: novel findings using a simulated adult workplace environment design | Wigal T., Brams M., Gasior M., et al | 2010 | DSM-IV-TR, Adult ADHD Clinical Diagnostic Scale version 1.2 | Yes | Yes | Unknown |
| Effects of SHP465 mixed amphetamine salts in adults with ADHD in a simulated adult workplace environment | Wigal T., Childress A., Frick G. et al | 2018 | DSM-IV-TR, SCID | Yes | Yes | Unknown |
| A Double-Blind, Placebo-Controlled, Phase II Study to Determine the Efficacy, Safety, Tolerability and Pharmacokinetics of a Controlled Release (CR) Formulation of Mazindol in Adults with DSM-5 Attention-Deficit/Hyperactivity Disorder (ADHD) | Wigal T.L., Newcorn J.H. And Handal N | 2018 | DSM-5, MINI | Yes | Yes | 'Licensed Clinicians'' |
| Atomoxetine treatment of adults with ADHD and comorbid alcohol use disorders | Wilens T.E., Adler L.A., Weiss M.D. et al | 2008 | DSM-IV, SCID,A dult ADHD Clinician Diagnostic Scale | Yes | Yes | Unknown |
| Correlates of alcohol use in adults with ADHD and comorbid alcohol use disorders: exploratory analysis of a placebo-controlled trial of atomoxetine | Wilens T.E., Adler L.A., Tanaka Y. et al | 2011 | DSM-IV-TR, Adult ADHD Clinical Diagnostic Scale (ACDS) version 1.2, | Yes | No | Unknown |
| Impact of attention-deficit/hyperactivity disorder (ADHD) treatment on smoking cessation intervention in ADHD smokers: a randomized, double-blind, placebo-controlled trial | Winhusen T.M., Somoza E.C., Brigham G.S. et al | 2010 | DSM-IV, Adult Clinical Diagnostic Scale version 1.2 | Yes | No | Unknown |
| Once-daily treatment with atomoxetine in adults with attention-deficit/hyperactivity disorder: a 24-week, randomized, double-blind, placebo-controlled trial | Young J.L., Sarkis E., Qiao M. et al | 2011 | DSM-IV, SCID, Conners’ Adult ADHD Diagnostic Interview for DSM-IV | Yes | No | Psychiatrist or Psychologist |
| Neurofeedback, sham neurofeedback, and cognitive-behavioural group therapy in adults with attention-deficit hyperactivity disorder: a triple-blind, randomised, controlled trial | Schönenberg M. , Wiedemann E., Schneidt A. et al | 2017 | DSM-IV, MINI, WURS, ADHD-SB, Wender-Reimherr interview | Yes | Yes | 'Experienced clinical assessors'' |
| Reward modulation of cognitive function in adult attention-deficit/hyperactivity disorder: a pilot study on the role of striatal dopamine | Aarts E.,van Holstein M., Hoogman M. et al | 2015 | DSM-IV-TR, DIVA (Diagnostic Interview for Adult ADHD), SCID | Yes | Yes | Psychiatrists or psychologist |
| Attention-deficit/hyperactivity disorder and the explore/exploit trade-off | Addicott M.A., Pearson J.M., Schechter J.C. et al | 2021 | Conners’ Adult ADHD Diagnostic Interview, DSM-IV, MINI | Yes | Yes | Unknown |
| Executive function in adults with attention-deficit/hyperactivity disorder during treatment with atomoxetine in a randomized, placebo-controlled, withdrawal study | Adler L., Tanaka Y. Williams D. et al | 2014 | DSM-IV, Conners’ Adult ADHD Diagnostic Interview for DSM-IV, | YEs | No | Unknown |
| Atomoxetine effects on executive function as measured by the BRIEF--a in young adults with ADHD: a randomized, double-blind, placebo-controlled study | Adler L.A. , Clemow D.B., Williams D.W. er al | 2014 | DSM-IV-TR, Adult ADHD Clinical Diagnostic Scale (ACDS) | Unknown | Yes | Unknown |
| The reliability and validity of self- and investigator ratings of ADHD in adults | Adler L.A., Faraone S.V., Spencer T.J. et al. | 2008 | DSM-IV, SCID, Conners Adult ADHD Rating Scales | Yes | No | Unknown |
| Long-term, open-label study of the safety and efficacy of atomoxetine in adults with attention-deficit/hyperactivity disorder: an interim analysis | Adler L.A., Spencer T.J., Milton D. R. et al | 2005 | DSM-IV, Clinical Interview, SCID, Conners’ Adult ADHD Diagnostic Interview for DSM-IV (CAAR-D; Conners et al 1999) | Yes | No | Unknown |
| A Long-Term, Open-Label, Safety Study of Triple-Bead Mixed Amphetamine Salts (SHP465) in Adults With ADHD | Adler L.A., Frick G. andYan B. | 2020 | DSM-IV, SCID, Adult ADHD Clinical Diagnostic Scale Version 1.2, ADHD-RS-IV | Yes | Yes | Unknown |
| Long-term safety of OROS methylphenidate in adults with attention-deficit/hyperactivity disorder: an open-label, dose-titration, 1-year study | Adler L.A., Orman C., Starr H.L. et al | 2011 | DSM-IV, Adult ADHD Clinical Diagnostic Scale version 1.2. | Yes (but no description of how) | No | Unknown |
| Concurrent cannabis use during treatment for comorbid ADHD and cocaine dependence: effects on outcome | Aharonovich E., Fatima Garawi F. Bisaga A. et al | 2006 | DSm-IV, SCID | Yes | Yes | Unknown |
| Alleviation of ADHD symptoms by non-invasive right prefrontal stimulation is correlated with EEG activity | Alyagon U., Shahar H., Hadar A. et al | 2020 | DSM-5 | Yes | No | Senior Psychiatrist |
| Double-blind controlled trial of venlafaxine for treatment of adults with attention deficit/hyperactivity disorder | Amiri S., Farhang S., Ghoreishizadeh M.A. et al | 2012 | Kiddie Schedule for Affective Disorders and Schizophrenia for School-Aged Children, Present and Lifetime Versions (K-SADS-PL), Conners Adult ADHD Rating Scale, SCID, DSM IV | Yes | No | Psychiatrist |
| Efficacy of a synbiotic in the management of adults with Attention-Deficit and Hyperactivity Disorder and/or Borderline Personality Disorder and high levels of irritability: Results from a multicenter, randomized, placebo-controlled, "basket" trial. | Arteaga-Henríquez G., Ramos-Sayalero C., Ibañez-Jimenez P. | 2024 | DSM-5, DIVA, SCID, MINI | Yes | Yes | Unknown |
| Treating parents with attention-deficit/hyperactivity disorder: the effects of behavioral parent training and acute stimulant medication treatment on parent-child interactions | Babinski D.E., Waxmonsky J.G.,  Pelham W.E. Jr | 2014 | ADHD Rating Scale (ADHD-RS), SCID | Yes | No | Unknown |
| A randomized pilot study of the efficacy and safety of ABT-089, a novel α4β2 neuronal nicotinic receptor agonist, in adults with attention-deficit/hyperactivity disorder | Bain E.E. , Apostol G., Sangal R.B. et al | 2012 | DSM-IV, Adult ADHD Clinical Diagnostic Scale | Yes | No | Unknown |
| A pilot study of the effects of atomoxetine on driving performance in adults with ADHD | Barkley R.A.,  Anderson D.L., Kruesi M. Et al | 2007 | DSM-IV, ''structured interview'' (Barkley, Murphy, DuPaul, & Bush, 2002), SCID (parts of) | Yes | Yes | Licensed clinical psychologist |
| Effects of two doses of methylphenidate on simulator driving performance in adults with attention deficit hyperactivity disorder | Barkley R.A., Murphy K.R., O'Connell T. Et al | 2005 | DSM-IV, SCID | Yes | Yes | Psychologist |
| Characterizing the Placebo Response in Adults With ADHD | Ben-Sheetrit J., Peskin M., Newcorn J.H. et al | 2020 | DSM-IV, Adult ADHD Clinical Diagnostic Scale | Yes | No | Unknown |
| Attention-deficit/hyperactivity disorder (ADHD) symptoms, craving to smoke, and tobacco withdrawal symptoms in adult smokers with ADHD | Berlin I., Hu M.C., Covey L.S. et al | 2012 | DSM-IV, Adult Clinical Diagnostic Scale version 1.2 | No | Unknown | Unknown |
| Long-term safety and effectiveness of mixed amphetamine salts extended release in adults with ADHD | Biederman J,, Spencer T.J. Wilens T.E. Et al | 2005 | DSM-IV-TR | Yes (but no description of how) | No | Unknown |
| A double-blind comparison of galantamine hydrogen bromide and placebo in adults with attention-deficit/hyperactivity disorder: a pilot study | Biederman J., Mick E., Faraone S. Et al | 2006 | DSM-IV, SCID, modules (DSM-IV ADHD and conduct disorder) from the Kiddie Schedule for Affective Disorders and Schizophrenia (Epidemiologic Version) | Yes | Yes | Psychiatrist |
| Memantine in the Treatment of Executive Function Deficits in Adults With ADHD | Biederman J., Fried R., Tarko L. et al | 2017 | DSM-IV, Adult ADHD Investigator Symptom Report Scale (AISRS), BRIEF-A version | Yes | Yes | Unknown |
| A randomized, placebo-controlled trial of OROS methylphenidate in adults with attention-deficit/hyperactivity disorder | Biederman J., Mick E, Surman C. Et al | 2006 | DSM-IV, SCID, hildhood disorders by modules (DSM-IV ADHD and conduct disorder) from the SADS for School-Age Children–Epidemiologic Version | Yes | Yes | Psychiatrist |
| Are stimulants effective in the treatment of executive function deficits? Results from a randomized double blind study of OROS-methylphenidate in adults with ADHD | Biederman J., Mick E., Fried R. et al | 2011 | DSM-IV, SCID, Kiddie SADS-E | Yes | Yes | Psychiatrist |
| Is response to OROS-methylphenidate treatment moderated by treatment with antidepressants or psychiatric comorbidity? A secondary analysis from a large randomized double blind study of adults with ADHD | Biederman J., Mick E., Spencer T: et al | 2012 | DSM-IV, AISRS | Yes | Yes | Unknown |
| Positive effects of repetitive transcranial magnetic stimulation on attention in ADHD Subjects: a randomized controlled pilot study | Bloch Y., Harel E.V., Aviram S. Et al | 2010 | DSM-IV, Adult ADHD Self Report Scale (ASRS) and the Wender-Utah adult ADHD scale (WUAAS). | Yes (but no description of how) | No | Psychiatrist |
| Hyperactive night and day? Actigraphy studies in adult ADHD: a baseline comparison and the effect of methylphenidate | Boonstra A.M. Sandra Kooij J.J. Oosterlaan J. et al | 2007 | DSM-IV, DSM-IV ADHD rating scale, the Composite International Diagnostic Interview, International Personality Disorder Examination | Yes | Yes | Experienced psychiatrist |
| Does methylphenidate improve inhibition and other cognitive abilities in adults with childhood-onset ADHD? | Boonstra A.M. Sandra Kooij J.J., Oosterlaan J. Et al | 2005 | Diagnostic Interview Schedule , ADHD Rating Scale, Composite International Diagnostic Interview , International Personality Disorder Examination | Yes | Yes | Psychiatrist |
| The efficacy of 2 different dosages of methylphenidate in treating adults with attention-deficit hyperactivity disorder | Bouffard R., Hechtman L., Minde K. et al | 2003 | DSM-IV, Conners’Adult ADHD Rating Scale, Adult ADHD Problem Behaviours | Yes | No | Psychiatrist |
| Effects of open-label lisdexamfetamine dimesylate on self-reported quality of life in adults with ADHD | Brams M., Giblin J., Gasior M. et al | 2011 | DSM-IV | Yes | No | Unknown |
| Relationships Between Executive Function Improvement and ADHD Symptom Improvement With Lisdexamfetamine Dimesylate in Adults With ADHD and Executive Function Deficits: A Post Hoc Analysis | Brown T.E., Chen J. and Robertson B. | 2020 | DSM-IV-TR | Yes | Yes | Unknown |
| Functional improvement and correlations with symptomatic improvement in adults with attention deficit hyperactivity disorder receiving long-acting methylphenidate | Buitelaar J.K., Casas M., Philipsen A. et al | 2012 | DSM-IV, Conners’ Adult ADHD Diagnostic Interview for DSM-IV, SCID | Yes | No | Unknown |
| Predictors of treatment outcome in adults with ADHD treated with OROS® methylphenidate | Buitelaar J.K., Kooij J.J., Ramos-Quiroga J.A et al | 2011 | DSM-IV, SCID Axis I, Conners' Adult ADHD Diagnostic Interview for DSM-IV (CAADID) | Yes | No | Unknown |
| Functional magnetic resonance imaging of methylphenidate and placebo in attention-deficit/hyperactivity disorder during the multi-source interference task | Bush G., Spencer T.J., Holmes J. et al | 2008 | DSM-IV, SCID, childhood disorders by modules (DSM-IV ADHD and conduct disorder) from the Schedule for Affective Disorders and Schizophrenia for School-Age Children–Epidemiologic Version | Yes | No | Psychiatrist |
| Positive effects of transcranial direct current stimulation in adult patients with attention-deficit/hyperactivity disorder - A pilot randomized controlled study | Cachoeira C.T., Leffa D.T., Suzana Doneda Mittelstad S. et al | 2017 | DSM-5 | Yes | yes | Unknown |
| A cognitive remediation programme for adults with Attention Deficit Hyperactivity Disorder | Stevenson, C. S., Whitmont, S., Bornholt, L. Et al | 2002 | DSM-III-R, Semi-Structured Interview for Adult ADHD | Yes | Yes | Unknown |
| A controlled trial of methylphenidate in adults with attention deficit/hyperactivity disorder and substance use disorders | Carpentier P.J., de Jong C.A.J., Dijkstra B.A.G. Et al | 2005 | DSM-IV | Unknown | Yes | Unknown |
| Evaluation of the effectiveness of the FOCUS ADHD App in monitoring adults with attention-deficit/hyperactivity disorder | Carvalho L.R, Letícia M., Gregory Z. et al | 2023 | DSM-5, SCID, MINI, ADHD module of the K-SADS-E adapted for adults, ASRS-18 | Yes | Yes | Experienced psychiatrist |
| An Open-Label Extension Study Assessing the Long-Term Safety and Efficacy of Viloxazine Extended-Release Capsules in Adults with Attention-Deficit/Hyperactivity Disorder | Childress A, Cutler A.J., Adler L.A. et al | 2024 | Unknown | Unknown | No | Unknown |
| Randomized, Double-Blind, Placebo-Controlled, Parallel-Group, Adult Laboratory Classroom Study of the Efficacy and Safety of PRC-063 (Extended-Release Methylphenidate) for the Treatment of ADHD | Childress A., Cutler A.J., Marraffino A.H. et al | 2022 | SCID DSM-5, ADHD Rating Scale IV | Yes | No | Unknown |
| Methylphenidate and emotional-motivational processing in attention-deficit/hyperactivity disorder | Conzelmann A., Woidich E., Muchaet R.F. al | 2016 | DSM-IV, SCID | Yes | No | Psychiatrist |
| Cannabinoids in attention-deficit/hyperactivity disorder: A randomised-controlled trial | Cooper R.E., Williams E., Seegobin S. et al | 2017 | DSM-5, Conners' Adult ADHD Rating Scale, DIVA | Unknown | No | Unknown, but reviewed by a psychiatrist |
| Long-term efficacy of a new 6-session cognitive behavioral therapy for adults with attention-deficit/hyperactivity disorder: A randomized, controlled clinical trial. | Corrales M., García-González S., Richarte V. Et al | 2024 | DSM-5, CAARS, ADHD-RS, CGI-S, STAI, BDI, FAST and WHODAS 2.0 | Yes | Yes | Psychiatrist and Psychologist |
| A Randomized, Double-Blind, Sham- Controlled Trial of Transcranial Direct Current Stimulation in Attention-Deficit/Hyperactivity Disorder | Cosmo C., Baptista A.F., de Araújo A.N. et al | 2015 | DSM-IV-TR, MINI-plus, ASRS-18 | Yes | No | Experienced psychiatrist |
| OROS-methylphenidate or placebo for adult smokers with attention deficit hyperactivity disorder: racial/ethnic differences | Covey L.S., Hu M.C., Winhusen T. et al | 2011 | Dsm-iv, Adult Clinical Diagnostic Scale version 1.2 | Yes (but no description of how) | No | Unknown |
| Effect of stimulant medication on driving performance of young adults with attention-deficit hyperactivity disorder: a preliminary double-blind placebo controlled trial | Cox D.J., Merkel R.L., Kovatchev B. et al | 2000 | DSM-III, SCID, Barkley ADHD Structured Intreview | Yes | No | Clinician |
| Randomized, Double-Blind, Placebo-Controlled, Fixed-Dose Study to Evaluate the Efficacy and Safety of Amphetamine Extended-Release Tablets in Adults With Attention-Deficit/Hyperactivity Disorder | Cutler A.T, Childress A.C. Pardo A. et al | 2022 | DSM-5, ACDS, MINI | Yes | No | Unknown |
| Atomoxetine in adults with ADHD: two randomized, placebo-controlled studies | David Michelson D., Adler L.A., Spencer T. Et al | 2003 | DSM-IV, SCID, Conners’ Adult ADHD Diagnostic Interview for DSM-IV (CAAR-D; Conners et al 1999) | Yes | No | Unknown |
| A positron emission tomography study of nigro-striatal dopaminergic mechanisms underlying attention: implications for ADHD and its treatment | del Campo N., Fryer T.D., Hong Y.T.et al | 2013 | DSM-IV, MINI | Yes | No | Psychiatrist |
| Cognitive-behavioural therapy for adult attention-deficit hyperactivity disorder: a proof of concept randomised controlled trial | Dittner A.J., Hodsoll J., Rimes K.A. et al | 2018 | DSM-IV, MINI, Conners’ Adult ADHD Diagnostic Interview for DSM-IV | Yes | No | Unknown |
| A randomized, double-blind, crossover study of methylphenidate and lithium in adults with attention-deficit/hyperactivity disorder: preliminary findings | Dorrego M.F., Canevaro L., Gabriela Kuzis G. Et al | 2002 | DSM-IV, SCID, Structural Interview for Adult ADHD, Conners’ Adult ADHD Ratin gScale | Not mentioned | Yes | Psychiatrist |
| Double-blind, placebo-controlled, crossover study of the efficacy and safety of lisdexamfetamine dimesylate in college students with ADHD | Dupaul G.J., Weyandt L.L., Rossi J.S.et al | 2012 | DSM-IV-TR | Yes | Yes | Unknown |
| Selegiline in ADHD adults: plasma monoamines and monoamine metabolites | Ernst M., Liebenauer L.L., Tebeka D. Et al | 1997 | DSM-III-R, the Schedule for Affective Disorders and Schizophrenia | Yes | Yes | Psychiatrist |
| Atomoxetine and stroop task performance in adult attention-deficit/hyperactivity disorder | Faraone S.V., Biederman J., Spencer T. Et al. | 2005 | DSM-IV, SCID, Conners’ Adult ADHD Diagnostic In- terview for DSM-IV (CAAR-D) | Yes | No | Unknown |
| A Randomized, Double-Blind, Placebo-Controlled Trial to Evaluate the Efficacy and Safety of AR19, a Manipulation-Resistant Formulation of Amphetamine Sulfate, in Adults With Attention-Deficit/Hyperactivity Disorder | Faraone S.V., Childress A., S. et al | 2021 | DSM-IV, Conners Diagnostic Interview | Yes | No | Unknown |
| Dose response effects of lisdexamfetamine dimesylate treatment in adults with ADHD: an exploratory study | Faraone S.V., Spencer T.J., Kollins S.H.et al | 2012 | DSM-IV | Yes | Yes | Unknown |
| Pilot RCT of dialectical behavior therapy group skills training for ADHD among college students | Fleming A.P., McMahon R.J., Moran L.R. et al | 2015 | DSM-5 | Yes | Yes | Unknown |
| Assessing methylphenidate preference in ADHD patients using a choice procedure | Fredericks E.M. And Kollins S.H. | 2004 | Wender Utah rating scale, Conners Adult ADHD Rating Scale | No | Yes | Unknown |
| The mixed amphetamine salt extended release (Adderall XR, Max-XR) as an adjunctive to SSRIS or SNRIS in the treatment of adult ADHD patients with comorbid partially responsive generalized anxiety: an open-label study | Gabriel A. | 2010 | DSM-IV, MINI | Yes | Yes | Unknown |
| The reinforcing effects of nicotine and stimulant medication in the everyday lives of adult smokers with ADHD: A preliminary examination | Gehricke J.G., Whalen C.K., Jamner L.D. Et al | 2006 | DSM-IV, Wender Utah Rating Scale, Assessment of Hyperactivity and Attention (AHA) | Yes | No | Psychiatrist |
| Long-term treatment outcomes with lisdexamfetamine dimesylate for adults with attention-deficit/hyperactivity disorder stratified by baseline severity | Ginsberg L., Katic A., Adeyi B. et al | 2011 | DSM-IV-TR | Yes (but no description of how) | No | Unknown |
| Methylphenidate treatment of adult male prison inmates with attention-deficit hyperactivity disorder: randomised double-blind placebo-controlled trial with open-label extension | Ginsberg Y. and Lindefors N. | 2012 | DSM-IV, SCID, Self Rated SCID | Yes | Yes | Unknown |
| Long-term (1 year) safety and efficacy of methylphenidate modified-release long-acting formulation (MPH-LA) in adults with attention-deficit hyperactivity disorder: a 26-week, flexible-dose, open-label extension to a 40-week, double-blind, randomised, placebo-controlled core study | Ginsberg Y. Arngrim T., Philipsen A. et al | 2014 | DSM-IV ADHD RS | Yes | Unknown | Unknown |
| Attention Deficit Hyperactivity Disorder (ADHD) among longer-term prison inmates is a prevalent, persistent and disabling disorder | Ginsberg Y., Hirvikoski T. and Lindefors N. | 2010 | DSM-IV, Wender Utah Rating Scale (WURS), ASRS | Unknown | Yes | Unknown |
| Long-term functional outcome in adult prison inmates with ADHD receiving OROS-methylphenidate | Ginsberg Y., Hirvikoski T., Grann M. Et al | 2012 | SCID, Self Rated SCID, DSM-IV, ASRS | Yes | Yes | Psychiatrists, clinical psychologists. |
| An interim analysis of the Quality of Life, Effectiveness, Safety, and Tolerability (QU.E.S.T.) evaluation of mixed amphetamine salts extended release in adults with ADHD | Goodman D.W., Ginsberg L.,Weisler R.H. Et al | 2005 | DSM-IV-TR | Yes (but no description of how) | No | Unknown |
| Work-MAP Telehealth Metacognitive Work-Performance Intervention for Adults With ADHD: Randomized Controlled Trial | Grinblat N. and Rosenblum S. | 2023 | DSM-5, ASRS, Adult ADHD Self-Report Scale version 1.1-Screener | No | Yes | Self Rated |
| A Randomized Controlled Trial of Mindfulness-Based Cognitive Therapy for College Students With ADHD | Gu Y. , Xu G. and Zhu Y. | 2018 | DSM-5 | Yes | No | Psychiatrists |
| Dialectical behavioral therapy‑based group treatment versus treatment as usual for adults with attention‑deficit hyperactivity disorder: a multicenter RCT | Halmøy A., Ring A.E, Gjestad R. et al. | 2022 | DSM-IV, SCID, MINI, DIVA | Yes | No | Psychiatrist or Psychologist |
| The Efficacy of Adapted MBCT on Core Symptoms and Executive Functioning in Adults With ADHD: A Preliminary RCT | Hepark S., Janssen L., de Vries A. et al | 2019 | DSM-IV, DIVA, MINI | Yes | No | Clinician |
| Randomized clinical study of a histamine H3 receptor antagonist for the treatment of adults with attention-deficit hyperactivity disorder | Herring, W. J., Wilens, T. E., Adler, L. A. Et al | 2012 | Conners Adult ADHD Diagnostic Interview, DSM-IV, Conners Adult ADHD Rating Scale Self-Report | Yes (but no description of how) | No | Unknown |
| Long-term safety and tolerability of atomoxetine in Japanese adults with attention deficit hyperactivity disorder | Hirata Y. , Goto T., Takita Y. et al | 2014 | DSM-IV, Conners’ Adult ADHD Diagnostic Interview for DSM-IV | Yes | No | Unknown |
| Psychoeducational groups for adults with ADHD and their significant others (PEGASUS): A pragmatic multicenter and RCT | Hirvikoski T., Lindström T., Carlsson J. et al | 2017 | DSM-IV, DIVA 2.0, Wender Utah Rating Scale, ASRS | Yes | Yes | Unknown |
| Mindfulness vs psychoeducation in adult ADHD: a RCT | Hoxhaj E., Sadohara C., Borel P. et al | 2018 | DSM-IV, SCID, WURS, Conners’ Adult ADHD Rating Scales | Yes | No | Unknown |
| Effects of diurnal variation on the Test of Variables of Attention performance in young adults with attention-deficit/hyperactivity disorder | Hunt M.G., Solomon W Bienstock S.W. Et al | 2012 | Self-report | No | Yes | Unknown |
| Methylphenidate hydrochloride modified-release in adults with attention deficit hyperactivity disorder: a randomized double-blind placebo-controlled trial | Huss M., Ginsberg Y., Tvedten T. et al | 2014 | DSM-IV, DSM-IV ADHD RS | Yes | Yes | Unknown |
| Efficacy of a novel biphasic controlled-release methylphenidate formula in adults with attention-deficit/hyperactivity disorder: results of a double-blind, placebo-controlled crossover study | Jain U., Hechtman L., Margaret Weiss M. et al | 2007 | DSM-IV | Yes | No | Unknown |
| Long-term efficacy and safety outcomes with OROS-MPH in adults with ADHD | Jan K Buitelaar, Götz-Erik Trott, Maria Hofecker et al | 2012 | DSM-IV, SCID, Conners ’ Adult ADHD Diagnostic Interview for DSM-IV | Yes | No | Unknown |
| Does intensive multimodal treatment for maternal ADHD improve the efficacy of parent training for children with ADHD? A randomized controlled multicenter trial | Jans T.,  Jacob C., Warnke A. et al | 2015 | DSM-IV-TR, Wender-Utah Rating Scale, short version. | Yes | Yes | 'Local psychiatric departments'' |
| Mindfulness based cognitive therapy versus treatment as usual in adults with attention deficit hyperactivity disorder (ADHD) | Janssen L., Kan C.C., Carpentier P.J. et al | 2015 | DSM-IV-TR, DIVA, MINI-plus, SCID | Yes | Yes | Unknown |
| Effects of one single-dose methylphenidate compared to one single-dose placebo on QbTest performance in adults with untreated ADHD: a randomized controlled trial. | Jansson L, Löhman M, Östlund M, Domingo B | 2023 | DSM-5, DIVA 2.0,MINI, ASRS, Wender Utah Rating Scale, Wender-Reimerr Adult Attention-Deficit Disorder Scale | No | Yes | Clinical Psychologist |
| Dasotraline for the Treatment of Attention-Deficit/Hyperactivity Disorder: A Randomized, Double-Blind, Placebo-Controlled, Proof-of-Concept Trial in Adults | Koblan K.S., Hopkins S.C., Sarma K.et al | 2015 | DSM-IV, MINI, Conners’ Adult ADHD Diagnostic Interview | Yes | Yes | Unknown |
| Methylphenidate does not influence smoking-reinforced responding or attentional performance in adult smokers with and without attention deficit hyperactivity disorder (ADHD) | Kollins S.H. , Schoenfelder E., English J.S.et al | 2013 | SCID, Conners Adult ADHD Diagnostic Interview for ADHD, Conners Adult ADHD Rating Scale | Unknown | Yes | 'Licensed clinician'' |
| Sustained release methylphenidate for the treatment of ADHD in amphetamine abusers: a pilot study | Konstenius M., Jayaram-Lindström N., Beck O. et al | 2010 | Conners’ adult ADHD self and observer rating scales (CAARS:SV and CAARS:O) | Yes | No | Unknown |
| Efficacy and safety of methylphenidate in 45 adults with attention-deficit/hyperactivity disorder. A randomized placebo-controlled double-blind cross-over trial | Kooij J.J.S., Burger H., Boonstra A.M. Et al | 2004 | DSM-IV ADHD rating scale, CIDI, DISL-O, IPDE | Yes | No | Psychiatrist |
| Influence of striatal dopamine transporter availability on the response to methylphenidate in adult patients with ADHD | Krause J., la Fougere C., Krause K.H. Et al | 2005 | DSM-IV, ''semi-structured interviews'' | Yes | No | Unknown |
| Bupropion SR vs. methylphenidate vs. placebo for attention deficit hyperactivity disorder in adults | Kuperman S., Perry P.J., Gaffney G.R. Et al | 2001 | DSM-IV, SCID DSM-III-R | Yes | No | Physician |
| Long-term Effects of Multimodal Treatment on Adult Attention-Deficit/Hyperactivity Disorder Symptoms: Follow-up Analysis of the COMPAS Trial | Lam A.P., Matthies S., Graf E. et al | 2019 | DSM‐IV criteria | Yes | No | Unknown |
| Transcranial Direct Current Stimulation vs Sham for the Treatment of Inattention in Adults With Attention-Deficit/Hyperactivity Disorder: The TUNED Randomized Clinical Trial | Leffa D.T. Grevet E.H. , Claiton H.D.B. et al | 2022 | DSM-5, SCID, CASRS | No | Yes | Unknown |
| Neurophysiologic predictors of response to atomoxetine in young adults with attention deficit hyperactivity disorder: a pilot project | Leuchter A.F. ,  McGough J.J.,  Korb A.S.et al | 2014 | Adult ADHD Clinician Diagnostic Scale | Yes | No | Unknown |
| Nicotine effects on adults with attention-deficit/hyperactivity disorder | Levin E.D., Conners C.K., Sparrow E. Et al | 1996 | Wender Utah Rating Scale, the Conners/Wells Adolescent and Adult Self-Report, a modified version of Barkley's Adult A D H D Semi-structured Interview (Barkley | No | Unknown | Unknown |
| Effects of major depressive disorder and attention-deficit/hyperactivity disorder on the outcome of treatment for cocaine dependence | Levin F.R. , Bisaga A., Raby W. et al | 2008 | DSM-IV, SCID (Axis I), the KSCID module for childhood and adult ADHD (Hien 1994) | Yes | Yes | Doctoral or masters level clinical psychologists |
| Treatment of cocaine dependent treatment seekers with adult ADHD: double-blind comparison of methylphenidate and placebo | Levin F.R., Evans S.M., Brooks D.J. et al | 2007 | DSM-IV, the Wender Utah Rating Scale (WURS), the Adult ADHD Rating Scale (AARS), SCID, KID-SCID (KSCID), a structured module designed for use in childhood ADHD that was modified for use in adult ADHD | Yes | Yes | Clinicians who had either a Ph.D., Psy.D., or M.A. in Clinical Psychology. |
| Treatment of methadone-maintained patients with adult ADHD: double-blind comparison of methylphenidate, bupropion and placebo | Levin F.R., Evans S.M., Brooks D.J. Et al | 2006 | DSM-IV, SCID | Yes | No | Unknown |
| Bupropion treatment for cocaine abuse and adult attention-deficit/hyperactivity disorder | Levin F.R., Evans S.M., McDowell D.M. Et al | 2002 | The Utah Criteria, SCID, KID-SCID | Yes | Yes | Masters level psychologist or psy- chiatrist |
| Extended-Release Mixed Amphetamine Salts for Comorbid Adult Attention-Deficit/Hyperactivity Disorder and Cannabis Use Disorder: A Pilot, Randomized Double-Blind, Placebo-Controlled Trial. | Levin F.R., Mariani J.J., Pavlicova M. Et al | 2024 | DSM-5, MINI, DIVA | Yes | Yes | Unknown |
| Exploring the impact of probiotics on adult ADHD management through a double-blind RCT | Levy Schwartz M, Magzal F, Yehuda I, Tamir S. | 2024 | MATAL (computer test) | No | Yes | Computer confirmed by a neurologist or psychiatrists |
| EEG alpha power during maintenance of information in working memory in adults with ADHD and its plasticity due to working memory training: A RCT | Liu Z.X., Glizer D., Tannock R. et al | 2016 | Adult ADHD Self-Report Scale | No | Yes | Unknown |
| Contrasting expectancy effects with objective measures in adults with untreated ADHD during QbTest | Löhman M., Domingo B., Östlund M. Et al, | 2023 | DSM-5, DIVA 2.0, ASRS, BRIEF-A, Wender Utah Rating Scale, Wender-Reimerr Adult Attention-Deficit Disorder Scale | No | Yes | Unknown |
| Exploring the efficacy of dialectical behaviour therapy and methylphenidate on emotional comorbid symptoms in adults with attention Deficit/Hyperactivity disorder: Results of the COMPAS multicentre randomised controlled trial. | López-Pinar C, Selaskowski B, Braun N. Et al | 2023 | DSM-IV, SCID, WURS, ADHS-DC | Yes | Yes | Psychiatrist |
| Exploring the Relationship between Adherence to Therapy, Treatment Acceptability, and Clinical Outcomes in Adults with Attention-Deficit/Hyperactivity Disorder: Results from the COMPAS Multicenter Randomized Controlled Trial. | López-Pinar C., Rosen H., Selaskowski B. Et al | 2024 | DSM-IV, SCID, WURS, ADHS-DC | Yes | Yes | Psychiatrist |
| Toward personalized smoking-cessation treatment: Using a predictive modeling approach to guide decisions regarding stimulant medication treatment of attention-deficit/hyperactivity disorder (ADHD) in smokers | Luo S.X., Covey L.S., Hu M.C. et al | 2015 | DSM-IV, Adult Clinical Diagnostic Scale version 1.2 | Yes | Yes | Unknown |
| Exploring longitudinal course and treatment-baseline severity interactions in secondary outcomes of smoking cessation treatment in individuals with attention-deficit hyperactivity disorder | Luo S.X., Wall M., Covey L. et al | 2018 | DSM-IV, Adult Clinical Diagnostic Scale version 1.2 | Yes | No | Unknown |
| A Randomized, Phase 3, Double-Blind, Crossover Comparison of Multilayer, Extended-Release Methylphenidate (PRC-063), and Lisdexamfetamine in the Driving Performance of Young Adults With ADHD. | Madaan V., Bhaskar S., Donnelly G.A.E., Cox D.J.. | 2024 | DSM-5, SCID | Yes | Yes | Trained rater |
| Attention benefits after a single dose of metadoxine extended release in adults with predominantly inattentive ADHD | Manor I., Rubin J., Daniely Y. Et al | 2014 | DSm-IV, SCID, Adult ADHD Clinical Diagnostic Scale | Yes | No | Unknown |
| Long-term open-label response to atomoxetine in adult ADHD: influence of sex, emotional dysregulation, and double-blind response to atomoxetine | Marchant B.K., Reimherr F.W., Halls C. et al | 2011 | Conners’ Adult ADHD Diagnostic Interview for DSM-IV, DSM-IV | No | Unknown | Unknown |
| Working Memory Training in ADHD: Controlling for Engagement, Motivation, and Expectancy of Improvement (Pilot Study) | Mawjee K., Woltering S., Lai N. al | 2014 | Adult ADHD Self-Report Scale | Yes | Yes | Unknown |
| Working Memory Training in Post-Secondary Students with ADHD: A Randomized Controlled Study | Mawjee K., Woltering S. and Tannock R. | 2015 | Adult ADHD Self-Report Scale (ASRS-A Interview), Adult ADHD Self-Report Scale (ASRS-A Interview) | No | Yes | Self Rated |
| A placebo-controlled trial of atomoxetine in marijuana-dependent individuals with attention deficit hyperactivity disorder | McRae-Clark A.L., Carter R.E., Killeen T.K.et al | 2010 | DSM-IV, SCID, Conners’ Adult ADHD Diagnostic Interview | Yes | Yes | Unknown |
| A randomized, placebo-controlled trial of three fixed dosages of prolonged-release OROS methylphenidate in adults with attention-deficit/hyperactivity disorder | Medori R. , Ramos-Quiroga J.A.,Casas M. et al | 2008 | DSM-IV, SCID, Conners’ Adult ADHD Diagnostic Interview for DSM-IV (CAADID), | Yes | No | Unknown |
| Motivational interviewing plus behavioral activation for alcohol misuse in college students with ADHD | Meinzer M.C., Oddo L.E., Vasko J.M. et al | 2021 | DSM-5, SCID, Adult ADHD Clinical Diagnostic Scale | Yes | Yes | Advanced-undergraduate, masters-, doctoral-levela nd postdoctoral trainees. |
| Assessing the validity of the Quality of Life Enjoyment and Satisfaction Questionnaire Short Form in adults with ADHD | Mick E., Faraone S.V.,  Spencer T. et al | 2008 | DSM-IV, SCID, supplemented for childhood disorders by modules (DSM-IV ADHD and conduct disorder) from the Kiddie Schedule for Affective Disorder and Schizophrenia for School-Age Children–Epidemiologic Version structured diagnostic interview | Yes | Yes | Unknown |
| Absence of association with DAT1 polymorphism and response to methylphenidate in a sample of adults with ADHD | Mick E., Joseph Biederman J., Spencer T. Et al | 2006 | DSM-IV, confirmed by structured diagnostic interview. | Yes | Yes | Unknown |
| Feasibility trial of the dialectical behavior therapy skills training group as add-on treatment for adults with attention-deficit/hyperactivity disorder | Moritz G.R., Pizutti L.T., Cancian A.C.M. et al | 2021 | DSm-5 | Yes | Yes | Unknown |
| A Phase III, Randomized, Double‑Blind, Placebo‑Controlled Trial Assessing the Efficacy and Safety of Viloxazine Extended‑Release Capsules in Adults with Attention‑Deficit/Hyperactivity Disorder | Nasser A:, Hull J.T., Chaturvedi S.A. et al | 2022 | DSM-5, SCID | Yes | Yes | Unknown |
| Neurobiological basis of reinforcement-based decision making in adults with ADHD treated with lisdexamfetamine dimesylate: Preliminary findings and implications for mechanisms influencing clinical improvement. | Newcorn J.H., Ivanov I., Krone B. Et al | 2024 | DSM-5, SCID, Adult ADHD Clinical Diagnostic Scale version 1.2 | No | No | Unknown |
| Atomoxetine could improve intra-individual variability in drug-naïve adults with attention-deficit/hyperactivity disorder comparably with methylphenidate: A head-to-head randomized clinical trial | Ni H.C., Hwang Gu S.L., Lin H.Y. et al | 2016 | Adult ADHD Self-Report Scale-v1.1, Chinese version of the Schedule for Affective Disorders and Schizophrenia– Epidemiological Version (K-SADS-E) | Yes | No | Telephone interviews by board-certified child psychiatrist and then further clinical assessment |
| Mixed-amphetamine salts increase abstinence from marijuana in patients with co-occurring attention-deficit/hyperactivity disorder and cocaine dependence | Notzon D.P., Mariani J.J.,  Pavlicova M. Et al | 2016 | Conners Adult ADHD Diagnostic Interview, SCID, DSM-IV | Yes | No | Unknown |
| Treating nicotine dependence by targeting attention-deficit/ hyperactivity disorder (ADHD) with OROS methylphenidate: the role of baseline ADHD severity and treatment response | Nunes E.V, Covey L.S., Brigham G. et al | 2013 | DSM-IV, Adult Clinical Diagnostic Scale, DSM-IV ADHD Rating Scale | Unknown | No | Unknown |
| One-year follow-up of the effectiveness and mediators of cognitive behavioural therapy among adults with attention-deficit/hyperactivity disorder: secondary outcomes of a randomised controlled trial | Pan M.R., Dong M., Zhang S.Y. Et al | 2024 | DSM-5, Conners’ Adult ADHD Diagnostic Interview for DSM-IV, SCID, ADHD-RS | Yes | Yes | Psychiatrist |
| A randomised, double-blind, placebo-controlled trial of dexamphetamine in adults with attention deficit hyperactivity disorder | Paterson R., Douglas C., Hallmayer J. Et al | 1999 | 'DSM-IV ADHD symptom checklist'' (self rated) | Yes | No | Psychiatrist |
| A controlled study of methylphenidate in the treatment of attention deficit disorder, residual type, in adults | Paul H. Wender P.H., Reimherr F.W. And Wood D. | 1985 | The Utah Criteria | Yes | Yes | Unknown |
| Randomised sham-controlled study of high-frequency bilateral deep transcranial magnetic stimulation (dTMS) to treat adult attention hyperactive disorder (ADHD): Negative results | Paz Y., Friedwald K., Yeheal L. et al | 2018 | DSM5 criteria, The Wender Utah ADHD Rating Scale (WURS), Adult ADHD Self Report Scale (ASRS) | Yes | No | Psychiatrist |
| Internet-Based Cognitive Behavioral Therapy for Adults With ADHD in Outpatient Psychiatric Care | Pettersson R., Söderström S., Edlund-Söderström K. et al | 2014 | DSM-IV-TR | Yes | Yes | Unknown |
| Effects of Group Psychotherapy, Individual Counseling, Methylphenidate, and Placebo in the Treatment of Adult Attention-Deficit/Hyperactivity Disorder: A Randomized Clinical Trial | Philipsen A., Jans T., Graf E. et al | 2015 | DSM-IV, Wender-Utah-Rating-Scale, SCID, ADHD diagnostic checklist | Yes | Yes | 'Psychiatric expert assessment'' |
| Effects of mindfulness-based cognitive therapy on neurophysiological correlates of performance monitoring in adult attention-deficit/hyperactivity disorder | Poppy L A Schoenberg P.L.A., Hepark S., Kan C.C. Et al | 2014 | DSM-IV-TR | Yes | Yes | Psychiatrists |
| AZD3480, a novel nicotinic receptor agonist, for the treatment of attention-deficit/hyperactivity disorder in adults | Potter A.S., Dunbar G., Mazzulla E. et al | 2013 | DSM-IV, SCID | Yes | No | Unknown |
| The Use of Brexpiprazole Combined With a Stimulant in Adults With Treatment-Resistant Attention-Deficit/Hyperactivity Disorder | Reimherr F, W. Gift, Steans T.E.et al | 2022 | DSM-5, Conners' AdultADHD Diagnostic Interview (CAADID), MINI | Yes | Yes | Unknown |
| Emotional dysregulation in adult ADHD and response to atomoxetine | Reimherr F.W., Marchant B.K., Strong R.E., et al | 2005 | DSM-IV, Conners’ Adult ADHD Diagnostic Interview for DSM-IV (CAARS-D), SCID | Yes | No | Unknown |
| Multiscale assessment of treatment efficacy in adults with ADHD: a randomized placebo-controlled, multi-centre study with extended-release methylphenidate | Retz W., Rösler M, Ose C. et al | 2012 | DSM-IV, SCID, short version of the Wender Utah Rating scale | Yes | No | Unknown, but reviewed by a commitee of psychiatrists |
| Mixed amphetamine salts extended-release in the treatment of adult ADHD: a randomized, controlled trial | Richard H Weisler, Joseph Biederman, Thomas J Spencer T.J. Et al | 2006 | DSM-IV-TR | Unknown | No | Unknown |
| Baseline brain volume predicts home-based transcranial direct current stimulation effects on inattention in adults with attention-deficit/hyperactivity disorder. | Rodrigues da Silva P.H., Leffa D.T., Luethi M.S. Et al | 2024 | DSM-5, ''Semi-structured clinical interview'', Kiddie SADS, SCID | Yes | No | Psychiatrist |
| Cognitive-behavioral therapy for ADHD in medication-treated adults with continued symptoms | Safren S.A.., Otto M.W., Sprich S. Et al | 2005 | DSM-IV, SCID, supplemented by sections of the Kiddie Schedule for Affective Disorders and Schizophrenia- Epidemiologic Version (Kiddie-SADS-E) | Yes | yes | Licensed clinical psychologist |
| Cognitive behavioral therapy vs relaxation with educational support for medication-treated adults with ADHD and persistent symptoms: a RCT | Safren S.A. , Sprich S., Mimiaga M.J. et al | 2010 | DSM-IV, SCID, Kiddie Schedule for Affective Disorders and Schizophrenia Epidemiologic Version to assess ADHD. | Yes | Yes | Study therapists |
| Working memory training restores aberrant brain activity in adult attention-deficit hyperactivity disorder | Salmi J., Soveri A., Salmela V. et al | 2020 | SCID (DSM-IV), MINI, Conners' Adult ADHD Diagnostic Interview | Yes | Yes | Unknown |
| Lisdexamfetamine Targets Amygdala Mechanisms That Bias Cognitive Control in Attention-Deficit/Hyperactivity Disorder | Schulz K.P., Krone B., Adler L.A. , Bédard A.C.V. et al | 2018 | DSM-IV, SCID, Adult ADHD Clinical Diagnostic Scale | Yes | No | Psychologist |
| Smartphone-assisted psychoeducation in adult attention-deficit/hyperactivity disorder: A RCT | Selaskowski B, , Steffens M. , Schulze M. et al | 2022 | DSM-5, SCID, Integrated Diagnosis of ADHD in Adulthood | Yes | Yes | Unknown |
| A neurocomputational account of reward and novelty processing and effects of psychostimulants in attention deficit hyperactivity disorder | Sethi A., Voon V., Critchley H.D. et al | 2018 | DSM-IV, DIVA, Conners’ ADHD self-report long version, Wender Utah questionnaires | Yes | Yes | Unknown |
| Sleep in adults with attention deficit hyperactivity disorder (ADHD) before and during treatment with methylphenidate: a controlled polysomnographic study | Sobanski E., el Schredl M., Kettler N. Et al | 2008 | DSM-IV, ''structured clinical interview'' | Yes | No | Senior psychiatrist |
| Treatment adherence and persistence in adult ADHD: results from a twenty-four week controlled clinical trial with extended release methylphenidate | Sobanski E., Retz W., Fischer R., Rösler M. et al | 2013 | DSM-IV-TR, ADHD-Diagnostic Checklist , Wender Utah rating scale, Conners adult ADHD rating scale self-report, Symptom Checklist 90 revised version | Unknown | Yes | Unknown |
| Efficacy and safety of atomoxetine hydrochloride in Korean adults with attention-deficit hyperactivity disorder | Soyoung Irene Lee S.I., Song D.H.,  Shin D.V. et al | 2014 | DSM-IV-TR, Conners’ Adult ADHD Rating Scale-Investigator-rated | Yes | Yes | Unknown |
| Effectiveness and tolerability of tomoxetine in adults with attention deficit hyperactivity disorder | Spencer T., Biederman J., Wilens T. Et al | 1998 | DSM-III-R | Yes | Yes | Unknown |
| A large, double-blind, randomized clinical trial of methylphenidate in the treatment of adults with attention-deficit/hyperactivity disorder | Spencer T., Biederman J., Wilens T. et al | 2005 | DSM-IV, SCID, childhood disorders by modules (DSM-IV ADHD and conduct disorder) from the Kiddie SADS-E (Epidemiologic Versio) | Yes | Yes | Trained rater |
| A double-blind, crossover comparison of methylphenidate and placebo in adults with childhood-onset attention-deficit hyperactivity disorder | Spencer T., Wilens T., Biederman J. Et al | 1995 | Structured Clinical Interview for DSM-III-R and DSM-IV, supplemented for childhood disorders by unmodified modules from the Schedule for Affective Disorders and Schizophrenia for School-Age Children—Epidemiologic Version (24). | Yes | Yes | Trained rater |
| Efficacy and safety of dexmethylphenidate extended-release capsules in adults with attention-deficit/hyperactivity disorder | Spencer T.J., Adler L.A., McGough J.J. et al | 2007 | DSM-IV | Yes (but no description of how) | Yes | Unknown |
| Triple-bead mixed amphetamine salts (SPD465), a novel, enhanced extended-release amphetamine formulation for the treatment of adults with ADHD: a randomized, double-blind, multicenter, placebo-controlled study | Spencer T.J., Adler L.A., Weisler R.H. Et al | 2008 | DSM-IV, ADHD-RS | Yes | No | Clinician |
| Opiate Antagonists Do Not Interfere With the Clinical Benefits of Stimulants in ADHD: A Double-Blind, Placebo-Controlled Trial of the Mixed Opioid Receptor Antagonist Naltrexone | Spencer T.J., Bhide P., Zhu J. et al | 2018 | DSM-IV, AISRS | Yes | No | Unknown |
| Atomoxetine and adult attention-deficit/hyperactivity disorder: the effects of comorbidity | Spencer T.J., Faraone S.V., Michelson D. Et al | 2006 | DSM-IV, Clinical Interview, SCID, Conners’ Adult ADHD Diagnostic Interview for DSM-IV (CAAR-D; Conners et al 1999) | Yes | No | Unknown |
| A randomized, single-blind, substitution study of OROS methylphenidate (Concerta) in ADHD adults receiving immediate release methylphenidate | Spencer T.J., Mick E., Surman C.B.H. et al | 2011 | DSM-IV, SCID, Childhood disorders by modules (DSM-IV ADHD and conduct disorder) from the Kiddie SADSE | Yes | Yes | Psychiatrist |
| Predicting efficacy of viloxazine extended-release treatment in adults with ADHD using an early change in ADHD symptoms: Machine learning Post Hoc analysis of a phase 3 clinical trial | Faraone S.V., Gomeni R., Hull J.T. et al | 2022 | DSM-5, SCID, AISRS | Yes | Yes | Unknown |
| The Efficacy of Computerized Cognitive Training in Adults With ADHD: A RCT | Stern A., Malik E., Pollak Y. et al | 2016 | SCID | Yes | Yes | Psychiatrist |
| Near and Far Transfer Effects of Computerized Progressive Attention Training (CPAT) Versus Mindfulness Based Stress Reduction (MBSR) Practice Among Adults With ADHD | Stern P., Kolodny T., Tsafrir S. et al | 2023 | DSM-5 | Yes | No | Psychiatrist |
| Solriamfetol for Attention-Deficit/Hyperactivity Disorder in Adults: A Double-Blind Placebo-Controlled Pilot Study. | Surman C.B.H., Walsh D.M., Horick N. Et al | 2023 | DSM-5, AISRS, Adult Self Report (ASR) | Yes | No | Psychiatrist |
| An 8-week, RCT of atomoxetine, atomoxetine plus buspirone, or placebo in adults with ADHD | Sutherland S.M., Adler L.A., Chen C. et al | 2012 | DSM-IV-TR, ACDS | Yes | Yes | Unknown |
| Randomized, double-blind, placebo-controlled, crossover study of the effects of lisdexamfetamine dimesylate and mixed amphetamine salts on cognition throughout the day in adults with attention-deficit/hyperactivity disorder | T Martin P.T. , Corcoran M., Zhang P. et al | 2014 | Adult ADHD Clinical Diagnostic Scale, version 1.2, (ADHD-RS-IV), MINI | Yes | Yes | Psychiatrist |
| Efficacy of a mixed amphetamine salts compound in adults with attention-deficit/hyperactivity disorder | T Spencer, J Biederman, T Wilens, S | 2001 | DSM-IV, SCID, supplemented for childhood disorders by unmodified modules from the Kiddie Schedule for Affective Disorders and Schizophrenia-Epidemiological Version (KSADS-E) | Yes | Yes | Psychiatrist |
| A randomized, double-blind, placebo-controlled, parallel-group study to evaluate the efficacy and safety of osmotic-controlled release oral delivery system methylphenidate HCl in adults with attention-deficit/hyperactivity disorder in Japan | Takahashi N., Koh T., Tominaga Y. et al | 2014 | DSM-IV, Conners ’ Adult ADHD Diagnostic Interview for DSM-IV | Yes | No | Unknown |
| Efficacy of modafinil compared to dextroamphetamine for the treatment of attention deficit hyperactivity disorder in adults | Taylor F.B. and Russo J. | 2000 | DSM-IV, ADHD Behavior Checklist for Adults, Beck Depression Inventory, Hamilton Anxiety Rating Scale | Yes | Yes | Unknown |
| Comparing guanfacine and dextroamphetamine for the treatment of adult attention-deficit/hyperactivity disorder | Taylor F.B. and Russo J. | 2001 | DSM-IV, the ADHD Behavior Checklist for Adults | Yes (but no description of how) | Yes | Unknown |
| An experimental comparison of Pycnogenol and methylphenidate in adults with Attention-Deficit/Hyperactivity Disorder (ADHD) | Tenenbaum S., Paull J.C., Sparrow E.P. Et al | 2002 | DSM-IV, SCID, Semi-Structured Clinical Interview for ADHD, Copeland Symptom Checklist for Adult Attention Disorders, Brown ADD Scales | Yes | Yes | 'Clinical interview with a licensed clinician'' |
| Working memory related functional connectivity in adult ADHD and its amenability to training: A randomized controlled trial. | Tolonen T., Leppämäki S., Roine T. Et al | 2024 | DSM-5, Conners’ Adult ADHD Diagnostic Interview for DSM-IV, MINI, SCID | Yes | No | Psychiatrist |
| Modafinil improves cognition and response inhibition in adult attention-deficit/hyperactivity disorder | Turner D.C., Clark L., Dowson J et al | 2004 | DSM-IV, ''self ratings and observer scales from childhood'' | Yes | Yes | Psychiatrist |
| Neurocognitive effects of methylphenidate in adult attention-deficit/hyperactivity disorder | Turner D.C., Blackwell A.D., Dowson J.H. et al | 2005 | DSM-IV | Yes | No | Psychiatrist |
| Time-to-onset and -resolution of adverse events before/after atomoxetine discontinuation in adult patients with ADHD | Upadhyaya H., Tanaka Y. , Lipsius S., Kryzhanovskaya L.A. et al | 2015 | DSM-IV, Conners’ Adult ADHD Diagnostic Interview for DSM-IV-TR | Yes | No | Unknown |
| Effects of chronotherapy on circadian rhythm and ADHD symptoms in adults with attention-deficit/hyperactivity disorder and delayed sleep phase syndrome: a randomized clinical trial | van Andel E., Bijlenga D., Vogel S.W.N. et al | 2021 | MINI, DIVA | Yes | No | Psychiatrist |
| Effects of Chronotherapeutic Interventions in Adults With ADHD and Delayed Sleep Phase Syndrome (DSPS) on Regulation of Appetite and Glucose Metabolism. | van Andel E., Vogel S.W.N., Bijlenga D. Et al | 2024 | DSM-IV, DIVA, MINI | Yes | No | Psychiatrist |
| Methylphenidate increases cigarette smoking in participants with ADHD | Vansickel A.R., Stoops W.W., Glaser P.E.A. et al | 2011 | DSM-IV, Conners’ Adult ADHD Diagnostic Interview for DSM-IV, | Yes | No | Psychiatrist |
| Methylphenidate significantly reduces lapses of attention during on-road highway driving in patients with ADHD | Verster J.C., Roth T. | 2014 | DSM-IV, DSM-IV ADHD-rating scale | Yes | No | Psychiatrists |
| Psychoeducation for adults with attention deficit hyperactivity disorder vs. cognitive behavioral group therapy: a randomized controlled pilot study | Vidal R., Bosch R., Nogueira M. et al | 2013 | DSM-Iv, ADHD Rating Scale, SCID, Conners’ Adult ADHD Diagnostic Interview for DSM-IV, the ADHD-RS, and Conners’ Adult ADHD Rating ScaleYSelf- Report: Long Version | Yes | No | Psychiatrists and psychologists |
| Long-term safety and effectiveness of lisdexamfetamine dimesylate in adults with attention-deficit/ hyperactivity disorder | Weisler R., Young J., Mattingly C. et al | 2009 | DSM-IV-TR criteria | Yes | Yes | Unknown |
| Long-term cardiovascular effects of mixed amphetamine salts extended release in adults with ADHD | Weisler R.H., Biederman J., Spencer T.J et al | 2005 | DSM-IV-TR | Yes (but no description of how) | No | Unknown |
| Efficacy and Safety of SHP465 Mixed Amphetamine Salts in the Treatment of Attention-Deficit/Hyperactivity Disorder in Adults: Results of a Randomized, Double-Blind, Placebo-Controlled, Forced-Dose Clinical Study | Weisler R.H., Greenbaum M., Arnold V. et al | 2017 | DSM-5, ADHD Rating Scale with Adult Prompts (ADHD- RS-AP | Yes | No | Unknown |
| A RCT of CBT therapy for adults with ADHD with and without medication | Weiss M.,  Murray C., Wasdell M. et al | 2012 | DSM-IV, SCID, Conners’ ADHD Diagnostic Interview | Yes | Yes | Unknown |
| Effect of a Multi-Layer, Extended-Release Methylphenidate Formulation (PRC-063) on Sleep in Adults with ADHD: A Randomized, Double-Blind, Forced-Dose, Placebo-Controlled Trial Followed by a 6-month Open-Label Extension | Weiss M.D., Surman C., Khullar A. et al | 2021 | ADHD-5-RS, DSM-5 | Yes | No | Unknown |
| Attention deficit disorder ('minimal brain dysfunction') in adults. A replication study of diagnosis and drug treatment | Wender P.H., Reimherr F.W. and Wood D.R. | 1981 | The "Utah Criteria", DSM-III | Yes | Yes | Unknown |
| Atomoxetine once daily for 24 weeks in adults with attention-deficit/hyperactivity disorder (ADHD): impact of treatment on family functioning | Wietecha L. , Young J., Ruff D. et al | 2012 | DSM-IV-TR, Conners Adult ADHD Diagnostic Interview | No | Unknown | Unknown |
| The Time Course of Effect of Multilayer-Release Methylphenidate Hydrochloride Capsules: A Randomized, Double-Blind Study of Adults With ADHD in a Simulated Adult Workplace Environment | Wigal S.B., Wigal Ann Childress A. et al | 2020 | Conners’s Adult ADHD Diagnostic Interview, DSM-5 | No | Yes | Unknown |
| Controlled trial of high doses of pemoline for adults with attention-deficit/hyperactivity disorder | Wilens T.E., Biederman J., Spencer T.J. Et al | 1999 | DSM-III-R, SCID, Kiddie Schedule for Affective Disorders and Schizophrenia-Epidemiologic Version. | Yes | No | Psychiatrist |
| Blood pressure changes associated with medication treatment of adults with attention-deficit/hyperactivity disorder | Wilens T.E., Hammerness P.G., Biederman J. Et al | 2005 | DSm-III, SCID, Kiddie | Yes | No | Unknown |
| Six-week, double-blind, placebo-controlled study of desipramine for adult attention deficit hyperactivity disorder | Wilens T.E., Biederman J., Prince J. Et al | 1996 | Structured Clinical Interview for SM-III-R, supplemented for childhood disorders by unmodified modules from the Schedule for Affective Disorders and Schizophrenia for School-Age Children- Epidemiologic Version | Yes | Yes | Trained rater |
| A pilot controlled clinical trial of ABT-418, a cholinergic agonist, in the treatment of adults with attention deficit hyperactivity disorder | Wilens T.E., Biederman J., Spencer T.J. Et al | 1999 | Structured Clinical Interview for DSM-III-R and DSM-IV, supplemented for childhood disorders by unmodified modules from the Schedule for Affective Disorders and Schizophrenia for School-Age Children—Epidemiologic Version (24). | Yes | Yes | Trained rater |
| Bupropion XL in adults with attention-deficit/hyperactivity disorder: a randomized, placebo-controlled study | Wilens T.E., Haight B.R., Horrigan J.P. Et al | 2005 | DSM-IV, SCID, Schedule for Affective Disorders and Schizophrenia for School-Age Children, Epidemiologic Version | Yes | No | Unknown |
| A controlled clinical trial of bupropion for attention deficit hyperactivity disorder in adults | Wilens T.E., Spencer T.J., Biederman J. et al | 2001 | Structured Clinical Interview for DSM-III-R and DSM-IV, supplemented for childhood disorders by unmodified modules from the Schedule for Affective Disorders and Schizophrenia for School-Age Children—Epidemiologic Version (24). | Yes | Yes | Trained rater |
| ABT-089, a neuronal nicotinic receptor partial agonist, for the treatment of attention-deficit/hyperactivity disorder in adults: results of a pilot study | Wilens T.E., Verlinden M.H., Adler L.A. Et al | 2006 | DSM-IV-TR, Kiddie SADS diagnostic criteria for ADHD | Yes | Yes | Unknown |
| Diagnosis and treatment of minimal brain dysfunction in adults: a preliminary report | Wood D.R., Reimherr F.W. and Wender P.H. | 1976 | Research Diagnostic Criteria, self-report form | Yes | Unknown | Unknown |
| Neural Correlates of Symptom Improvement Following Stimulant Treatment in Adults with Attention-Deficit/Hyperactivity Disorder | Yang Z., Kelly C., Castellanos F.X. et al | 2016 | DSM-IV-TR, Adult Clinician Diagnostic Scale (ACDS), SCID | Yes | Yes | Unknown |
| Cognitive-behavioural therapy in medication-treated adults with attention-deficit/hyperactivity disorder and co-morbid psychopathology: a RCT using multi-level analysis | Young S., Khondoker M., Emilsson B. et al | 2015 | DSM-IV, MINI | Yes | Yes | "Experienced mental health practitioner" |
| fMRI Neurofeedback Training for Increasing Anterior Cingulate Cortex Activation in Adult Attention Deficit Hyperactivity Disorder. An Exploratory Randomized, Single-Blinded Study | Zilverstan A., Sorger B, Slaats-Willemse D. et al | 2017 | DSM-IV TR, DIVA, ADHD-DSM-IV Rating Scale | Yes | No | Unknown |
| The effects of acute tryptophan depletion on reactive aggression in adults with attention-deficit/hyperactivity disorder (ADHD) and healthy controls | Zimmermann M., Grabemann M., Mette C. et al | 2012 | Wender Utah Rating Scale (WURS) | Yes (but no description of how) | No | Unknown |
