# Supplementary material for: Diagnosing ADHD in adults in randomized controlled studies: a scoping review
Source: Eur Psychiatry. 2025 Apr 14;68(1):e64. doi: 10.1192/j.eurpsy.2025.2447 (PMC12188335; doi:10.1192/j.eurpsy.2025.2447)
Supplement: Studart et al. supplementary material 2 — Studart et al. supplementary material [file S0924933825024472sup002.docx]

**Supplementary 2. References to the 292 included randomized controlled studies**

1. Aarts, E., van Holstein, M., Hoogman, M., Onnink, M., Kan, C., Franke, B., ... & Cools, R. (2015). Reward modulation of cognitive function in adult attention-deficit/hyperactivity disorder: a pilot study on the role of striatal dopamine. *Behavioural pharmacology*, *26*(1 and 2-Special Issue), 227-240.
2. Addicott, M. A., Pearson, J. M., Schechter, J. C., Sapyta, J. J., Weiss, M. D., & Kollins, S. H. (2021). Attention-deficit/hyperactivity disorder and the explore/exploit trade-off. *Neuropsychopharmacology*, *46*(3), 614-621.
3. Adler, L. A., Adams, J., Madera-McDonough, J., Kohegyi, E., Hobart, M., Chang, D., ... & Liebowitz, M. (2022). Efficacy, safety, and tolerability of centanafadine sustained-release tablets in adults with attention-deficit/hyperactivity disorder: Results of 2 phase 3, randomized, double-blind, multicenter, placebo-controlled trials. *Journal of Clinical Psychopharmacology*, *42*(5), 429-439.
4. Adler, L. A., Alperin, S., Leon, T., & Faraone, S. V. (2017). Pharmacokinetic and pharmacodynamic properties of lisdexamfetamine in adults with attention-deficit/hyperactivity disorder. *Journal of Child and Adolescent Psychopharmacology*, *27*(2), 196-199.
5. Adler, L. A., Alperin, S., Leon, T., & Faraone, S. V. (2017). Pharmacokinetic and pharmacodynamic properties of lisdexamfetamine in adults with attention-deficit/hyperactivity disorder. *Journal of Child and Adolescent Psychopharmacology*, *27*(2), 196-199.
6. Adler, L. A., Clemow, D. B., Williams, D. W., & Durell, T. M. (2014). Atomoxetine effects on executive function as measured by the BRIEF-A in young adults with ADHD: A randomized, double-blind, placebo-controlled study. *PLoS One*, *9*(8), e104175.
7. Adler, L. A., Dirks, B., Deas, P. F., Raychaudhuri, A., Dauphin, M. R., Lasser, R. A., & Weisler, R. H. (2013). Lisdexamfetamine dimesylate in adults with attention-deficit/hyperactivity disorder who report clinically significant impairment in executive function: results from a randomized, double-blind, placebo-controlled study. *The Journal of clinical psychiatry*, *74*(7), 10397.
8. Adler, L. A., Dirks, B., Deas, P., Raychaudhuri, A., Dauphin, M., Saylor, K., & Weisler, R. (2013). Self-reported quality of life in adults with attention-deficit/hyperactivity disorder and executive function impairment treated with lisdexamfetamine dimesylate: a randomized, double-blind, multicenter, placebo-controlled, parallel-group study. *BMC psychiatry*, *13*, 1-11.
9. Adler, L. A., Faraone, S. V., Spencer, T. J., Michelson, D., Reimherr, F. W., Glatt, S. J., ... & Biederman, J. (2008). The reliability and validity of self-and investigator ratings of ADHD in adults. *Journal of Attention Disorders*, *11*(6), 711-719.
10. Adler, L. A., Frick, G., & Yan, B. (2020). A long-term, open-label, safety study of triple-bead mixed amphetamine salts (SHP465) in adults with ADHD. *Journal of Attention Disorders*, *24*(3), 434-446.
11. Adler, L. A., Goodman, D. W., Kollins, S. H., Weisler, R. H., Krishnan, S., Zhang, Y., & Biederman, J. (2008). Double-blind, placebo-controlled study of the efficacy and safety of lisdexamfetamine dimesylate in adults with attention-deficit/hyperactivity disorder. *Journal of Clinical Psychiatry*, *69*(9), 1364.
12. Adler, L. A., Leon, T. L., Sardoff, T. M., Krone, B., Faraone, S. V., Silverstein, M. J., & Newcorn, J. H. (2021). A placebo-controlled trial of lisdexamfetamine in the treatment of comorbid sluggish cognitive tempo and adult ADHD. *The Journal of Clinical Psychiatry*, *82*(4), 34965.
13. Adler, L. A., Liebowitz, M., Kronenberger, W., Qiao, M., Rubin, R., Hollandbeck, M., ... & Durell, T. (2009). Atomoxetine treatment in adults with attention‐deficit/hyperactivity disorder and comorbid social anxiety disorder. *Depression and Anxiety*, *26*(3), 212-221.
14. Adler, L. A., Lynch, L. R., Shaw, D. M., Wallace, S. P., Ciranni, M. A., Briggie, A. M., ... & Faraone, S. V. (2011). Medication adherence and symptom reduction in adults treated with mixed amphetamine salts in a randomized crossover study. *Postgraduate medicine*, *123*(5), 71-79.
15. Adler, L. A., Lynch, L. R., Shaw, D. M., Wallace, S. P., O’Donnell, K. E., Ciranni, M. A., ... & Faraone, S. V. (2017). Effectiveness and duration of effect of open-label lisdexamfetamine dimesylate in adults with ADHD. *Journal of Attention Disorders*, *21*(2), 149-157.
16. Adler, L. A., Orman, C., Starr, H. L., Silber, S., Palumbo, J., Cooper, K., ... & Harrison, D. D. (2011). Long-term safety of OROS methylphenidate in adults with attention-deficit/hyperactivity disorder: an open-label, dose-titration, 1-year study. *Journal of clinical psychopharmacology*, *31*(1), 108-114.
17. Adler, L. A., Solanto, M., Escobar, R., Lipsius, S., & Upadhyaya, H. (2020). Executive functioning outcomes over 6 months of atomoxetine for adults with ADHD: relationship to maintenance of response and relapse over the subsequent 6 months after treatment. *Journal of Attention Disorders*, *24*(3), 363-372.
18. Adler, L. A., Spencer, T. J., Levine, L. R., Ramsey, J. L., Tamura, R., Kelsey, D., ... & Biederman, J. (2008). Functional outcomes in the treatment of adults with ADHD. *Journal of Attention Disorders*, *11*(6), 720-727.
19. Adler, L. A., Spencer, T. J., Milton, D. R., Moore, R. J., & Michelson, D. (2005). Long-term, open-label study of the safety and efficacy of atomoxetine in adults with attention-deficit/hyperactivity disorder: an interim analysis. *Journal of Clinical Psychiatry*, *66*(3), 294-299.
20. Adler, L. A., Spencer, T., Brown, T. E., Holdnack, J., Saylor, K., Schuh, K., ... & Kelsey, D. (2009). Once-daily atomoxetine for adult attention-deficit/hyperactivity disorder: a 6-month, double-blind trial. *Journal of Clinical Psychopharmacology*, *29*(1), 44-50.
21. Adler, L. A., Spencer, T., McGough, J. J., Jiang, H., & Muniz, R. (2009). Long-term effectiveness and safety of dexmethylphenidate extended-release capsules in adult ADHD. *Journal of Attention Disorders*, *12*(5), 449-459.
22. Adler, L. A., Sutton, V. K., Moore, R. J., Dietrich, A. P., Reimherr, F. W., Sangal, R. B., ... & Allen, A. J. (2006). Quality of life assessment in adult patients with attention-deficit/hyperactivity disorder treated with atomoxetine. *Journal of clinical psychopharmacology*, *26*(6), 648-652.
23. Adler, L. A., Weisler, R. H., Goodman, D. W., Hamdani, M., & Niebler, G. E. (2009). Short-term effects of lisdexamfetamine dimesylate on cardiovascular parameters in a 4-week clinical trial in adults with attention-deficit/hyperactivity disorder. *Journal of Clinical Psychiatry*, *16*(12), 1652.
24. Adler, L. A., Zimmerman, B., Starr, H. L., Silber, S., Palumbo, J., Orman, C., & Spencer, T. (2009). Efficacy and safety of OROS methylphenidate in adults with attention-deficit/hyperactivity disorder: a randomized, placebo-controlled, double-blind, parallel group, dose-escalation study. *Journal of clinical psychopharmacology*, *29*(3), 239-247.
25. Adler, L., Dietrich, A., Reimherr, F. W., Taylor, L. V., Sutton, V. K., Bakken, R., ... & Kelsey, D. (2006). Safety and tolerability of once versus twice daily atomoxetine in adults with ADHD. *Annals of Clinical Psychiatry*, *18*(2), 107-113.
26. Adler, L., Tanaka, Y., Williams, D., Trzepacz, P. T., Goto, T., Allen, A. J., ... & Upadhyaya, H. P. (2014). Executive function in adults with attention-deficit/hyperactivity disorder during treatment with atomoxetine in a randomized, placebo-controlled, withdrawal study. *Journal of clinical psychopharmacology*, *34*(4), 461-466.
27. Agay, N., Yechiam, E., Carmel, Z., & Levkovitz, Y. (2010). Non-specific effects of methylphenidate (Ritalin) on cognitive ability and decision-making of ADHD and healthy adults. *Psychopharmacology*, *210*, 511-519.
28. Aharonovich, E., Garawi, F., Bisaga, A., Brooks, D., Raby, W. N., Rubin, E., ... & Levin, F. R. (2006). Concurrent cannabis use during treatment for comorbid ADHD and cocaine dependence: effects on outcome. *The American journal of drug and alcohol abuse*, *32*(4), 629-635.
29. Ahlers, J., Baumgartner, C., Augsburger, M., Wenger, A., Malischnig, D., Boumparis, N., ... & Schaub, M. P. (2022). Cannabis use in adults who screen positive for attention deficit/hyperactivity disorder: CANreduce 2.0 Randomized Controlled Trial Subgroup Analysis. *Journal of Medical Internet Research*, *24*(4), e30138.
30. Alyagon, U., Shahar, H., Hadar, A., Barnea-Ygael, N., Lazarovits, A., Shalev, H., & Zangen, A. (2020). Alleviation of ADHD symptoms by non-invasive right prefrontal stimulation is correlated with EEG activity. *NeuroImage: Clinical*, *26*, 102206.
31. Amiri, S., Farhang, S., Ghoreishizadeh, M. A., Malek, A., & Mohammadzadeh, S. (2012). Double‐blind controlled trial of venlafaxine for treatment of adults with attention deficit/hyperactivity disorder. *Human Psychopharmacology: Clinical and Experimental*, *27*(1), 76-81.
32. Apostol, G., Abi-Saab, W., Kratochvil, C. J., Adler, L. A., Robieson, W. Z., Gault, L. M., ... & Saltarelli, M. D. (2012). Efficacy and safety of the novel α 4 β 2 neuronal nicotinic receptor partial agonist ABT-089 in adults with attention-deficit/hyperactivity disorder: a randomized, double-blind, placebo-controlled crossover study. *Psychopharmacology*, *219*, 715-725.
33. Arteaga-Henríquez, G., Ramos-Sayalero, C., Ibañez-Jimenez, P., Rosales-Ortiz, S. K., Kilencz, T., Schiweck, C., ... & Ramos-Quiroga, J. A. (2024). Efficacy of a synbiotic in the management of adults with Attention-Deficit and Hyperactivity Disorder and/or Borderline Personality Disorder, and high levels of irritability: Results from a multicenter, randomized, placebo-controlled,“basket” trial. *Brain, Behavior, and Immunity*.
34. Babinski, D. E., Waxmonsky, J. G., & Pelham, W. E. (2014). Treating parents with attention-deficit/hyperactivity disorder: The effects of behavioral parent training and acute stimulant medication treatment on parent–child interactions. *Journal of Abnormal Child Psychology*, *42*, 1129-1140.
35. Babinski, D. E., Waxmonsky, J. G., Waschbusch, D. A., Humphery, H., & Pelham Jr, W. E. (2017). Parent-reported improvements in family functioning in a randomized controlled trial of lisdexamfetamine for treatment of parental attention-deficit/hyperactivity disorder. *Journal of Child and Adolescent Psychopharmacology*, *27*(3), 250-257. Dubreuil-Vall, L., Gomez-Bernal, F., Villegas, A. C., Cirillo, P., Surman, C., Ruffini, G., ... & Camprodon, J. A. (2021). Transcranial direct current stimulation to the left dorsolateral prefrontal cortex improves cognitive control in patients with attention-deficit/hyperactivity disorder: a randomized behavioral and neurophysiological study. *Biological Psychiatry: Cognitive Neuroscience and Neuroimaging*, *6*(4), 439-448.
36. Bachmann, K., Lam, A. P., Sörös, P., Kanat, M., Hoxhaj, E., Matthies, S., ... & Philipsen, A. (2018). Effects of mindfulness and psychoeducation on working memory in adult ADHD: A randomised, controlled fMRI study. *Behaviour research and therapy*, *106*, 47-56.
37. Bain, E. E., Apostol, G., Sangal, R. B., Robieson, W. Z., McNeill, D. L., Abi-Saab, W. M., & Saltarelli, M. D. (2012). A randomized pilot study of the efficacy and safety of ABT-089, a novel α4β2 neuronal nicotinic receptor agonist, in adults with attention-deficit/hyperactivity disorder. *The Journal of clinical psychiatry*, *73*(6), 16813.
38. Barham, H., Büyükgök, D., Aksu, S., Soyata, A. Z., Bulut, G., Eskicioğlu, G., & Kulaksızoğlu, I. B. (2022). Evidence for modulation of planning and working memory capacities by transcranial direct current stimulation in a sample of adults with attention deficit hyperactivity disorder. *Neuroscience Letters*, *790*, 136883.
39. Barkley, R. A., Anderson, D. L., & Kruesi, M. (2007). A pilot study of the effects of atomoxetine on driving performance in adults with ADHD. *Journal of attention disorders*, *10*(3), 306-316.
40. Barkley, R. A., Murphy, K. R., O'Connell, T., & Connor, D. F. (2005). Effects of two doses of methylphenidate on simulator driving performance in adults with attention deficit hyperactivity disorder. *Journal of safety research*, *36*(2), 121-131.
41. Barth, B., Mayer-Carius, K., Strehl, U., Wyckoff, S. N., Haeussinger, F. B., Fallgatter, A. J., & Ehlis, A. C. (2021). A randomized-controlled neurofeedback trial in adult attention-deficit/hyperactivity disorder. *Scientific Reports*, *11*(1), 16873.
42. Ben-Sheetrit, J., Peskin, M., Newcorn, J. H., Daniely, Y., Shbiro, L., Rotem, A., ... & Manor, I. (2020). Characterizing the placebo response in adults with ADHD. *Journal of Attention Disorders*, *24*(3), 425-433.
43. Berlin, I., Hu, M. C., Covey, L. S., & Winhusen, T. (2012). Attention-deficit/hyperactivity disorder (ADHD) symptoms, craving to smoke, and tobacco withdrawal symptoms in adult smokers with ADHD. *Drug and alcohol dependence*, *124*(3), 268-273.
44. Biederman, J., Fried, R., Hammerness, P., Surman, C., Mehler, B., Petty, C. R., ... & Reimer, B. (2012). The effects of lisdexamfetamine dimesylate on the driving performance of young adults with ADHD: a randomized, double-blind, placebo-controlled study using a validated driving simulator paradigm. *Journal of psychiatric research*, *46*(4), 484-491.
45. Biederman, J., Fried, R., Tarko, L., Surman, C., Spencer, T., Pope, A., ... & Faraone, S. V. (2017). Memantine in the treatment of executive function deficits in adults with ADHD: A pilot-randomized double-blind controlled clinical trial. *Journal of Attention Disorders*, *21*(4), 343-352.
46. Biederman, J., Mick, E., Faraone, S., Hammerness, P., Surman, C., Harpold, T., ... & Spencer, T. (2006). A double-blind comparison of galantamine hydrogen bromide and placebo in adults with attention-deficit/hyperactivity disorder: a pilot study. *Journal of clinical psychopharmacology*, *26*(2), 163-166.
47. Biederman, J., Mick, E., Fried, R., Wilner, N., Spencer, T. J., & Faraone, S. V. (2011). Are stimulants effective in the treatment of executive function deficits? Results from a randomized double blind study of OROS-methylphenidate in adults with ADHD. *European Neuropsychopharmacology*, *21*(7), 508-515.
48. Biederman, J., Mick, E., Spencer, T., Surman, C., & Faraone, S. V. (2012). Is response to OROS‐methylphenidate treatment moderated by treatment with antidepressants or psychiatric comorbidity? A secondary analysis from a large randomized double blind study of adults with ADHD. *CNS neuroscience & therapeutics*, *18*(2), 126-132.
49. Biederman, J., Mick, E., Surman, C., Doyle, R., Hammerness, P., Harpold, T., ... & Spencer, T. (2006). A randomized, placebo-controlled trial of OROS methylphenidate in adults with attention-deficit/hyperactivity disorder. *Biological psychiatry*, *59*(9), 829-835.
50. Biederman, J., Mick, E., Surman, C., Doyle, R., Hammerness, P., Kotarski, M., & Spencer, T. (2010). A randomized, 3-phase, 34-week, double-blind, long-term efficacy study of osmotic-release oral system-methylphenidate in adults with attention-deficit/hyperactivity disorder. *Journal of clinical psychopharmacology*, *30*(5), 549-553.
51. Biederman, J., Spencer, T. J., Wilens, T. E., Weisler, R. H., Read, S. C., & Tulloch, S. J. (2005). Long-term safety and effectiveness of mixed amphetamine salts extended release in adults with ADHD. *CNS spectrums*, *10*(S20), 16-25.
52. Bleich-Cohen, M., Gurevitch, G., Carmi, N., Medvedovsky, M., Bregman, N., Nevler, N., ... & Ash, E. L. (2021). A functional magnetic resonance imaging investigation of prefrontal cortex deep transcranial magnetic stimulation efficacy in adults with attention deficit/hyperactive disorder: A double blind, randomized clinical trial. *NeuroImage: Clinical*, *30*, 102670.
53. Bloch, Y., Harel, E. V., Aviram, S., Govezensky, J., Ratzoni, G., & Levkovitz, Y. (2010). Positive effects of repetitive transcranial magnetic stimulation on attention in ADHD Subjects: a randomized controlled pilot study. *The World Journal of Biological Psychiatry*, *11*(5), 755-758.
54. Boonstra, A. M., Kooij, J. S., Oosterlaan, J., Sergeant, J. A., & Buitelaar, J. K. (2005). Does methylphenidate improve inhibition and other cognitive abilities in adults with childhood-onset ADHD?. *Journal of clinical and experimental neuropsychology*, *27*(3), 278-298.
55. Boonstra, A. M., Kooij, J. S., Oosterlaan, J., Sergeant, J. A., Buitelaar, J. K., & Van Someren, E. J. (2007). Hyperactive night and day? Actigraphy studies in adult ADHD: a baseline comparison and the effect of methylphenidate. *Sleep*, *30*(4), 433-442.
56. Bouffard, R., Hechtman, L., Minde, K., & Iaboni-Kassab, F. (2003). The efficacy of 2 different dosages of methylphenidate in treating adults with attention-deficit hyperactivity disorder. *The Canadian Journal of Psychiatry*, *48*(8), 546-554.
57. Brams, M., Giblin, J., Gasior, M., Gao, J., & Wigal, T. (2011). Effects of open-label lisdexamfetamine dimesylate on self-reported quality of life in adults with ADHD. *Postgraduate medicine*, *123*(3), 99-108.
58. Bron, T. I., Bijlenga, D., Boonstra, A. M., Breuk, M., Pardoen, W. F., Beekman, A. T., & Kooij, J. S. (2014). OROS-methylphenidate efficacy on specific executive functioning deficits in adults with ADHD: a randomized, placebo-controlled cross-over study. *European Neuropsychopharmacology*, *24*(4), 519-528.
59. Brown, T. E., Brams, M., Gao, J., Gasior, M., & Childress, A. (2010). Open-label administration of lisdexamfetamine dimesylate improves executive function impairments and symptoms of attention-deficit/hyperactivity disorder in adults. *Postgraduate medicine*, *122*(5), 7-17.
60. Brown, T. E., Chen, J., & Robertson, B. (2020). Relationships between executive function improvement and ADHD symptom improvement with lisdexamfetamine dimesylate in adults with ADHD and executive function deficits: a post hoc analysis. *The Primary Care Companion for CNS Disorders*, *22*(3), 23025.
61. Brown, T. E., Chen, J., & Robertson, B. (2022). Improved executive function in adults diagnosed with attention-deficit/hyperactivity disorder as measured by the brown attention-deficit disorder scale following treatment with SHP465 mixed amphetamine salts extended-release: post hoc analyses from 2 randomized, placebo-controlled studies. *Journal of Attention Disorders*, *26*(2), 256-266.
62. Brown, T. E., Holdnack, J., Saylor, K., Adler, L., Spencer, T., Williams, D. W., ... & Kelsey, D. (2011). Effect of atomoxetine on executive function impairments in adults with ADHD. *Journal of attention disorders*, *15*(2), 130-138.
63. Buitelaar, J. K., Casas, M., Philipsen, A., Kooij, J. J. S., Ramos-Quiroga, J. A., Dejonckheere, J., ... & Schäuble, B. (2012). Functional improvement and correlations with symptomatic improvement in adults with attention deficit hyperactivity disorder receiving long-acting methylphenidate. *Psychological Medicine*, *42*(1), 195-204.
64. Buitelaar, J. K., Kooij, J. S., Ramos-Quiroga, J. A., Dejonckheere, J., Casas, M., van Oene, J. C., ... & Trott, G. E. (2011). Predictors of treatment outcome in adults with ADHD treated with OROS® methylphenidate. *Progress in Neuro-Psychopharmacology and Biological Psychiatry*, *35*(2), 554-560.
65. Buitelaar, J. K., Trott, G. E., Hofecker, M., Waechter, S., Berwaerts, J., Dejonkheere, J., & Schäuble, B. (2012). Long-term efficacy and safety outcomes with OROS-MPH in adults with ADHD. *International Journal of Neuropsychopharmacology*, *15*(1), 1-13.
66. Bush, G., Spencer, T. J., Holmes, J., Shin, L. M., Valera, E. M., Seidman, L. J., ... & Biederman, J. (2008). Functional magnetic resonance imaging of methylphenidate and placebo in attention-deficit/hyperactivity disorder during the multi-source interference task. *Archives of general psychiatry*, *65*(1), 102-114.
67. Butterfield, M. E., Saal, J., Young, B., & Young, J. L. (2016). Supplementary guanfacine hydrochloride as a treatment of attention deficit hyperactivity disorder in adults: a double blind, placebo-controlled study. *Psychiatry research*, *236*, 136-141.
68. Cachoeira, C. T., Leffa, D. T., Mittelstadt, S. D., Mendes, L. S. T., Brunoni, A. R., Pinto, J. V., ... & Schestatsky, P. (2017). Positive effects of transcranial direct current stimulation in adult patients with attention-deficit/hyperactivity disorder A pilot randomized controlled study. *Psychiatry Research*, *247*, 28-32.
69. Carpentier, P. J., De Jong, C. A., Dijkstra, B. A., Verbrugge, C. A., & Krabbe, P. F. (2005). A controlled trial of methylphenidate in adults with attention deficit/hyperactivity disorder and substance use disorders. *Addiction*, *100*(12), 1868-1874.
70. Carvalho, L. R., Haas, L. M., Zeni, G., Victor, M. M., Techele, S. P., Castanho, J. M., ... & Rohde, L. A. (2023). Evaluation of the effectiveness of the FOCUS ADHD App in monitoring adults with attention-deficit/hyperactivity disorder. *European Psychiatry*, *66*(1), e53.
71. Chamberlain, S. R., Del Campo, N., Dowson, J., Müller, U., Clark, L., Robbins, T. W., & Sahakian, B. J. (2007). Atomoxetine improved response inhibition in adults with attention deficit/hyperactivity disorder. *Biological psychiatry*, *62*(9), 977-984.
72. Cherkasova, M. V., French, L. R., Syer, C. A., Cousins, L., Galina, H., Ahmadi-Kashani, Y., & Hechtman, L. (2020). Efficacy of cognitive behavioral therapy with and without medication for adults with ADHD: A randomized clinical trial. *Journal of attention disorders*, *24*(6), 889-903.
73. Childress, A., Cutler, A. J., Adler, L. A., Fry, N., Asubonteng, K., Maldonado-Cruz, Z., ... & Rubin, J. (2024). An Open-Label Extension Study Assessing the Long-Term Safety and Efficacy of Viloxazine Extended-Release Capsules in Adults with Attention-Deficit/Hyperactivity Disorder. *CNS drugs*, 1-17.
74. Childress, A., Cutler, A. J., Marraffino, A. H., Bhaskar, S., & Donnelly, G. (2022). Randomized, double-blind, placebo-controlled, parallel-group, adult laboratory classroom study of the efficacy and safety of PRC-063 (extended-release methylphenidate) for the treatment of ADHD. *Journal of Attention Disorders*, *26*(6), 857-869.
75. Chronis-Tuscano, A., Wang, C. H., Strickland, J., Almirall, D., & Stein, M. A. (2016). Personalized treatment of mothers with ADHD and their young at-risk children: A SMART pilot. *Journal of Clinical Child & Adolescent Psychology*, *45*(4), 510-521.
76. Conzelmann, A., Woidich, E., Mucha, R. F., Weyers, P., Müller, M., Lesch, K. P., ... & Pauli, P. (2016). Methylphenidate and emotional-motivational processing in attention-deficit/hyperactivity disorder. *Journal of Neural Transmission*, *123*, 971-979.
77. Cooper, R. E., Williams, E., Seegobin, S., Tye, C., Kuntsi, J., & Asherson, P. (2017). Cannabinoids in attention-deficit/hyperactivity disorder: A randomised-controlled trial. *European Neuropsychopharmacology*, *27*(8), 795-808.
78. Corrales, M., García-González, S., Richarte, V., Fadeuilhe, C., Daigre, C., García-Gea, E., & Ramos-Quiroga, J. A. (2024). Long-term efficacy of a new 6-session cognitive behavioral therapy for adults with attention-deficit/hyperactivity disorder: A randomized, controlled clinical trial. *Psychiatry Research*, *331*, 115642.
79. Cosmo, C., Baptista, A. F., de Araújo, A. N., do Rosário, R. S., Miranda, J. G. V., Montoya, P., & de Sena, E. P. (2015). A randomized, double-blind, sham-controlled trial of transcranial direct current stimulation in attention-deficit/hyperactivity disorder. *PloS one*, *10*(8), e0135371.
80. Covey, L. S., Hu, M. C., Green, C. A., Brigham, G., Hurt, R. D., Adler, L., & Winhusen, T. (2011). An exploration of site effects in a multisite trial of OROS-methylphenidate for smokers with attention deficit/hyperactivity disorder. *The American journal of drug and alcohol abuse*, *37*(5), 392-399.
81. Covey, L. S., Hu, M. C., Weissman, J., Croghan, I., Adler, L., & Winhusen, T. (2011). Divergence by ADHD subtype in smoking cessation response to OROS-methylphenidate. *Nicotine & Tobacco Research*, *13*(10), 1003-1008.
82. Covey, L. S., Hu, M. C., Winhusen, T., Weissman, J., Berlin, I., & Nunes, E. V. (2010). OROS-methylphenidate or placebo for adult smokers with attention deficit hyperactivity disorder: racial/ethnic differences. *Drug and alcohol dependence*, *110*(1-2), 156-159.
83. Cox, D. J., Davis, M., Mikami, A. Y., Singh, H., Merkel, R. L., & Burket, R. (2012). Long-acting methylphenidate reduces collision rates of young adult drivers with attention-deficit/hyperactivity disorder. *Journal of clinical psychopharmacology*, *32*(2), 225-230.
84. Cox, D. J., Merkel, R. L., Kovatchev, B., & Seward, R. (2000). Effect of stimulant medication on driving performance of young adults with attention-deficit hyperactivity disorder: a preliminary double-blind placebo controlled trial. *The Journal of nervous and mental disease*, *188*(4), 230-234.
85. Cutler, A. J., Childress, A. C., Pardo, A., Duhoux, S., Gomeni, R., Rafla, E., ... & Kando, J. C. (2022). Randomized, double-blind, placebo-controlled, fixed-dose study to evaluate the efficacy and safety of amphetamine extended-release tablets in adults with attention-deficit/hyperactivity disorder. *The Journal of Clinical Psychiatry*, *83*(5), 41875.
86. da Silva, P. H. R., Leffa, D. T., Luethi, M. S., Silva, R. F., Ferrazza, C. P., Picon, F. A., ... & Brunoni, A. R. (2024). Baseline brain volume predicts home-based transcranial direct current stimulation effects on inattention in adults with attention-deficit/hyperactivity disorder. *Journal of Psychiatric Research*, *177*, 403-411.
87. del Campo, N., Fryer, T. D., Hong, Y. T., Smith, R., Brichard, L., Acosta-Cabronero, J., ... & Müller, U. (2013). A positron emission tomography study of nigro-striatal dopaminergic mechanisms underlying attention: implications for ADHD and its treatment. *Brain*, *136*(11), 3252-3270.
88. Dittner, A. J., Hodsoll, J., Rimes, K. A., Russell, A. J., & Chalder, T. (2018). Cognitive–behavioural therapy for adult attention‐deficit hyperactivity disorder: A proof of concept randomised controlled trial. *Acta Psychiatrica Scandinavica*, *137*(2), 125-137.
89. Dorrego, M. F., Canevaro, L., Kuzis, G., Sabe, L., & Starkstein, S. E. (2002). A randomized, double-blind, crossover study of methylphenidate and lithium in adults with attention-deficit/hyperactivity disorder: preliminary findings. *The Journal of neuropsychiatry and clinical neurosciences*, *14*(3), 289-295.
90. DuPaul, G. J., Weyandt, L. L., Rossi, J. S., Vilardo, B. A., O’Dell, S. M., Carson, K. M., ... & Swentosky, A. (2012). Double-blind, placebo-controlled, crossover study of the efficacy and safety of lisdexamfetamine dimesylate in college students with ADHD. *Journal of attention disorders*, *16*(3), 202-220.
91. Eddy, L. D., Anastopoulos, A. D., Dvorsky, M. R., Silvia, P. J., Labban, J. D., & Langberg, J. M. (2021). An RCT of a CBT intervention for emerging adults with ADHD attending college: functional outcomes. *Journal of Clinical Child & Adolescent Psychology*, *50*(6), 844-857.
92. Emilsson, B., Gudjonsson, G., Sigurdsson, J. F., Baldursson, G., Einarsson, E., Olafsdottir, H., & Young, S. (2011). Cognitive behaviour therapy in medication-treated adults with ADHD and persistent symptoms: a randomized controlled trial. *BMC psychiatry*, *11*, 1-10.
93. Ernst, M., Liebenauer, L. L., Tebeka, D., Jons, P. H., Eisenhofer, G., Murphy, D. L., & Zametkin, A. J. (1997). Selegiline in ADHD adults, plasma monoamines and monoamine metabolites. *Neuropsychopharmacology*, *16*(4), 276-284.
94. Fan, L. Y., Chou, T. L., & Gau, S. S. F. (2017). Neural correlates of atomoxetine improving inhibitory control and visual processing in Drug‐naïve adults with attention‐deficit/hyperactivity disorder. *Human Brain Mapping*, *38*(10), 4850-4864.
95. Faraone, S. V., Biederman, J., Spencer, T., Michelson, D., Adler, L., Reimherr, F., & Seidman, L. (2005). Atomoxetine and stroop task performance in adult attention-deficit/hyperactivity disorder. *Journal of Child & Adolescent Psychopharmacology*, *15*(4), 664-670.
96. Faraone, S. V., Childress, A., Caras, S., Arnold, V. K., Montano, C. B., Sarkis, E. H., ... & Young, J. L. (2021). A randomized, double-blind, placebo-controlled trial to evaluate the efficacy and safety of AR19, a manipulation-resistant formulation of amphetamine sulfate, in adults with attention-deficit/hyperactivity disorder. *The Journal of Clinical Psychiatry*, *82*(5), 36309.
97. Faraone, S. V., Gomeni, R., Hull, J. T., Chaturvedi, S. A., Busse, G. D., Melyan, Z., ... & Nasser, A. (2022). Predicting efficacy of viloxazine extended-release treatment in adults with ADHD using an early change in ADHD symptoms: machine learning post hoc analysis of a phase 3 clinical trial. *Psychiatry Research*, *318*, 114922.
98. Faraone, S. V., Spencer, T. J., Kollins, S. H., Glatt, S. J., & Goodman, D. (2012). Dose response effects of lisdexamfetamine dimesylate treatment in adults with ADHD: an exploratory study. *Journal of attention disorders*, *16*(2), 118-127.
99. Fleming, A. P., McMahon, R. J., Moran, L. R., Peterson, A. P., & Dreessen, A. (2015). Pilot randomized controlled trial of dialectical behavior therapy group skills training for ADHD among college students. *Journal of attention disorders*, *19*(3), 260-271.
100. Fredericks, E. M., & Kollins, S. H. (2004). Assessing methylphenidate preference in ADHD patients using a choice procedure. *Psychopharmacology*, *175*, 391-398.
101. Frick, G., Yan, B., & Adler, L. A. (2020). Triple-bead mixed amphetamine salts (SHP465) in adults with ADHD: results of a phase 3, double-blind, randomized, forced-dose trial. *Journal of Attention Disorders*, *24*(3), 402-413.
102. Gabriel, A., & Violato, C. (2011). Adjunctive atomoxetine to SSRIs or SNRIs in the treatment of adult ADHD patients with comorbid partially responsive generalized anxiety (GA): an open-label study. *ADHD Attention Deficit and Hyperactivity Disorders*, *3*, 319-326.
103. Gehricke, J. G., Whalen, C. K., Jamner, L. D., Wigal, T. L., & Steinhoff, K. (2006). The reinforcing effects of nicotine and stimulant medication in the everyday lives of adult smokers with ADHD: A preliminary examination. *Nicotine & tobacco research*, *8*(1), 37-47.
104. Gift, T. E., Reimherr, F. W., Marchant, B. K., Steans, T. A., & Wender, P. H. (2016). Personality disorder in adult attention-deficit/hyperactivity disorder: attrition and change during long-term treatment. *The Journal of Nervous and Mental Disease*, *204*(5), 355-363.
105. Ginsberg, L., Katic, A., Adeyi, B., Dirks, B., Babcock, T., Lasser, R., ... & Adler, L. A. (2011). Long-term treatment outcomes with lisdexamfetamine dimesylate for adults with attention-deficit/hyperactivity disorder stratified by baseline severity. *Current medical research and opinion*, *27*(6), 1097-1107.
106. Ginsberg, Y., & Lindefors, N. (2012). Methylphenidate treatment of adult male prison inmates with attention-deficit hyperactivity disorder: randomised double-blind placebo-controlled trial with open-label extension. *The British Journal of Psychiatry*, *200*(1), 68-73.
107. Ginsberg, Y., Arngrim, T., Philipsen, A., Gandhi, P., Chen, C. W., Kumar, V., & Huss, M. (2014). Long-term (1 year) safety and efficacy of methylphenidate modified-release long-acting formulation (MPH-LA) in adults with attention-deficit hyperactivity disorder: a 26-week, flexible-dose, open-label extension to a 40-week, double-blind, randomised, placebo-controlled core study. *CNS drugs*, *28*, 951-962.
108. Ginsberg, Y., Hirvikoski, T., & Lindefors, N. (2010). Attention deficit hyperactivity disorder (ADHD) among longer-term prison inmates is a prevalent, persistent and disabling disorder. *BMC psychiatry*, *10*, 1-13.
109. Ginsberg, Y., Hirvikoski, T., Grann, M., & Lindefors, N. (2012). Long-term functional outcome in adult prison inmates with ADHD receiving OROS-methylphenidate. *European Archives of Psychiatry and Clinical Neuroscience*, *262*, 705-724.
110. Goodman, D. W., Ginsberg, L., Weisler, R. H., Cutler, A. J., & Hodgkins, P. (2005). An interim analysis of the Quality of Life, Effectiveness, Safety, and Tolerability (QU. EST) evaluation of mixed amphetamine salts extended release in adults with ADHD. *Cns Spectrums*, *10*(S20), 26-34.
111. Goodman, D. W., Starr, H. L., Ma, Y. W., Rostain, A. L., Ascher, S., & Armstrong, R. B. (2017). Randomized, 6-week, placebo-controlled study of treatment for adult attention-deficit/hyperactivity disorder: individualized dosing of osmotic-release oral system (OROS) methylphenidate with a goal of symptom remission. *The Journal of Clinical Psychiatry*, *78*(1), 9021.
112. Grinblat, N., & Rosenblum, S. (2023). Work-MAP telehealth metacognitive work-performance intervention for adults with ADHD: Randomized controlled trial. *OTJR: Occupational Therapy Journal of Research*, *43*(3), 435-445.
113. Gu, Y., Xu, G., & Zhu, Y. (2018). A randomized controlled trial of mindfulness-based cognitive therapy for college students with ADHD. *Journal of attention disorders*, *22*(4), 388-399.
114. Gutman, S. A., Balasubramanian, S., Herzog, M., Kim, E., Swirnow, H., Retig, Y., & Wolff, S. (2020). Effectiveness of a tailored intervention for women with attention deficit hyperactivity disorder (ADHD) and ADHD symptoms: a randomized controlled study. *The American Journal of Occupational Therapy*, *74*(1), 7401205010p1-7401205010p11.
115. Halmøy, A., Ring, A. E., Gjestad, R., Møller, M., Ubostad, B., Lien, T., ... & Fredriksen, M. (2022). Dialectical behavioral therapy-based group treatment versus treatment as usual for adults with attention-deficit hyperactivity disorder: a multicenter randomized controlled trial. *Bmc Psychiatry*, *22*(1), 738.
116. Hepark, S., Janssen, L., de Vries, A., Schoenberg, P. L., Donders, R., Kan, C. C., & Speckens, A. E. (2019). The efficacy of adapted MBCT on core symptoms and executive functioning in adults with ADHD: A preliminary randomized controlled trial. *Journal of Attention Disorders*, *23*(4), 351-362.
117. Hirata, Y., Goto, T., Takita, Y., Trzepacz, P. T., Allen, A. J., Ichikawa, H., & Takahashi, M. (2014). Long‐term safety and tolerability of atomoxetine in J apanese adults with attention deficit hyperactivity disorder. *Asia‐Pacific Psychiatry*, *6*(3), 292-301.
118. Hirvikoski, T., Lindström, T., Carlsson, J., Waaler, E., Jokinen, J., & Bölte, S. (2017). Psychoeducational groups for adults with ADHD and their significant others (PEGASUS): A pragmatic multicenter and randomized controlled trial. *European Psychiatry*, *44*, 141-152.
119. Hirvikoski, T., Waaler, E., Alfredsson, J., Pihlgren, C., Holmström, A., Johnson, A., ... & Nordström, A. L. (2011). Reduced ADHD symptoms in adults with ADHD after structured skills training group: results from a randomized controlled trial. *Behaviour research and therapy*, *49*(3), 175-185.
120. Hoxhaj, E., Sadohara, C., Borel, P., D’Amelio, R., Sobanski, E., Müller, H., ... & Philipsen, A. (2018). Mindfulness vs psychoeducation in adult ADHD: a randomized controlled trial. *European archives of psychiatry and clinical neuroscience*, *268*, 321-335.
121. Huang, F., Tang, Y. L., Zhao, M., Wang, Y., Pan, M., Wang, Y., & Qian, Q. (2019). Cognitive-behavioral therapy for adult ADHD: A randomized clinical trial in China. *Journal of Attention Disorders*, *23*(9), 1035-1046.
122. Hunt, M. G., Bienstock, S. W., & Qiang, J. K. (2012). Effects of diurnal variation on the Test of Variables of Attention performance in young adults with attention-deficit/hyperactivity disorder. *Psychological Assessment*, *24*(1), 166.
123. Huss, M., Ginsberg, Y., Arngrim, T., Philipsen, A., Carter, K., Chen, C. W., ... & Kumar, V. (2014). Open-label dose optimization of methylphenidate modified release long acting (MPH-LA): a post hoc analysis of real-life titration from a 40-week randomized trial. *Clinical Drug Investigation*, *34*, 639-649.
124. Huss, M., Ginsberg, Y., Tvedten, T., Arngrim, T., Philipsen, A., Carter, K., ... & Kumar, V. (2014). Methylphenidate hydrochloride modified-release in adults with attention deficit hyperactivity disorder: a randomized double-blind placebo-controlled trial. *Advances in therapy*, *31*, 44-65.
125. Iwanami, A., Saito, K., Fujiwara, M., Okutsu, D., & Ichikawa, H. (2020). Efficacy and safety of guanfacine extended-release in the treatment of attention-deficit/hyperactivity disorder in adults: results of a randomized, double-blind, placebo-controlled study. *The Journal of clinical psychiatry*, *81*(3), 7891.
126. Jain, U., Hechtman, L., Weiss, M., Ahmed, T. S., Reiz, J. L., Donnelly, G. A., ... & Darke, A. C. (2007). Efficacy of a novel biphasic controlled-release methylphenidate formula in adults with attention-deficit/hyperactivity disorder: results of a double-blind, placebo-controlled crossover study. *Journal of Clinical Psychiatry*, *68*(2), 268-277.
127. Jans, T., Jacob, C., Warnke, A., Zwanzger, U., Groß‐Lesch, S., Matthies, S., ... & Philipsen, A. (2015). Does intensive multimodal treatment for maternal ADHD improve the efficacy of parent training for children with ADHD? A randomized controlled multicenter trial. *Journal of Child Psychology and Psychiatry*, *56*(12), 1298-1313.
128. Janssen, L., Kan, C. C., Carpentier, P. J., Sizoo, B., Hepark, S., Grutters, J., ... & Speckens, A. E. (2015). Mindfulness based cognitive therapy versus treatment as usual in adults with attention deficit hyperactivity disorder (ADHD). *BMC psychiatry*, *15*, 1-10.
129. Jansson, L., Löhman, M., Östlund, M., & Domingo, B. (2023). Effects of one single-dose methylphenidate compared to one single-dose placebo on QbTest performance in adults with untreated ADHD: a randomized controlled trial. *BMC psychiatry*, *23*(1), 762.
130. Jucaite, A., Öhd, J., Potter, A. S., Jaeger, J., Karlsson, P., Hannesdottir, K., ... & Paulsson, B. (2014). A randomized, double-blind, placebo-controlled crossover study of α 4 β 2* nicotinic acetylcholine receptor agonist AZD1446 (TC-6683) in adults with attention-deficit/hyperactivity disorder. *Psychopharmacology*, *231*, 1251-1265.
131. Koblan, K. S., Hopkins, S. C., Sarma, K., Jin, F., Goldman, R., Kollins, S. H., & Loebel, A. (2015). Dasotraline for the treatment of attention-deficit/hyperactivity disorder: a randomized, double-blind, placebo-controlled, proof-of-concept trial in adults. *Neuropsychopharmacology*, *40*(12), 2745-2752.
132. Kollins, S. H., English, J. S., Itchon-Ramos, N., Chrisman, A. K., Dew, R., O’Brien, B., & McClernon, F. J. (2014). A pilot study of lis-dexamfetamine dimesylate (LDX/SPD489) to facilitate smoking cessation in nicotine-dependent adults with ADHD. *Journal of attention disorders*, *18*(2), 158-168.
133. Kollins, S. H., English, J., Robinson, R., Hallyburton, M., & Chrisman, A. K. (2009). Reinforcing and subjective effects of methylphenidate in adults with and without attention deficit hyperactivity disorder (ADHD). *Psychopharmacology*, *204*, 73-83.
134. Kollins, S. H., Schoenfelder, E., English, J. S., McClernon, F. J., Dew, R. E., & Lane, S. D. (2013). Methylphenidate does not influence smoking-reinforced responding or attentional performance in adult smokers with and without attention deficit hyperactivity disorder (ADHD). *Experimental and clinical psychopharmacology*, *21*(5), 375.
135. Konstenius, M., Jayaram-Lindström, N., Beck, O., & Franck, J. (2010). Sustained release methylphenidate for the treatment of ADHD in amphetamine abusers: a pilot study. *Drug and alcohol dependence*, *108*(1-2), 130-133.
136. Konstenius, M., Jayaram‐Lindström, N., Guterstam, J., Beck, O., Philips, B., & Franck, J. (2014). Methylphenidate for attention deficit hyperactivity disorder and drug relapse in criminal offenders with substance dependence: a 24‐week randomized placebo‐controlled trial. *Addiction*, *109*(3), 440-449.
137. Kooij, J. J. S., Burger, H., Boonstra, A. M., Van der Linden, P. D., Kalma, L. E., & Buitelaar, J. K. (2004). Efficacy and safety of methylphenidate in 45 adults with attention-deficit/hyperactivity disorder. A randomized placebo-controlled double-blind cross-over trial. *Psychological medicine*, *34*(6), 973-982.
138. Kooij, J. S., Boonstra, A. M., Vermeulen, S. H., Heister, A. G., Burger, H., Buitelaar, J. K., & Franke, B. (2008). Response to methylphenidate in adults with ADHD is associated with a polymorphism in SLC6A3 (DAT1). *American Journal of Medical Genetics Part B: Neuropsychiatric Genetics*, *147*(2), 201-208.
139. Krause, J., la Fougere, C., Krause, K. H., Ackenheil, M., & Dresel, S. H. (2005). Influence of striatal dopamine transporter availability on the response to methylphenidate in adult patients with ADHD. *European archives of psychiatry and clinical neuroscience*, *255*, 428-431.
140. Kuperman, S., Perry, P. J., Gaffney, G. R., Lund, B. C., Bever-Stille, K. A., Arndt, S., ... & Paulsen, J. S. (2001). Bupropion SR vs. methylphenidate vs. placebo for attention deficit hyperactivity disorder in adults. *Annals of Clinical Psychiatry*, *13*, 129-134.
141. Lam, A. P., Matthies, S., Graf, E., Colla, M., Jacob, C., Sobanski, E., ... & Philipsen, A. (2019). Long-term effects of multimodal treatment on adult attention-deficit/hyperactivity disorder symptoms: follow-up analysis of the COMPAS trial. *JAMA network open*, *2*(5), e194980-e194980.
142. Lee, S. I., Song, D. H., Shin, D. W., Kim, J. H., Lee, Y. S., Hwang, J. W., ... & Treuer, T. (2014). Efficacy and safety of atomoxetine hydrochloride in K orean adults with attention‐deficit hyperactivity disorder. *Asia‐Pacific Psychiatry*, *6*(4), 386-396.
143. Leffa, D. T., Grevet, E. H., Bau, C. H. D., Schneider, M., Ferrazza, C. P., Da Silva, R. F., ... & Rohde, L. A. (2022). Transcranial direct current stimulation vs sham for the treatment of inattention in adults with attention-deficit/hyperactivity disorder: the TUNED randomized clinical trial. *JAMA psychiatry*, *79*(9), 847-856.
144. Leuchter, A. F., McGough, J. J., Korb, A. S., Hunter, A. M., Glaser, P. E., Deldar, A., ... & Cook, I. A. (2014). Neurophysiologic predictors of response to atomoxetine in young adults with attention deficit hyperactivity disorder: a pilot project. *Journal of psychiatric research*, *54*, 11-18.
145. Levin, E. D., Conners, C. K., Sparrow, E., Hinton, S. C., Erhardt, D., Meck, W. H., ... & March, J. (1996). Nicotine effects on adults with attention-deficit/hyperactivity disorder. *Psychopharmacology*, *123*, 55-63.
146. Levin, F. R., Bisaga, A., Raby, W., Aharonovich, E., Rubin, E., Mariani, J., ... & Nunes, E. V. (2008). Effects of major depressive disorder and attention-deficit/hyperactivity disorder on the outcome of treatment for cocaine dependence. *Journal of substance abuse treatment*, *34*(1), 80-89.
147. Levin, F. R., Choi, C. J., Pavlicova, M., Mariani, J. J., Mahony, A., Brooks, D. J., ... & Grabowski, J. (2018). How treatment improvement in ADHD and cocaine dependence are related to one another: A secondary analysis. *Drug and alcohol dependence*, *188*, 135-140.
148. Levin, F. R., Evans, S. M., Brooks, D. J., & Garawi, F. (2007). Treatment of cocaine dependent treatment seekers with adult ADHD: double-blind comparison of methylphenidate and placebo. *Drug and alcohol dependence*, *87*(1), 20-29.
149. Levin, F. R., Evans, S. M., Brooks, D. J., Kalbag, A. S., Garawi, F., & Nunes, E. V. (2006). Treatment of methadone-maintained patients with adult ADHD: double-blind comparison of methylphenidate, bupropion and placebo. *Drug and Alcohol Dependence*, *81*(2), 137-148.
150. Levin, F. R., Evans, S. M., McDowell, D. M., Brooks, D. J., & Nunes, E. (2002). Bupropion treatment for cocaine abuse and adult attention-deficit/hyperactivity disorder. *Journal of Addictive Diseases*, *21*(2), 1-16.
151. Levin, F. R., Mariani, J. J., Pavlicova, M., Choi, C. J., Basaraba, C., Mahony, A. L., ... & Naqvi, N. (2024). Extended-Release Mixed Amphetamine Salts for Comorbid Adult Attention-Deficit/Hyperactivity Disorder and Cannabis Use Disorder: A Pilot, Randomized Double-Blind, Placebo-Controlled Trial. *Journal of Attention Disorders*, *28*(11), 1467-1481.
152. Levin, F. R., Mariani, J. J., Specker, S., Mooney, M., Mahony, A., Brooks, D. J., ... & Grabowski, J. (2015). Extended-release mixed amphetamine salts vs placebo for comorbid adult attention-deficit/hyperactivity disorder and cocaine use disorder: a randomized clinical trial. *JAMA psychiatry*, *72*(6), 593-602.
153. Levy Schwartz, M., Magzal, F., Yehuda, I., & Tamir, S. (2024). Exploring the impact of probiotics on adult ADHD management through a double-blind RCT. *Scientific Reports*, *14*(1), 26830.
154. Liu, Z. X., Glizer, D., Tannock, R., & Woltering, S. (2016). EEG alpha power during maintenance of information in working memory in adults with ADHD and its plasticity due to working memory training: A randomized controlled trial. *Clinical Neurophysiology*, *127*(2), 1307-1320.
155. Löhman, M., Domingo, B., Östlund, M., & Jansson, L. (2023). Contrasting expectancy effects with objective measures in adults with untreated ADHD during QbTest. *Scandinavian Journal of Psychology*, *64*(4), 461-469.
156. López-Pinar, C., Rosen, H., Selaskowski, B., Staerk, C., Jans, T., Retz, W., ... & Philipsen, A. (2024). Exploring the Relationship between Adherence to Therapy, Treatment Acceptability, and Clinical Outcomes in Adults with Attention-Deficit/Hyperactivity Disorder: Results from the COMPAS Multicenter Randomized Controlled Trial. *Psychotherapy and Psychosomatics*, *93*(1), 46-64.
157. López-Pinar, C., Selaskowski, B., Braun, N., Fornés-Ferrer, V., Euscher, R., Matthies, S., ... & Philipsen, A. (2023). Exploring the efficacy of dialectical behaviour therapy and methylphenidate on emotional comorbid symptoms in adults with attention Deficit/Hyperactivity disorder: Results of the COMPAS multicentre randomised controlled trial. *Psychiatry Research*, *330*, 115610.
158. Luo, S. X., Covey, L. S., Hu, M. C., Levin, F. R., Nunes, E. V., & Winhusen, T. M. (2015). Toward personalized smoking‐cessation treatment: Using a predictive modeling approach to guide decisions regarding stimulant medication treatment of attention‐deficit/hyperactivity disorder (ADHD) in smokers. *The American journal on addictions*, *24*(4), 348-356.
159. Luo, S. X., Wall, M., Covey, L., Hu, M. C., Scodes, J. M., Levin, F. R., ... & Winhusen, T. (2018). Exploring longitudinal course and treatment-baseline severity interactions in secondary outcomes of smoking cessation treatment in individuals with attention-deficit hyperactivity disorder. *The American journal of drug and alcohol abuse*, *44*(6), 653-659.
160. Madaan, V., Bhaskar, S., Donnelly, G. A., & Cox, D. J. (2024). A Randomized, Phase 3, Double-Blind, Crossover Comparison of Multilayer, Extended-Release Methylphenidate (PRC-063), and Lisdexamfetamine in the Driving Performance of Young Adults With ADHD. *Journal of Attention Disorders*, *28*(6), 947-956.
161. Maier, S., Perlov, E., Graf, E., Dieter, E., Sobanski, E., Rump, M., ... & van Elst, L. T. (2016). Discrete global but no focal gray matter volume reductions in unmedicated adult patients with attention-deficit/hyperactivity disorder. *Biological psychiatry*, *80*(12), 905-915.
162. Manor, I., Newcorn, J. H., Faraone, S. V., Adler, L. A., & Metadoxine Study Group. (2013). Efficacy of metadoxine extended release in patients with predominantly inattentive subtype attention-deficit/hyperactivity disorder. *Postgraduate Medicine*, *125*(4), 181-190.
163. Manor, I., Rubin, J., Daniely, Y., & Adler, L. A. (2014). Attention benefits after a single dose of metadoxine extended release in adults with predominantly inattentive ADHD. *Postgraduate Medicine*, *126*(5), 7-16.
164. Marchant, B. K., Reimherr, F. W., Halls, C., Williams, E. D., Strong, R. E., Kondo, D., ... & Robison, R. J. (2011). Long-term open-label response to atomoxetine in adult ADHD: influence of sex, emotional dysregulation, and double-blind response to atomoxetine. *ADHD Attention Deficit and Hyperactivity Disorders*, *3*, 237-244.
165. Marchant, B. K., Reimherr, F. W., Robison, R. J., Olsen, J. L., & Kondo, D. G. (2011). Methylphenidate transdermal system in adult ADHD and impact on emotional and oppositional symptoms. *Journal of attention disorders*, *15*(4), 295-304.
166. Martin, P. T., Corcoran, M., Zhang, P., & Katic, A. (2014). Randomized, double-blind, placebo-controlled, crossover study of the effects of lisdexamfetamine dimesylate and mixed amphetamine salts on cognition throughout the day in adults with attention-deficit/hyperactivity disorder. *Clinical drug investigation*, *34*, 147-157.
167. Martiny, K., Nielsen, N. P., & Wiig, E. H. (2020). Differentiating depression and ADHD without depression in adults with processing-speed measures. *Acta neuropsychiatrica*, *32*(5), 237-246.
168. Matza, L. S., Johnston, J. A., Faries, D. E., Malley, K. G., & Brod, M. (2007). Responsiveness of the adult attention-deficit/hyperactivity disorder quality of life scale (AAQoL). *Quality of Life Research*, *16*, 1511-1520.
169. Mawjee, K., Woltering, S., & Tannock, R. (2015). Working memory training in post-secondary students with ADHD: A randomized controlled study. *PloS one*, *10*(9), e0137173.
170. Mawjee, K., Woltering, S., Lai, N., Gotlieb, H., Kronitz, R., & Tannock, R. (2017). Working memory training in ADHD: controlling for engagement, motivation, and expectancy of improvement (pilot study). *Journal of Attention Disorders*, *21*(11), 956-968.
171. McClernon, F. J., Kollins, S. H., Lutz, A. M., Fitzgerald, D. P., Murray, D. W., Redman, C., & Rose, J. E. (2008). Effects of smoking abstinence on adult smokers with and without attention deficit hyperactivity disorder: results of a preliminary study. *Psychopharmacology*, *197*, 95-105.
172. McRae‐Clark, A. L., Carter, R. E., Killeen, T. K., Carpenter, M. J., White, K. G., & Brady, K. T. (2010). A placebo‐controlled trial of atomoxetine in marijuana‐dependent individuals with attention deficit hyperactivity disorder. *The American journal on addictions*, *19*(6), 481-489.
173. Medori, R., Ramos-Quiroga, J. A., Casas, M., Kooij, J. J. S., Niemelä, A., Trott, G. E., ... & Buitelaar, J. K. (2008). A randomized, placebo-controlled trial of three fixed dosages of prolonged-release OROS methylphenidate in adults with attention-deficit/hyperactivity disorder. *Biological psychiatry*, *63*(10), 981-989.
174. Meinzer, M. C., Oddo, L. E., Vasko, J. M., Murphy, J. G., Iwamoto, D., Lejuez, C. W., & Chronis-Tuscano, A. (2021). Motivational interviewing plus behavioral activation for alcohol misuse in college students with ADHD. *Psychology of addictive behaviors*, *35*(7), 803.
175. Michelson, D., Adler, L., Spencer, T., Reimherr, F. W., West, S. A., Allen, A. J., ... & Milton, D. (2003). Atomoxetine in adults with ADHD: two randomized, placebo-controlled studies. *Biological psychiatry*, *53*(2), 112-120.
176. Mick, E., Biederman, J., Spencer, T., Faraone, S. V., & Sklar, P. (2006). Absence of association with DAT1 polymorphism and response to methylphenidate in a sample of adults with ADHD. *American Journal of Medical Genetics Part B: Neuropsychiatric Genetics*, *141*(8), 890-894.
177. Mick, E., Faraone, S. V., Spencer, T., Zhang, H. F., & Biederman, J. (2008). Assessing the validity of the quality of life enjoyment and satisfaction questionnaire—short form in adults with ADHD. *Journal of attention disorders*, *11*(4), 504-509.
178. Mohammadzadeh, S., Ahangari, T. K., & Yousefi, F. (2019). The effect of memantine in adult patients with attention deficit hyperactivity disorder. *Human Psychopharmacology: Clinical and Experimental*, *34*(1), e2687.
179. Moorthy, G., Sallee, F., Gabbita, P., Zemlan, F., Sallans, L., & Desai, P. B. (2015). Safety, tolerability and pharmacokinetics of 2‐pyridylacetic acid, a major metabolite of betahistine, in a phase 1 dose escalation study in subjects with ADHD. *Biopharmaceutics & drug disposition*, *36*(7), 429-439.
180. Moritz, G. R., Pizutti, L. T., Cancian, A. C., Dillenburg, M. S., de Souza, L. A., Lewgoy, L. B., ... & Rohde, L. A. (2021). Feasibility trial of the dialectical behavior therapy skills training group as add‐on treatment for adults with attention‐deficit/hyperactivity disorder. *Journal of Clinical Psychology*, *77*(3), 516-524.
181. Mowinckel, A. M., Alnæs, D., Pedersen, M. L., Ziegler, S., Fredriksen, M., Kaufmann, T., ... & Biele, G. (2017). Increased default-mode variability is related to reduced task-performance and is evident in adults with ADHD. *NeuroImage: Clinical*, *16*, 369-382.
182. Nakashima, M., Inada, N., Tanigawa, Y., Yamashita, M., Maeda, E., Kouguchi, M., ... & Kuroki, T. (2022). Efficacy of group cognitive behavior therapy targeting time management for adults with attention deficit/hyperactivity disorder in Japan: a randomized control pilot trial. *Journal of Attention Disorders*, *26*(3), 377-390.
183. Nasser, A., Hull, J. T., Chaturvedi, S. A., Liranso, T., Odebo, O., Kosheleff, A. R., ... & Childress, A. (2022). A phase III, randomized, double-blind, placebo-controlled trial assessing the efficacy and safety of viloxazine extended-release capsules in adults with attention-deficit/hyperactivity disorder. *CNS drugs*, *36*(8), 897-915.
184. Naya, N., Sakai, C., Okutsu, D., Kiguchi, R., Fujiwara, M., Tsuji, T., & Iwanami, A. (2021). Efficacy and safety of guanfacine extended‐release in Japanese adults with attention‐deficit/hyperactivity disorder: Exploratory post hoc subgroup analyses of a randomized, double‐blind, placebo‐controlled study. *Neuropsychopharmacology reports*, *41*(1), 26-39.
185. Newcorn, J. H., Ivanov, I., Krone, B., Li, X., Duhoux, S., White, S., ... & Blair, R. J. (2024). Neurobiological basis of reinforcement-based decision making in adults with ADHD treated with lisdexamfetamine dimesylate: Preliminary findings and implications for mechanisms influencing clinical improvement. *Journal of Psychiatric Research*, *170*, 19-26.
186. Ni, H. C., Hwang Gu, S. L., Lin, H. Y., Lin, Y. J., Yang, L. K., Huang, H. C., & Gau, S. S. F. (2016). Atomoxetine could improve intra-individual variability in drug-naïve adults with attention-deficit/hyperactivity disorder comparably with methylphenidate: a head-to-head randomized clinical trial. *Journal of Psychopharmacology*, *30*(5), 459-467.
187. Ni, H. C., Shang, C. Y., Gau, S. S. F., Lin, Y. J., Huang, H. C., & Yang, L. K. (2013). A head-to-head randomized clinical trial of methylphenidate and atomoxetine treatment for executive function in adults with attention-deficit hyperactivity disorder. *International Journal of Neuropsychopharmacology*, *16*(9), 1959-1973.
188. Notzon, D. P., Mariani, J. J., Pavlicova, M., Glass, A., Mahony, A. L., Brooks, D. J., ... & Levin, F. R. (2016). Mixed‐amphetamine salts increase abstinence from marijuana in patients with co‐occurring attention‐deficit/hyperactivity disorder and cocaine dependence. *The American journal on addictions*, *25*(8), 666-672.
189. Nunes, E. V., Covey, L. S., Brigham, G., Hu, M. C., Levin, F. R., Somoza, E. C., & Winhusen, T. M. (2013). Treating nicotine dependence by targeting attention-deficit/hyperactivity disorder (ADHD) with OROS methylphenidate: the role of baseline ADHD severity and treatment response. *The Journal of clinical psychiatry*, *74*(10), 21297.
190. Pan, M. R., Dong, M., Zhang, S. Y., Liu, L., Li, H. M., Wang, Y. F., & Qian, Q. J. (2024). One-year follow-up of the effectiveness and mediators of cognitive behavioural therapy among adults with attention-deficit/hyperactivity disorder: secondary outcomes of a randomised controlled trial. *BMC psychiatry*, *24*(1), 207.
191. Pan, M. R., Huang, F., Zhao, M. J., Wang, Y. F., Wang, Y. F., & Qian, Q. J. (2019). A comparison of efficacy between cognitive behavioral therapy (CBT) and CBT combined with medication in adults with attention-deficit/hyperactivity disorder (ADHD). *Psychiatry research*, *279*, 23-33.
192. Pan, M. R., Zhang, S. Y., Qiu, S. W., Liu, L., Li, H. M., Zhao, M. J., ... & Qian, Q. J. (2022). Efficacy of cognitive behavioural therapy in medicated adults with attention-deficit/hyperactivity disorder in multiple dimensions: a randomised controlled trial. *European Archives of Psychiatry and Clinical Neuroscience*, 1-21.
193. Pan, M. R., Zhao, M. J., Liu, L., Li, H. M., Wang, Y. F., & Qian, Q. J. (2020). Cognitive behavioural therapy in groups for medicated adults with attention deficit hyperactivity disorder: protocol for a randomised controlled trial. *BMJ open*, *10*(10), e037514.
194. Paterson, R., Douglas, C., Hallmayer, J., Hagan, M., & Krupenia, Z. (1999). A randomised, double-blind, placebo-controlled trial of dexamphetamine in adults with attention deficit hyperactivity disorder. *Australian & New Zealand Journal of Psychiatry*, *33*(4), 494-502.
195. Paz, Y., Friedwald, K., Levkovitz, Y., Zangen, A., Alyagon, U., Nitzan, U., ... & Bloch, Y. (2018). Randomised sham-controlled study of high-frequency bilateral deep transcranial magnetic stimulation (dTMS) to treat adult attention hyperactive disorder (ADHD): Negative results. *The World Journal of Biological Psychiatry*, *19*(7), 561-566.
196. Pettersson, R., Söderström, S., Edlund-Söderström, K., & Nilsson, K. W. (2017). Internet-based cognitive behavioral therapy for adults with ADHD in outpatient psychiatric care: A randomized trial. *Journal of attention disorders*, *21*(6), 508-521.
197. Philipsen, A., Graf, E., Jans, T., Matthies, S., Borel, P., Colla, M., ... & Berger, M. (2014). A randomized controlled multicenter trial on the multimodal treatment of adult attention-deficit hyperactivity disorder: enrollment and characteristics of the study sample. *ADHD Attention Deficit and Hyperactivity Disorders*, *6*, 35-47.
198. Philipsen, A., Jans, T., Graf, E., Matthies, S., Borel, P., Colla, M., ... & van Elst, L. T. (2015). Effects of group psychotherapy, individual counseling, methylphenidate, and placebo in the treatment of adult attention-deficit/hyperactivity disorder: a randomized clinical trial. *JAMA psychiatry*, *72*(12), 1199-1210.
199. Potter, A. S., & Newhouse, P. A. (2008). Acute nicotine improves cognitive deficits in young adults with attention-deficit/hyperactivity disorder. *Pharmacology Biochemistry and Behavior*, *88*(4), 407-417.
200. Potter, A. S., Dunbar, G., Mazzulla, E., Hosford, D., & Newhouse, P. A. (2014). AZD3480, a novel nicotinic receptor agonist, for the treatment of attention-deficit/hyperactivity disorder in adults. *Biological psychiatry*, *75*(3), 207-214.
201. Potter, A. S., Ryan, K. K., & Newhouse, P. A. (2009). Effects of acute ultra‐low dose mecamylamine on cognition in adult attention‐deficit/hyperactivity disorder (ADHD). *Human Psychopharmacology: Clinical and Experimental*, *24*(4), 309-317.
202. Reimherr, F. W., Gift, T. E., Steans, T. A., Reimherr, M. L., Rosenberg, L. I., Wilson, M., & Marchant, B. K. (2022). The use of brexpiprazole combined with a stimulant in adults with treatment-resistant attention-deficit/hyperactivity disorder. *Journal of Clinical Psychopharmacology*, *42*(5), 445-453.
203. Reimherr, F. W., Marchant, B. K., Strong, R. E., Hedges, D. W., Adler, L., Spencer, T. J., ... & Soni, P. (2005). Emotional dysregulation in adult ADHD and response to atomoxetine. *Biological psychiatry*, *58*(2), 125-131.
204. Reimherr, F. W., Williams, E. D., Strong, R. E., Mestas, R., Soni, P., & Marchant, B. K. (2007). A double-blind, placebo-controlled, crossover study of osmotic release oral system methylphenidate in adults with ADHD with assessment of oppositional and emotional dimensions of the disorder. *Journal of Clinical Psychiatry*, *68*(1), 93-101.
205. Retz, W., Rösler, M., Ose, C., Scherag, A., Alm, B., Philipsen, A., ... & Study Group. (2012). Multiscale assessment of treatment efficacy in adults with ADHD: a randomized placebo-controlled, multi-centre study with extended-release methylphenidate. *The World Journal of Biological Psychiatry*, *13*(1), 48-59.
206. Riahi, F., Tehrani‐Doost, M., Shahrivar, Z., & Alaghband‐Rad, J. (2010). Efficacy of reboxetine in adults with attention‐deficit/hyperactivity disorder: A randomized, placebo‐controlled clinical trial. *Human Psychopharmacology: Clinical and Experimental*, *25*(7‐8), 570-576.
207. Rivkin, A., Alexander, R. C., Knighton, J., Hutson, P. H., Wang, X. J., Snavely, D. B., ... & Adler, L. A. (2012). A randomized, double-blind, crossover comparison of MK-0929 and placebo in the treatment of adults with ADHD. *Journal of Attention Disorders*, *16*(8), 664-674.
208. Rösler, M., Fischer, R., Ammer, R., Ose, C., Retz, W., & study group. (2009). A randomised, placebo-controlled, 24-week, study of low-dose extended-release methylphenidate in adults with attention-deficit/hyperactivity disorder. *European archives of psychiatry and clinical neuroscience*, *259*, 120-129.
209. Rösler, M., Retz, W., Fischer, R., Ose, C., Alm, B., Deckert, J., ... & Ammer, R. (2010). Twenty-four-week treatment with extended release methylphenidate improves emotional symptoms in adult ADHD. *The World Journal of Biological Psychiatry*, *11*(5), 709-718.
210. Safren, S. A., Duran, P., Yovel, I., Perlman, C. A., & Sprich, S. (2007). Medication adherence in psychopharmacologically treated adults with ADHD. *Journal of attention disorders*, *10*(3), 257-260.
211. Safren, S. A., Otto, M. W., Sprich, S., Winett, C. L., Wilens, T. E., & Biederman, J. (2005). Cognitive-behavioral therapy for ADHD in medication-treated adults with continued symptoms. *Behaviour research and therapy*, *43*(7), 831-842.
212. Safren, S. A., Sprich, S., Mimiaga, M. J., Surman, C., Knouse, L., Groves, M., & Otto, M. W. (2010). Cognitive behavioral therapy vs relaxation with educational support for medication-treated adults with ADHD and persistent symptoms: a randomized controlled trial. *Jama*, *304*(8), 875-880.
213. Salmi, J., Soveri, A., Salmela, V., Alho, K., Leppämäki, S., Tani, P., ... & Laine, M. (2020). Working memory training restores aberrant brain activity in adult attention‐deficit hyperactivity disorder. *Human Brain Mapping*, *41*(17), 4876-4891.
214. Schoenberg, P. L., Hepark, S., Kan, C. C., Barendregt, H. P., Buitelaar, J. K., & Speckens, A. E. (2014). Effects of mindfulness-based cognitive therapy on neurophysiological correlates of performance monitoring in adult attention-deficit/hyperactivity disorder. *Clinical neurophysiology*, *125*(7), 1407-1416.
215. Schönenberg, M., Wiedemann, E., Schneidt, A., Scheeff, J., Logemann, A., Keune, P. M., & Hautzinger, M. (2017). Neurofeedback, sham neurofeedback, and cognitive-behavioural group therapy in adults with attention-deficit hyperactivity disorder: a triple-blind, randomised, controlled trial. *The Lancet Psychiatry*, *4*(9), 673-684.
216. Schulz, K. P., Krone, B., Adler, L. A., Bédard, A. C. V., Duhoux, S., Pedraza, J., ... & Newcorn, J. H. (2018). Lisdexamfetamine targets amygdala mechanisms that bias cognitive control in attention-deficit/hyperactivity disorder. *Biological Psychiatry: Cognitive Neuroscience and Neuroimaging*, *3*(8), 686-693.
217. Selaskowski, B., Steffens, M., Schulze, M., Lingen, M., Aslan, B., Rosen, H., ... & Braun, N. (2022). Smartphone-assisted psychoeducation in adult attention-deficit/hyperactivity disorder: a randomized controlled trial. *Psychiatry Research*, *317*, 114802.
218. Sethi, A., Voon, V., Critchley, H. D., Cercignani, M., & Harrison, N. A. (2018). A neurocomputational account of reward and novelty processing and effects of psychostimulants in attention deficit hyperactivity disorder. *Brain*, *141*(5), 1545-1557.
219. Sobanski, E., Retz, W., Fischer, R., Ose, C., Alm, B., Hennig, O., & Rösler, M. (2014). Treatment adherence and persistence in adult ADHD: results from a twenty-four week controlled clinical trial with extended release methylphenidate. *European psychiatry*, *29*(5), 324-330.
220. Sobanski, E., Schredl, M., Kettler, N., & Alm, B. (2008). Sleep in adults with attention deficit hyperactivity disorder (ADHD) before and during treatment with methylphenidate: a controlled polysomnographic study. *Sleep*, *31*(3), 375-381.
221. Solanto, M. V., Marks, D. J., Wasserstein, J., Mitchell, K., Abikoff, H., Alvir, J. M. J., & Kofman, M. D. (2010). Efficacy of meta-cognitive therapy for adult ADHD. *American Journal of Psychiatry*, *167*(8), 958-968.
222. Solanto, M. V., Surman, C. B., & Alvir, J. M. J. (2018). The efficacy of cognitive–behavioral therapy for older adults with ADHD: a randomized controlled trial. *ADHD Attention Deficit and Hyperactivity Disorders*, *10*, 223-235.
223. Sörös, P., Hoxhaj, E., Borel, P., Sadohara, C., Feige, B., Matthies, S., ... & Philipsen, A. (2019). Hyperactivity/restlessness is associated with increased functional connectivity in adults with ADHD: a dimensional analysis of resting state fMRI. *BMC psychiatry*, *19*, 1-11.
224. Spencer, T. J., Adler, L. A., McGough, J. J., Muniz, R., Jiang, H., Pestreich, L., & Adult ADHD Research Group. (2007). Efficacy and safety of dexmethylphenidate extended-release capsules in adults with attention-deficit/hyperactivity disorder. *Biological Psychiatry*, *61*(12), 1380-1387.
225. Spencer, T. J., Adler, L. A., Weisler, R. H., & Youcha, S. H. (2008). Triple-bead mixed amphetamine salts (SPD465), a novel, enhanced extended-release amphetamine formulation for the treatment of adults with ADHD: a randomized, double-blind, multicenter, placebo-controlled study. *Journal of Clinical Psychiatry*, *69*(9), 1437.
226. Spencer, T. J., Bhide, P., Zhu, J., Faraone, S. V., Fitzgerald, M., Yule, A. M., ... & Biederman, J. (2017). Opiate antagonists do not interfere with the clinical benefits of stimulants in ADHD: a double-blind, placebo-controlled trial of the mixed opioid receptor antagonist naltrexone. *The Journal of clinical psychiatry*, *79*(1), 13465.
227. Spencer, T. J., Faraone, S. V., Michelson, D., Adler, L. A., Reimherr, F. W., Glatt, S. J., & Biederman, J. (2006). Atomoxetine and adult attention-deficit/hyperactivity disorder: the effects of comorbidity. *Journal of Clinical Psychiatry*, *67*(3), 415-420.
228. Spencer, T. J., Mick, E., Surman, C. B., Hammerness, P., Doyle, R., Aleardi, M., ... & Biederman, J. (2011). A randomized, single-blind, substitution study of OROS methylphenidate (Concerta) in ADHD adults receiving immediate release methylphenidate. *Journal of attention disorders*, *15*(4), 286-294.
229. Spencer, T., Biederman, J., Wilens, T., Doyle, R., Surman, C., Prince, J., ... & Faraone, S. (2005). A large, double-blind, randomized clinical trial of methylphenidate in the treatment of adults with attention-deficit/hyperactivity disorder. *Biological psychiatry*, *57*(5), 456-463.
230. Spencer, T., Biederman, J., Wilens, T., Faraone, S., Prince, J., Gerard, K., ... & Bearman, S. K. (2001). Efficacy of a mixed amphetamine salts compound in adults with attention-deficit/hyperactivity disorder. *Archives of general psychiatry*, *58*(8), 775-782.
231. Spencer, T., Biederman, J., Wilens, T., Prince, J., Hatch, M., Jones, J., ... & Seidman, L. (1998). Effectiveness and tolerability of tomoxetine in adults with attention deficit hyperactivity disorder. *American Journal of Psychiatry*, *155*(5), 693-695.
232. Spencer, T., Wilens, T., Biederman, J., Faraone, S. V., Ablon, J. S., & Lapey, K. (1995). A double-blind, crossover comparison of methylphenidate and placebo in adults with childhood-onset attention-deficit hyperactivity disorder. *Archives of General Psychiatry*, *52*(6), 434-443.
233. Stern, A., Malik, E., Pollak, Y., Bonne, O., & Maeir, A. (2016). The efficacy of computerized cognitive training in adults with ADHD: A randomized controlled trial. *Journal of attention disorders*, *20*(12), 991-1003.
234. Stern, P., Kolodny, T., Tsafrir, S., Cohen, G., & Shalev, L. (2023). Near and far transfer effects of computerized progressive attention training (CPAT) versus mindfulness based stress reduction (MBSR) practice among adults with ADHD. *Journal of Attention Disorders*, *27*(7), 757-776.
235. Stevenson, C. S., Whitmont, S., Bornholt, L., Livesey, D., & Stevenson, R. J. (2002). A cognitive remediation programme for adults with attention deficit hyperactivity disorder. *Australian & New Zealand Journal of Psychiatry*, *36*(5), 610-616.
236. Surman, C. B., Robertson, B., Chen, J., & Cortese, S. (2019). Post-hoc analyses of the effects of baseline sleep quality on SHP465 mixed amphetamine salts extended-release treatment response in adults with attention-deficit/hyperactivity disorder. *CNS drugs*, *33*, 695-706.
237. Surman, C. B., Walsh, D. M., Horick, N., DiSalvo, M., Vater, C. H., & Kaufman, D. (2023). Solriamfetol for attention-deficit/hyperactivity disorder in adults: a double-blind placebo-controlled pilot study. *The Journal of Clinical Psychiatry*, *84*(6), 49350.
238. Surman, C., Ceranoglu, A., Vaudreuil, C., Albright, B., Uchida, M., Yule, A., ... & Biederman, J. (2019). Does L-methylfolate supplement methylphenidate pharmacotherapy in attention-deficit/hyperactivity disorder?: Evidence of lack of benefit from a double-blind, placebo-controlled, randomized clinical trial. *Journal of clinical psychopharmacology*, *39*(1), 28-38.
239. Sutherland, S. M., Adler, L. A., Chen, C., Smith, M. D., & Feltner, D. E. (2012). An 8-week, randomized controlled trial of atomoxetine, atomoxetine plus buspirone, or placebo in adults with ADHD. *The Journal of clinical psychiatry*, *73*(4), 22316.
240. Takahashi, N., Koh, T., Tominaga, Y., Saito, Y., Kashimoto, Y., & Matsumura, T. (2014). A randomized, double-blind, placebo-controlled, parallel-group study to evaluate the efficacy and safety of osmotic-controlled release oral delivery system methylphenidate HCl in adults with attention-deficit/hyperactivity disorder in Japan. *The World Journal of Biological Psychiatry*, *15*(6), 488-498.
241. Taylor, F. B., & Russo, J. (2000). Efficacy of modafinil compared to dextroamphetamine for the treatment of attention deficit hyperactivity disorder in adults. *Journal of child and adolescent psychopharmacology*, *10*(4), 311-320.
242. Taylor, F. B., & Russo, J. (2001). Comparing guanfacine and dextroamphetamine for the treatment of adult attention-deficit/hyperactivity disorder. *Journal of clinical psychopharmacology*, *21*(2), 223-228.
243. Tenenbaum, S., Paull, J. C., Sparrow, E. P., Dodd, D. K., & Green, L. (2002). An experimental comparison of Pycnogenol® and methylphenidate in adults with Attention-Deficit/Hyperactivity Disorder (ADHD). *Journal of attention disorders*, *6*(2), 49-60.
244. Tolonen, T., Leppämäki, S., Roine, T., Alho, K., Tani, P., Koski, A., ... & Salmi, J. (2024). Working memory related functional connectivity in adult ADHD and its amenability to training: A randomized controlled trial. *NeuroImage: Clinical*, *44*, 103696.
245. Turner, D. C., Blackwell, A. D., Dowson, J. H., McLean, A., & Sahakian, B. J. (2005). Neurocognitive effects of methylphenidate in adult attention-deficit/hyperactivity disorder. *Psychopharmacology*, *178*, 286-295.
246. Turner, D. C., Clark, L., Dowson, J., Robbins, T. W., & Sahakian, B. J. (2004). Modafinil improves cognition and response inhibition in adult attention-deficit/hyperactivity disorder. *Biological psychiatry*, *55*(10), 1031-1040.
247. Upadhyaya, H., Tanaka, Y., Lipsius, S., Kryzhanovskaya, L. A., Lane, J. R., Escobar, R., ... & Allen, A. J. (2015). Time-to-onset and-resolution of adverse events before/after atomoxetine discontinuation in adult patients with ADHD. *Postgraduate medicine*, *127*(7), 677-685.
248. van Andel, E., Bijlenga, D., Vogel, S. W., Beekman, A. T., & Kooij, J. S. (2021). Effects of chronotherapy on circadian rhythm and ADHD symptoms in adults with attention-deficit/hyperactivity disorder and delayed sleep phase syndrome: a randomized clinical trial. *Chronobiology international*, *38*(2), 260-269.
249. van Andel, E., Vogel, S. W., Bijlenga, D., Kalsbeek, A., Beekman, A. T., & Kooij, J. S. (2024). Effects of Chronotherapeutic Interventions in Adults With ADHD and Delayed Sleep Phase Syndrome (DSPS) on Regulation of Appetite and Glucose Metabolism. *Journal of attention disorders*, *28*(13), 1653-1667.
250. Van der Oord, S., Boyer, B. E., Van Dyck, L., Mackay, K. J., De Meyer, H., & Baeyens, D. (2020). A randomized controlled study of a cognitive behavioral planning intervention for college students with ADHD: An effectiveness study in student counseling services in Flanders. *Journal of Attention Disorders*, *24*(6), 849-862.
251. Vansickel, A. R., Stoops, W. W., Glaser, P. E., Poole, M. M., & Rush, C. R. (2011). Methylphenidate increases cigarette smoking in participants with ADHD. *Psychopharmacology*, *218*, 381-390.
252. Verster, J. C., & Roth, T. (2014). Methylphenidate significantly reduces lapses of attention during on-road highway driving in patients with ADHD. *Journal of clinical psychopharmacology*, *34*(5), 633-636.
253. Verster, J. C., Bekker, E. M., De Roos, M., Minova, A., Eijken, E. J., Kooij, J. S., ... & Volkerts, E. R. (2008). Methylphenidate significantly improves driving performance of adults with attention-deficit hyperactivity disorder: a randomized crossover trial. *Journal of Psychopharmacology*, *22*(3), 230-237.
254. Verster, J. C., Bekker, E. M., Kooij, J. S., Buitelaar, J. K., Verbaten, M. N., Volkerts, E. R., & Olivier, B. (2010). Methylphenidate significantly improves declarative memory functioning of adults with ADHD. *Psychopharmacology*, *212*, 277-281.
255. Vidal, R., Bosch, R., Nogueira, M., Gómez-Barros, N., Valero, S., Palomar, G., ... & Ramos-Quiroga, J. A. (2013). Psychoeducation for adults with attention deficit hyperactivity disorder vs. cognitive behavioral group therapy: a randomized controlled pilot study. *The Journal of nervous and mental disease*, *201*(10), 894-900.
256. Wang, Z., Zuschlag, Z. D., Myers, U. S., & Hamner, M. (2022). Atomoxetine in comorbid ADHD/PTSD: a randomized, placebo controlled, pilot, and feasibility study. *Depression and Anxiety*, *39*(4), 286-295.
257. Weisler, R. H., Biederman, J., Spencer, T. J., & Wilens, T. E. (2005). Long-term cardiovascular effects of mixed amphetamine salts extended release in adults with ADHD. *CNS spectrums*, *10*(S20), 35-43.
258. Weisler, R. H., Biederman, J., Spencer, T. J., Wilens, T. E., Faraone, S. V., Chrisman, A. K., ... & Tulloch, S. J. (2006). Mixed amphetamine salts extended-release in the treatment of adult ADHD: a randomized, controlled trial. *Cns Spectrums*, *11*(8), 625-639.
259. Weisler, R. H., Greenbaum, M., Arnold, V., Yu, M., Yan, B., Jaffee, M., & Robertson, B. (2017). Efficacy and safety of SHP465 mixed amphetamine salts in the treatment of attention-deficit/hyperactivity disorder in adults: results of a randomized, double-blind, placebo-controlled, forced-dose clinical study. *CNS drugs*, *31*, 685-697.
260. Weisler, R. H., Pandina, G. J., Daly, E. J., Cooper, K., & Gassmann-Mayer, C. (2012). Randomized clinical study of a histamine H 3 receptor antagonist for the treatment of adults with attention-deficit hyperactivity disorder. *CNS drugs*, *26*, 421-434.
261. Weisler, R., Ginsberg, L., Dirks, B., Deas, P., Adeyi, B., & Adler, L. A. (2017). Treatment with lisdexamfetamine dimesylate improves self-and informant-rated executive function behaviors and clinician-and informant-rated ADHD symptoms in adults: data from a randomized, double-blind, placebo-controlled study. *Journal of Attention Disorders*, *21*(14), 1198-1207.
262. Weisler, R., Young, J., Mattingly, G., Gao, J., Squires, L., & Adler, L. (2009). Long-term safety and effectiveness of lisdexamfetamine dimesylate in adults with attention-deficit/hyperactivity disorder. *CNS spectrums*, *14*(10), 573-586.
263. Weiss, M. D., Childress, A. C., & Donnelly, G. A. (2021). Efficacy and safety of PRC-063, extended-release multilayer methylphenidate in adults with ADHD including 6-month open-label extension. *Journal of Attention Disorders*, *25*(10), 1417-1428.
264. Weiss, M. D., Surman, C., Khullar, A., He, E., Cataldo, M., & Donnelly, G. (2021). Effect of a multi-layer, extended-release methylphenidate formulation (PRC-063) on sleep in adults with ADHD: a randomized, double-blind, forced-dose, placebo-controlled trial followed by a 6-month open-label extension. *CNS drugs*, *35*(6), 667-679.
265. Weiss, M., Hechtman, L., & Adult ADHD Research Group. (2006). A randomized double-blind trial of paroxetine and/or dextroamphetamine and problem-focused therapy for attention-deficit/hyperactivity disorder in adults. *Journal of Clinical Psychiatry*, *67*(4), 611-619.
266. Weiss, M., Murray, C., Wasdell, M., Greenfield, B., Giles, L., & Hechtman, L. (2012). A randomized controlled trial of CBT therapy for adults with ADHD with and without medication. *BMC psychiatry*, *12*, 1-8.
267. Wender, P. H., Reimherr, F. W., & Wood, D. R. (1981). Attention deficit disorder ('minimal brain dysfunction') in adults: A replication study of diagnosis and drug treatment. *Archives of General Psychiatry*, *38*(4), 449-456.
268. Wender, P. H., Reimherr, F. W., Marchant, B. K., Sanford, M. E., Czajkowski, L. A., & Tomb, D. A. (2011). A one year trial of methylphenidate in the treatment of ADHD. *Journal of Attention Disorders*, *15*(1), 36-45.
269. Wender, P. H., Reimherr, F. W., Wood, D., & Ward, M. (1985). A controlled study of methylphenidate in the treatment of attention deficit disorder, residual type, in adults. *Journal of Clinical Psychopharmacology*, *5*(5), 306-307.
270. Wietecha, L., Young, J., Ruff, D., Dunn, D., Findling, R. L., & Saylor, K. (2012). Atomoxetine once daily for 24 weeks in adults with attention-deficit/hyperactivity disorder (ADHD): impact of treatment on family functioning. *Clinical neuropharmacology*, *35*(3), 125-133.
271. Wigal, S. B., Wigal, T., Childress, A., Donnelly, G. A., & Reiz, J. L. (2020). The time course of effect of multilayer-release methylphenidate hydrochloride capsules: a randomized, double-blind study of adults with ADHD in a simulated adult workplace environment. *Journal of Attention Disorders*, *24*(3), 373-383.
272. Wigal, T. L., Newcorn, J. H., Handal, N., Wigal, S. B., Mulligan, I., Schmith, V., & Konofal, E. (2018). A double-blind, placebo-controlled, phase II study to determine the efficacy, safety, tolerability and pharmacokinetics of a controlled release (CR) formulation of mazindol in adults with DSM-5 attention-deficit/hyperactivity disorder (ADHD). *CNS drugs*, *32*, 289-301.
273. Wigal, T., Brams, M., Frick, G., Yan, B., & Madhoo, M. (2018). A randomized, double-blind study of SHP465 mixed amphetamine salts extended-release in adults with ADHD using a simulated adult workplace design. *Postgraduate Medicine*, *130*(5), 481-493.
274. Wigal, T., Brams, M., Gasior, M., Gao, J., & Giblin, J. (2011). Effect size of lisdexamfetamine dimesylate in adults with attention-deficit/hyperactivity disorder. *Postgraduate medicine*, *123*(2), 169-176.
275. Wigal, T., Brams, M., Gasior, M., Gao, J., Squires, L., Giblin, J., & 316 Study Group. (2010). Randomized, double-blind, placebo-controlled, crossover study of the efficacy and safety of lisdexamfetamine dimesylate in adults with attention-deficit/hyperactivity disorder: novel findings using a simulated adult workplace environment design. *Behavioral and Brain Functions*, *6*, 1-14.
276. Wigal, T., Childress, A., Frick, G., Yan, B., Wigal, S., & Madhoo, M. (2018). Effects of SHP465 mixed amphetamine salts in adults with ADHD in a simulated adult workplace environment. *Postgraduate Medicine*, *130*(1), 111-121.
277. Wilens, T. E., Adler, L. A., Tanaka, Y., Xiao, F., D’Souza, D. N., Gutkin, S. W., & Upadhyaya, H. P. (2011). Correlates of alcohol use in adults with ADHD and comorbid alcohol use disorders: exploratory analysis of a placebo-controlled trial of atomoxetine. *Current medical research and opinion*, *27*(12), 2309-2320.
278. Wilens, T. E., Adler, L. A., Weiss, M. D., Michelson, D., Ramsey, J. L., Moore, R. J., ... & Atomoxetine ADHD/SUD Study Group. (2008). Atomoxetine treatment of adults with ADHD and comorbid alcohol use disorders. *Drug and alcohol dependence*, *96*(1-2), 145-154.
279. Wilens, T. E., Biederman, J., Prince, J., Spencer, T. J., Faraone, S. V., Warburton, R., ... & Geller, D. (1996). Six-week, double-blind, placebo-controlled study of desipramine for adult attention deficit hyperactivity disorder. *American Journal of Psychiatry*, *153*(9), 1147-1153.
280. Wilens, T. E., Biederman, J., Spencer, T. J., Bostic, J., Prince, J., Monuteaux, M. C., ... & Polisner, D. (1999). A pilot controlled clinical trial of ABT-418, a cholinergic agonist, in the treatment of adults with attention deficit hyperactivity disorder. *American Journal of Psychiatry*, *156*(12), 1931-1937.
281. Wilens, T. E., Biederman, J., Spencer, T. J., Frazier, J., Prince, J., Bostic, J., ... & Abrantes, A. (1999). Controlled trial of high doses of pemoline for adults with attention-deficit/hyperactivity disorder. *Journal of Clinical Psychopharmacology*, *19*(3), 257-264.
282. Wilens, T. E., Haight, B. R., Horrigan, J. P., Hudziak, J. J., Rosenthal, N. E., Connor, D. F., ... & Modell, J. G. (2005). Bupropion XL in adults with attention-deficit/hyperactivity disorder: a randomized, placebo-controlled study. *Biological psychiatry*, *57*(7), 793-801.
283. Wilens, T. E., Hammerness, P. G., Biederman, J., Kwon, A., Spencer, T. J., Clark, S., ... & Moore, H. (2005). Blood pressure changes associated with medication treatment of adults with attention-deficit/hyperactivity disorder. *Journal of clinical psychiatry*, *66*(2), 253-259.
284. Wilens, T. E., Spencer, T. J., Biederman, J., Girard, K., Doyle, R., Prince, J., ... & Parekh, A. (2001). A controlled clinical trial of bupropion for attention deficit hyperactivity disorder in adults. *American Journal of Psychiatry*, *158*(2), 282-288.
285. Wilens, T. E., Verlinden, M. H., Adler, L. A., Wozniak, P. J., & West, S. A. (2006). ABT-089, a neuronal nicotinic receptor partial agonist, for the treatment of attention-deficit/hyperactivity disorder in adults: results of a pilot study. *Biological psychiatry*, *59*(11), 1065-1070.
286. Winhusen, T. M., Somoza, E. C., Brigham, G. S., Liu, D. S., Green, C. A., Covey, L. S., ... & Dorer, E. M. (2010). Impact of attention-deficit/hyperactivity disorder (ADHD) treatment on smoking cessation intervention in ADHD smokers: a randomized, double-blind, placebo-controlled trial. *The Journal of clinical psychiatry*, *71*(12), 8530.
287. Wood, D. R., Reimherr, F. W., Wender, P. H., & Johnson, G. E. (1976). Diagnosis and treatment of minimal brain dysfunction in adults: a preliminary report. *Archives of general psychiatry*, *33*(12), 1453-1460.
288. Yang, Z., Kelly, C., Castellanos, F. X., Leon, T., Milham, M. P., & Adler, L. A. (2016). Neural correlates of symptom improvement following stimulant treatment in adults with attention-deficit/hyperactivity disorder. *Journal of child and adolescent psychopharmacology*, *26*(6), 527-536.
289. Young, J. L., Sarkis, E., Qiao, M., & Wietecha, L. (2011). Once-daily treatment with atomoxetine in adults with attention-deficit/hyperactivity disorder: a 24-week, randomized, double-blind, placebo-controlled trial. *Clinical neuropharmacology*, *34*(2), 51-60.
290. Young, S., Khondoker, M., Emilsson, B., Sigurdsson, J. F., Philipp-Wiegmann, F., Baldursson, G., ... & Gudjonsson, G. (2015). Cognitive–behavioural therapy in medication-treated adults with attention-deficit/hyperactivity disorder and co-morbid psychopathology: a randomized controlled trial using multi-level analysis. *Psychological medicine*, *45*(13), 2793-2804.
291. Zilverstand, A., Sorger, B., Slaats-Willemse, D., Kan, C. C., Goebel, R., & Buitelaar, J. K. (2017). fMRI neurofeedback training for increasing anterior cingulate cortex activation in adult attention deficit hyperactivity disorder. An exploratory randomized, single-blinded study. *PloS one*, *12*(1), e0170795.
292. Zimmermann, M., Grabemann, M., Mette, C., Abdel-Hamid, M., Ueckermann, J., Kraemer, M., ... & Zepf, F. D. (2012). The effects of acute tryptophan depletion on reactive aggression in adults with attention-deficit/hyperactivity disorder (ADHD) and healthy controls. *PloS one*, *7*(3), e32023.
